# Systematic Analysis of Compositional Order of Proteins Reveals New Characteristics of Biological Functions and a Universal Correlate of Macroevolution

**DOI:** 10.1371/journal.pcbi.1003346

**Published:** 2013-11-21

**Authors:** Erez Persi, David Horn

**Affiliations:** School of Physics and Astronomy, Tel Aviv University, Tel Aviv, Israel; Wake Forest University, United States of America

## Abstract

We present a novel analysis of compositional order (CO) based on the occurrence of Frequent amino-acid Triplets (FTs) that appear much more than random in protein sequences. The method captures all types of proteomic compositional order including single amino-acid runs, tandem repeats, periodic structure of motifs and otherwise low complexity amino-acid regions. We introduce new order measures, distinguishing between ‘regularity’, ‘periodicity’ and ‘vocabulary’, to quantify these phenomena and to facilitate the identification of evolutionary effects. Detailed analysis of representative species across the tree-of-life demonstrates that CO proteins exhibit numerous functional enrichments, including a wide repertoire of particular patterns of dependencies on regularity and periodicity. Comparison between human and mouse proteomes further reveals the interplay of CO with evolutionary trends, such as faster substitution rate in mouse leading to decrease of periodicity, while innovation along the human lineage leads to larger regularity. Large-scale analysis of 94 proteomes leads to systematic ordering of all major taxonomic groups according to FT-vocabulary size. This is measured by the count of Different Frequent Triplets (DFT) in proteomes. The latter provides a clear hierarchical delineation of vertebrates, invertebrates, plants, fungi and prokaryotes, with thermophiles showing the lowest level of FT-vocabulary. Among eukaryotes, this ordering correlates with phylogenetic proximity. Interestingly, in all kingdoms CO accumulation in the proteome has universal characteristics. We suggest that CO is a genomic-information correlate of both macroevolution and various protein functions. The results indicate a mechanism of genomic ‘innovation’ at the peptide level, involved in protein elongation, shaped in a universal manner by mutational and selective forces.

## Author Summary

Variations in compositionally ordered (CO) sections of proteins, such as amino acid runs, tandem repeats and low complexity regions, are often considered as a third type of genomic variation along with SNP and CNV. At the microevolutionary scale, they are involved in the rapid evolution of numerous biological functions and the development of novel phenotypic complex traits, including disease in human, in particular neurodegeneration and cancer. At the macroevolutionary scale, the best discriminating proteomic factor between super-kingdoms is the prevalence of CO proteins in eukaryotes. The analysis of CO structures has so far been quite eclectic. Here we introduce a novel unifying methodology, accounting for all types of low-complexity regions and repetitive phenomena, including the existence of large periodic structures in protein sequences. We define new CO measures providing insights into the correlation of CO with protein function and with evolution. In particular, a large-scale analysis of 94 proteomes shows that the CO vocabulary of frequently appearing amino acid triplets serves as a measure of taxonomic ordering separating major clades from each other. It unravels a missing genomic correlate of macroevolution and serves as a novel phylogenetic tool. This suggests that major CO generation occurs during the creation of a completely new species, i.e. during macroevolutionary events.

## Introduction

Most protein sequences appear to be quite random. Nonetheless, many sequences display various types of ordered patterns, observed in all kingdoms of life [1], [2]. These include successive expansion of a single amino-acid (known as ‘run’ or homo-peptide), repetitive sections with various lengths and degree of purity, and more generally, low-complexity amino-acid regions, i.e., sections of high compositional bias manifested by low single amino-acid Shannon entropy [3]. We refer hereafter to the phenomena of ordered patterns in protein sequences as compositional order (CO). 

In the DNA, ordered patterns appear in both non-coding and coding regions, including minisatellites and microsatellites, or generally tandem repeats of chains of few nucleotides. Their generating mechanisms involve replication slippage and recombination effects [4]. These mechanisms, and others such as segmental duplications, may serve as the origin of the CO structures observed in proteins. Proteins containing CO exhibit a wide variety of functions associated with disordered, as well as ordered, 3D structures including extended coiled, helical domains, molten globules, collagen, keratin and zinc-fingers [5]–[8]. They are involved in DNA binding, alternative splicing, transcription, regulation, protein-protein interaction, tumor genesis [5], [8], [9], and formation of novel functions, such as cell envelopes of keratinocytes [10].

Tandem repeats are thought to represent a third type of genomic variation along with single nucleotide polymorphisms (SNPs) and copy number variation (CNV) [11]. This is because they are important not only for protein function but also for the fast evolution of complex traits, including various phenotypic and morphological changes as well as adaptive and social behaviors [12]–[14]. Variations of repeats in coding DNA sequences were found particularly important in some rapid evolutionary processes, such as changes in size and shape of limbs and craniums of dog [15], and fast adaptation to changing environment of cell wall proteins in yeast, which allows for avoiding capture by the host immune system [16]. In contrast, in human, variations in amino-acid runs have been associated with disease, in particular various cancers [17] and neurodegenerative diseases [18], where related proteins are rich with poly-Q repeats [19], poly-A repeats [20], or multiple runs of various amino-acids as in the case of the Huntingtin protein.

The dual association of repeats with both essential functions and disease promoted the view that repetitive sections are subjected to rapid evolution by fast mutational drive, which facilitates the acquisition of a function soon after a repetitive section came into being [5], [9]. The latter presumably did not fit any initial functionality and may have even contained a risk of leading to deleterious effects. This raised the question of which evolutionary forces act on repetitive sequences. Early studies [5], [9], [21] pointed out that interruptions of amino-acid runs are evidence of mutational forces, and that mutations at the third DNA synonymous site are indicative of selectivity of function. However, the role of selection remained elusive as other studies have shown that repeats are only weakly conserved across species, indicating weak selective pressure, except for some specific genes of the *xylanase* family, the heat response protein *Dnaj*, and the ribosomal *L10*, *12* proteins, which were identified as the origins of amino-acid runs in prokaryotes [22]. Recently it was shown that alternatively spliced exons are enriched in repeats with low codon-diversity [23], and that repeat conservation in vertebrates is three times higher in coding than in non-coding regions, but less in primates [24]. Both observations constitute strong evidence for selection. Evidently three fundamental evolutionary forces: mutation, selection and ‘innovation’, i.e. generation of new raw repetitive sequences exist; however, the balance between these forces is hard to measure and may vary considerably among species and conditions.

The interrelation between CO and evolution has been stressed by Albà et al. [25], who have suggested that repeats may have an important role in organism diversity and macroevolution, i.e., the generation of higher taxa. This is because some developmental genes, like Ubx in insects and HOX in human, are responsible for major organism-specific characteristics and are rich in homo-peptides. Indeed, previous analyses of a large ensemble of species revealed that the number of CO proteins is three times larger in eukaryotes than in prokaryotes, independently of protein length [26]. Furthermore, some CO proteins were associated with specific eukaryotic functions such as collagen, calcium binding and keratin. Thus it was suggested that eukaryotes favor the generation of repeats as a source of variability to compensate for their relatively slow evolutionary rate [26], [27], indicating that the mechanisms shaping CO are not universal in the super-kingdoms.

In the general framework of evolution, with particular emphasis on eukaryotes, it should be noted that species development is often described in terms of increasing organism complexity [28], which is thought to be reflected by several factors such as the numbers of different tissues, cell types, proteins and their interactions [29]. Attempts to quantify this complexity from genomic sequence suggest that natural selection is a necessary mechanism to explain the seemingly increase in biological complexity [30]. Nevertheless, the questions of which evolutionary forces participate in the development of complex traits, what is the balance between them, how it depends on environmental and ecological factors, and whether all this leaves any measurable genomic-information stamp that correlates with the evolutionary path of species complexity, remained unresolved [31], [32].

In this study we introduce the concept of Compositional Order (CO), accounting for all types of repetitive and low complexity regions. The novel framework is based on the identification and quantification of Frequent amino acid Triplets (FTs). The biological importance of both amino acid and DNA triplets has been pointed out in various studies, emphasizing their role in the characterization of major bacterial phyla and super-kingdoms [33], [34], and the evolutionary importance of their spontaneous expansions in higher taxa [35]. We show that triplets of amino acids are adequate and even optimal building blocks for a systematic characterization of CO. We define and exhibit three measures of CO in proteins: ‘regularity’, ‘periodicity’ and ‘vocabulary’. Regularity refers to the high multiplicity of amino-acid triplets, and is defined by the relative coverage of a protein's sequence by FTs. This measure is highly correlated with Shannon's entropy hence it recapitulates the conventional establishment of low sequence-complexity regions. Periodicity reflects the relative amount of FT occurrences within a periodic structure observed on the protein sequence. In the case of tandem repeats it may account for basic motif characterization. FT-vocabulary is defined as the number of observed Different Frequent Triplets (DFT) in either a single protein or in a full proteome.

We demonstrate and evaluate the phenomenology of CO in human proteins, quantifying them in detail using the new measures. We explore the functional enrichment of proteins containing CO in several representative species, emphasizing their dependencies on these new measures. We discuss the evolutionary interpretation of these dependencies. A comparative study of human *vs* mouse proteomes provides new insights on the interplay of CO with evolutionary forces. Last, we concentrate on a large-scale proteomic study, comparing 94 species from all kingdoms of life. This leads to the observation that FT-vocabulary is an important measure. At the proteome level DFT counts provide clear delineation of vertebrates, invertebrates, plants and fungi from each other, with bacteria and archaea closing the list, concluding that DFT is a universal proteomic marker of macroevolution. This throws new light on fundamental questions in the evolution of species and on the nature of the genomic mechanisms involved.

## Results

We define Frequent Triplets (FT) to be those amino-acid triplets that are observed in protein sequences far beyond random (see Methods). Specifically, we search for triplets that occur at least 5 times in a protein. Their statistical significance is discussed in Text S1 (section 1–2, figures S1, S2, S3, S4, S5). In Methods, we establish that the **relative coverage (RC)** of FTs in a protein sequence highly correlates with sequence entropy, providing a good tool for estimating ‘regularity’ (Text S1 - section 4, figure S7). Additionally, the intervals between the consecutive occurrences of an FT provide information about the existence of periodic structures on the protein's sequence. These are identified by the **most frequent interval (MFI)** encountered in a protein, chosen out of all intervals displayed by FT recurrences on the sequence. The level of ‘periodicity’ in a protein is then estimated by the **relative periodicity (RP)**: the sum of all FT recurrences at MFI divided by the sum of all FTs occurrences. Thus we obtain through FTs independent information about both prevalent composition and prevalent periodicities. Few representative examples are shown in Table 1. Complete detailed information is provided in Methods.

**Table 1:** Typical Examples of proteins containing FTs.

Protein	Length	# DFTs	Leading FT	MFI	RC, RP	Amino-acid sequence (leading FTs are highlighted)
**A. A4**Amyloid beta A4 protein	770	3	AEEEEETTT	1	0.04, 0.4	MLPGLALLLLAAWTARALEVPTDGNAGLLAEPQIAMFCGRLNMHMVQNGKWDSDPSGTKTCIDTKEGILQYCQEVYPELQITNVVEANQPVTIQNWCKRGRKQCKTHPHFVIPYRCLVGEFVSDALLVPDKCKFLHQERMDVCETHLHWHTVAKETCSEKSTNLHDYGMLLPCGIDKFRGVEFVCCPL**AEE**SDNVDSAD**AEE**DDSDVWWGGADTDYADGSEDKVVEV**AEEEE**VAEV**EEEE**ADDDEDDEDGDEV**EEEAEE**PYEEATERTTSIA**TTTTTTT**ESVEEVVREVCSEQAETGPCRAMISRWYFDVTEGKCAPFFYGGCGGNRNNFDTEEYCMAVCGSAMSQSLLKTTQEPLARDPVKLPTTAASTPDAVDKYLETPGDENEHAHFQKAKERLEAKHRERMSQVMREWEEAERQAKNLPKADKKAVIQHFQEKVESLEQEAANERQQLVETHMARVEAMLNDRRRLALENYITALQAVPPRPRHVFNMLKKYVRAEQKDRQHTLKHFEHVRMVDPKKAAQIRSQVMTHLRVIYERMNQSLSLLYNVPAV**AEE**IQDEVDELLQKEQNYSDDVLANMISEPRISYGNDALMPSLTETKTTVELLPVNGEFSLDDLQPWHSFGADSVPANTENEVEPVDARPAADRGLTTRPGSGLTNIKTEEISEVKMDAEFRHDSGYEVHHQKLVFFAEDVGSNKGAIIGLMVGGVVIATVIVITLVMLKKKQYTSIHHGVVEVDAAVTPEERHLSKMQQNGYENPTYKFFEQMQN
**B. LORI**Keratin	312	12	GGG	1	0.72, 0.13	MSYQKKQPTPQPPVDCVKTS**GGGGGGGG**S**GGGG**CGFF**GGGG**S**GGG**SSGSGCGYS**GGGG**YS**GGG**C**GGG**SS**GGGGGGG**IGGC**GGG**SGGSVKYS**GGGG**SS**GGG**SGCFSS**GGGG**SGCFSS**GGGG**SS**GGG**SGCFSS**GGGG**SS**GGG**SGCFSS**GGGG**FSGQAVQCQSYGGVSSGGSS**GGG**SGCFSS**GGGGG**SVCGYS**GGG**SGC**GGG**SSGGSGSGYVSSQQVTQTSCAPQPSY**GGG**SS**GGGG**SGGSGCFSS**GGGGG**SSGC**GGG**SSGIGSGCIIS**GGG**SVC**GGG**SS**GGGGGG**SSVGGSGSGKGVPICHQTQQKQAPTWPSK
**C. CAMKV**ATP binding	501	5	TPAPATATD	8	0.1, 0.69	MPFGCVTLGDKKNYNQPSEVTDRYDLGQVIKTEEFCEIFRAKDKTTGKLHTCKKFQKRDGRKVRKAAKNEIGILKMVKHPNILQLVDVFVTRKEYFIFLELATGREVFDWILDQGYYSERDTSNVVRQVLEAVAYLHSLKIVHRNLKLENLVYYNRLKNSKIVISDFHLAKLENGLIKEPCGTPEYLAPEVVGRQRYGRPVDCWAIGVIMYILLSGNPPFYEEVEEDDYENHDKNLFRKILAGDYEFDSPYWDDISQAAKDLVTRLMEVEQDQRITAEEAISHEWISGNAASDKNIKDGVCAQIEKNFARAKWKKAVRVTTLMKRLRAPEQSSTAAAQSAS**ATD**TATPGAAGGATAAAASGATSAPEGDAARAAKSDNVAPADRSA**TPATD**GSA**TPATD**GSV**TPATD**GSI**TPATD**GSV**TPATD**RSA**TPATD**GRA**TPAT**EESTVPTTQSSAMLATKAAATPEPAMAQPDSTAPEGATGQAPPSSKGEEAAGYAQESQREEAS
**D. COLQ**Collagen	455	7	PGP	6	0.18, 0.14	MVVLNPMTLGIYLQLFFLSIVSQPTFINSVLPISAALPSLDQKKRGGHKACCLLTPPPPPLFPPPFFRGGRSPLLSPDMKNLMLELETSQSPCMQGSLGS**PGPPGP**QGPPGLPGKTGPKGEKGELGRPGRKGR**PGP**PGVPGM**PGP**IGW**PGP**EGPRGEKGDLGMMGLPGSRGPMGSKGYPGSRGEKGSRGEKGDLGPKGEKGFPGFPGMLGQKGEMGPKGEPGIAGHRGPTGRPGKRGKQGQKGDSGVMGPPGK**PGP**SGQPGR**PGPPGP**PPAGQLIMGPKGERGF**PGP**PGRCLCGPTMNVNNPSYGESVYGPSSPRVPVIFVVNNQEELERLNTQNAIAFRRDQRSLYFKDSLGWLPIQLTPFYPVDYTADQHGTCGDGLLQPGEECDDGNSDVGDDCIRCHRAYCGDGHRHEGVEDCDGSDFGYLTCETYLPGSYGDLQCTQYCYIDSTPCRYFT
**E. ASPX**multi - cellular organismal development	265	6	SGE	5	0.24, 0.35	MNRFLLLMSLYLLGSARGTSSQPNELSGSIDHQTSVQQLPGEFFSLENPSDAEALYETSSGLNTLSEHGSSEHGSSKHTVAEHT**SGE**HAESEHA**SGE**PAATEHAEGEHTVGEQP**SGE**QP**SGE**HL**SGE**QPLSELE**SGE**QPSDEQP**SGE**HG**SGE**QP**SGE**QA**SGE**QP**SGE**HA**SGE**QASGAPISSTSTGTILNCYTCAYMNDQGKCLRGEGTCITQNSQQCMLKKIFEGGKLQFMVQGCENMCPSMNLFSHGTRMQIICCRNQSFCNKI
**F. PRDM9**Zinc-finger	894	28	HQRHTGTGEGEKYVCVCRCREECG	28	0.36, 0.84	MSPEKSQEESPEEDTERTERKPMVKDAFKDISIYFTKEEWAEMGDWEKTRYRNVKRNYNALITIGLRATRPAFMCHRRQAIKLQVDDTEDSDEEWTPRQQVKPPWMALRVEQRKHQKGMPKASFSNESSLKELSRTANLLNASGSEQAQKPVSPSGEASTSGQHSRLKLELRKKETERKMYSLRERKGHAYKEVSEPQDDDYLYCEMCQNFFIDSCAAHGPPTFVKDSAVDKGHPNRSALSLPPGLRIGPSGIPQAGLGVWNEASDLPLGLHFGPYEGRITEDEEAANNGYSWLITKGRNCYEYVDGKDKSWANWMRYVNCARDDEEQNLVAFQYHRQIFYRTCRVIRPGCELLVWYGDEYGQELGIKWGSKWKKELMAGREPKPEIHPCPSCCLAFSSQKFLSQHVERNHSSQNFPGPSARKLLQPENPCPGDQNQEQQYPDPHSRNDKTKGQEIKERSKLLNKRTWQREISRAFSSPPKGQMGSCRVGKRIMEEESRTGQKVNPGNTGKLFVGVGISRIAKVKYG**ECG**QGFSVKSDVIT**HQR**T**HTGEK**L**YVCRECG**RGFSWKSHLLI**HQR**I**HTGEK**P**YVCRECG**RGFSWQSVLLT**HQR**T**HTGEK**P**YVCRECG**RGFSRQSVLLT**HQR**R**HTGEK**P**YVCRECG**RGFSRQSVLLT**HQR**R**HTGEK**P**YVCRECG**RGFSWQSVLLT**HQR**T**HTGEK**P**YVCRECG**RGFSWQSVLLT**HQR**T**HTGEK**P**YVCRECG**RGFSNKSHLLR**HQR**T**HTGEK**P**YVCRECG**RGFRDKSHLLR**HQR**T**HTGEK**P**YVCRECG**RGFRDKSNLLS**HQR**T**HTGEK**P**YVCRECG**RGFSNKSHLLR**HQR**T**HTGEK**P**YVCRECG**RGFRNKSHLLR**HQR**T**HTGEK**P**YVCRECG**RGFSDRSSLCY**HQR**T**HTGEK**P**YVCRE**DE

Typical examples of order patterns, as obtained by FT search in the human proteome. For each protein, Swiss-Prot entry name and main function is given in the first column, and then follow the protein length, the number of different frequent-triplets (DFT), the leading FTs, defined by the maximal number of occurrences of a FT, and the CO measures MFI, RC, RP. The leading FTs are highlighted within the protein sequence, displayed in the last column; in some cases they form runs of amino-acids (A–B), while in other cases they form large repetitive motifs of various purities (C–F). See Methods for more details.

### Compositional order (CO) of human proteins

We analyze the Swiss-Prot human proteome (N = 20248) in detail, employing our new measures. The human proteome is composed of CO proteins (N_CO_ = 5511, 27.2%) and NO proteins (N_NO_ = 14747); the latter do not contain any FT. Identifying FT occurrences on proteins allows for capturing a large repertoire of order patterns of peptide repeats of different levels of purity. Two outstanding examples of human proteins in Swiss-Prot records are: 1) the pure glutamine run in ATX8, a protein which consists of one M followed by 79 Q. Notably its DNA consists of an uncorrupted chain of 79 CAG repeats [36]. 2) 40 exact repeats of a peptide of length 20, VTSVPVTRPALGSTTPPAHD, on the protein MUC1. The variability in the number and purity of these repeats may differ among individuals [37], and plays an important role in cancer [38]. In Table 1 we present other types of order patterns caught by the FT analysis. One finds single amino-acid short runs of several amino-acid types, which may be distributed on various locations in a protein (Table 1 examples A–B), as well as repetitive motifs of various length and purity (Table 1, examples C–F), as found in many zinc-fingers (ZF), collagens and keratins. For high RP the repetitive motif is quite obvious (Table 1, examples C and F), while for moderate RP the underlying motif may be less obvious and the MFI may indicate its origin (Table 1, D and E are examples of motifs that seem to have undergone mutations). A full list of human CO proteins and their relevant CO measures, as well as other sequence information, is provided in Table S1.

**Figure 1:**
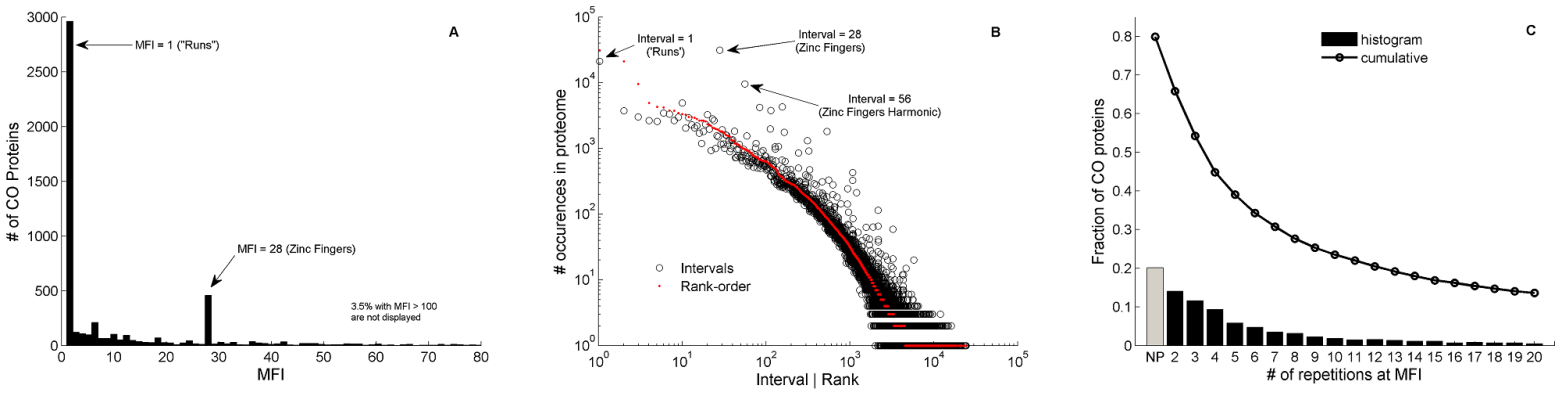
Analysis of Swiss-Prot human proteome. Analysis of Swiss-Prot human proteome (n = 20248) containing 5511 CO proteins. A) Histogram of the most frequent intervals, MFI, demonstrates the significant periodic structures originating in ‘runs’ of homo-peptides (MFI = 1) and zinc-fingers (MFI = 28). B) The frequency of intervals of all FTs in all proteins (black circles). The outstanding symbols are mostly due to Zinc-finger proteins which form repetitive sections of 28 amino-acids. Multiplicities at intervals 56, 84 amino-acid are also evident due to mutation acting on these sections. The superimposed red dots display the data in a rank-ordered manner (i.e. the *x*-axis takes on the role of rank rather than value of interval). C) The number of periodic proteins as defined by the number of FT occurrences at MFI. The bars indicate the fraction of CO proteins with exactly 2–20 (*x*-axis) occurrences at MFI. 20% of CO proteins are non-periodic (NP). Circles represent the cumulative fraction of proteins with number of repetitions at MFI above the value indicated by the *x*-axis. Thus, for a minimum of 4 repeats at MFI (i.e., *x = 3*), there are above 50% CO proteins with periodic structures.

The distribution of MFIs (figure 1a) as well the distribution of all intervals in the entire CO set (figure 1b), exhibit two leading periodic features in the proteome. One is that of MFI = 1 denoting prevalence of amino-acid runs, and the other is MFI = 28 which is characteristic of many ZF proteins. The interval distribution further displays higher harmonics of 56 and 84 on ZF proteins, which can be accounted for by mutation effects on amino acid sections with periodicity 28. Interestingly, the ranked-ordered interval distribution (figure 1b) displays behavior close to that of the well-known Zipf Law, a hallmark of linguistic elements (see Discussion). A periodic structure can be defined by requiring a minimal number of interval recurrences at MFI (figure 1c). In human, about 50% of CO proteins can be characterized as periodic with at least 4 interval repetitions, on which we find on average 6 DFTs and 30 recurrences at MFI.

The number of ZF proteins is quite prominent in the CO set (Table 2). It is of the order of 18%, doubling its relative weight compared to the total human proteome. Similar doubling is observed for collagen and keratin. The latter have substantial average values of RC, pointing to high relative coverage of FTs on their sequences, while ZF have high relative periodicity, RP. In contrast to all these examples, proteins annotated as disease-correlated, are not significantly enriched within the CO set. This would seem to run against the common understanding that disease related proteins have high compositional bias. The resolution is explained in the next section.

**Table 2:** Examples of compositional order and functional enrichment.

Function	within the proteome	within CO proteins	Mean RP	Mean RC
Disease	2755 (13.6%)	903 (16.4%)	0.3	0.1
Zinc Fingers	1799 (8.9%)	977 (17.7%)	**0.43**	0.17
Collagen	166 (0.8%)	87 (1.6%)	0.21	**0.25**
Keratin	162 (0.8%)	100 (1.8%)	0.27	**0.39**

Examples of selected functional groups with high CO in human. Based on Swiss-Prot records, the portions of each functional group in the entire proteome and within the CO set (i.e., proteins containing FTs) are given in numbers and percentages. Last columns indicate the average RC and RP, which should be compared with the overall mean values of 0.1 (RC) and 0.35 (RP) in the CO set (n = 5511).

### Functional enrichment and annotation dependencies on measures of compositional order on species chosen from different kingdoms throughout the tree of life

Our principal measures of CO regularity, RC, and periodicity, RP, can be used to sort out functions, cell-localizations and other annotations that are enriched with CO (see Methods). We carry out such analysis on three species: Human, *A. Thaliana*, and *S. Cerevisiae*, which may be viewed as representatives of three major taxonomic groups of eukaryotes: Animalia, Plantae and Fungi. Their proteomes in the Swiss-Prot data-base contain 20248, 11304, and 5875 proteins respectively. In addition, we have analyzed all the 187740 bacterial enzymes in Swiss-Prot.

#### Human

**Figure 2:**
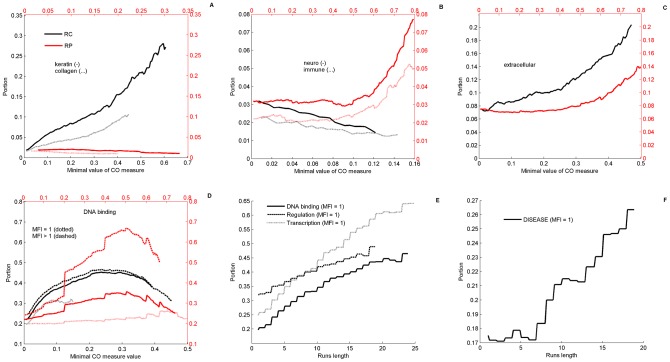
Repertoire of functional enrichments in human proteome. Repertoire of enrichment dependencies of GO (gene ontology) terms on the order measures of regularity, RC (black), and periodicity, RP (red). Portions of proteins belonging to a functional group are estimated based on text search in GO terms (see methods) and plotted in double axes against increasing thresholds of RC (lower x-axis) and RP (upper x-axis). A) The portions of some terms that are enriched with increasing threshold of RC but not of RP, like keratin (solid) and collagen (dotted). In this category one finds also filament and cell adhesion related proteins. B) GO terms that are enriched only for increased RP threshold but not RC, as neuronal related proteins (solid) and immune system proteins (dotted). These include also synaptic function and cell response genes. C) Other terms like extracellular region are enriched with increasing the threshold of both RC and RP. D) Some functionalities show more complicated non-monotonic “bump” behaviors. These include DNA-binding, regulation and transcription. As an example, DNA binding are further analyzed showing functional dependencies on RP and RC of both repetitive sections (MFI>1) and runs (MFI = 1). E) MFI = 1 proteins exhibit stable enrichment pattern as function of the threshold on the sum of repetitions at MFI = 1 (i.e., the effective coverage of all amino-acids “runs”). F) Disease related proteins are enriched with increasing length of runs. In each plot, the portion of the corresponding GO-term in the entire CO Swiss-Prot reviewed proteome is the value displayed at 0.

Analysis of human CO proteins using the GOrilla GO (gene ontology) tool [39], shows, consistently with previous studies, that human CO proteins exhibit numerous and highly significant functional enrichments (Text S2). Notably, these include regulation, transcription, binding, and various developmental and metabolic biosynthetic processes (Hypergeometric *P*-values<10^−8^, FDR corrected). In figure 2, we further demonstrate, using text search in GO annotations (see Methods), that CO human proteins exhibit a repertoire of enrichment dependencies on CO measures. Some functions depend on RC (figure 2A): keratin (*P*-value<10^−12^) and collagen (*P*-value<10^−12^). In this category one finds also filament and cell adhesion related proteins. Other functions depend on the RP (figure 2B), such as neuronal (*P*-value<10^−6^) and immune system related proteins (*P*-value<10^−5^) and other response proteins. Some depend on both RC and RP (figure 2C), e.g., extracellular proteins (*P*-value<10^−10^ for RC, and <10^−6^ for RP). There also exist proteins that have a non-monotonic behavior (figure 2D). In this last category we note some outstanding terms that have been previously discussed in the literature in the context of compositional bias: DNA-binding, regulation and transcription. To better understand the association of these annotations with CO measures we further explore how they depend on MFI. We analyzed separately two sub-groups of these proteins (figure 2D), those with significant amino-acid runs (MFI = 1) and its complement (MFI>1). The subgroup MFI>1 shows the highest enrichment with RP, indicating that, repetitive sections other than runs play important roles in the evolution of these functions. The subgroup MFI = 1 further displays a clear monotonic behavior with respect to the length of runs (figure 2E). The larger the coverage of amino-acid runs, the larger is the portion of proteins associated with these three annotations (*P*-values<10^−12^, 10^−7^, 10^−12^ for DNA binding, regulation and transcription, respectively). Disease proteins do not show any clear behavior with respect to RC or RP, however, a monotonic enrichment was found with respect to an increase in length of runs (figure 2F) with moderate *P*-value<10^−4^. Last, we note that there are several GO terms for which one finds monotonic decrease of the portion of related proteins for elevated thresholds of both RP and RC. These include ATP, cell-cycle, signal transduction, proliferation and growth. All these basic functions of living systems presumably evolve without relying on CO-structures, implying strong accumulation of mutations or purifying selection. We will point out below that increasing CO is correlated with organism complexity; hence, the fact that the most basic mechanisms do not require an increased CO is consistent with our analysis.

#### Arabidopsis thaliana

**Figure 3:**
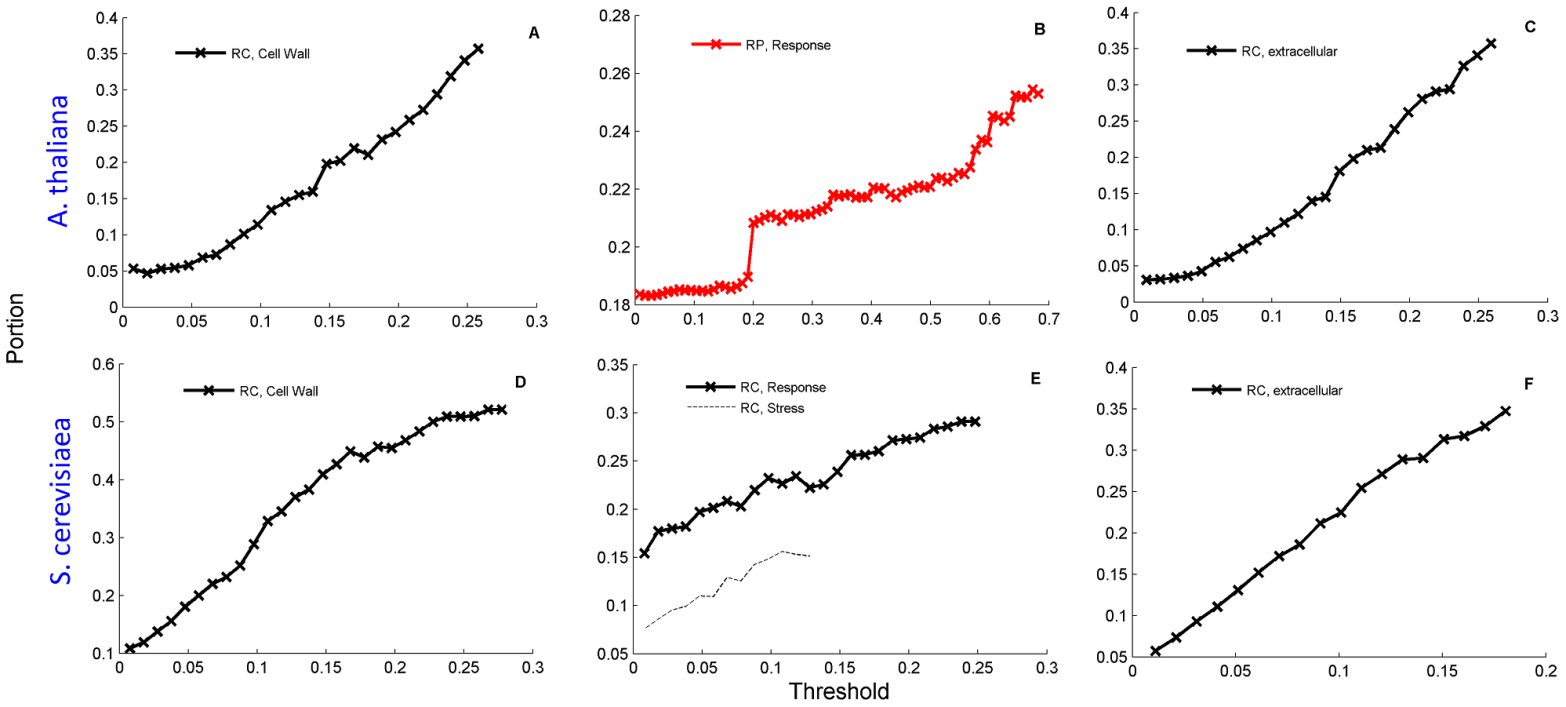
Functional enrichment in *A. Thaliana* and *S. cerevisiae*. Similarly to figure 2, functional enrichment in *A. Thaliana* (A–C) and *S. cerevisiae* (D–F) are shown with respect to RC (black) or RP (red). Portions of cell wall genes (A, D) and extracellular related genes (C, F) are enriched with increasing the threshold of RC, while portions of response related genes (B, E) are enriched with RP in *A. thaliana* but RC in yeast.

Some plant genomes contain large repetitive sections whose protective roles in stressful conditions have been suggested previously [41], [42]. In *A. thaliana* we find 1786 CO proteins comprising 15.8% of the proteome. Figure 3 (upper panel) describes the enrichment patterns found in **cell wall** (figure 3A, *P*-value<10^−11^), **response** (figure 3B, *P*-value<10^−4^) and **extracellular** related proteins (figure 3C, *P*-value<10^−12^). While cell wall and extracellular region related proteins were enriched for elevated thresholds of RC, the response related proteins are enriched with respect to RP. Notably, many of the high-RP proteins, beyond the scale of figure 3B (RP>0.7), are those involved with response to heat (HFA1A), cold (CAHC), cadmium and zinc ions (WAKLD), cytokinin stimulus (WOX9) oxidative stress (GPX6), salt stress (ULP1D, SRK2H), abscisic acid stimulus (ATE1, DNJ16), light stimulus (Y3210, GAT22), and defense response to bacterium or biotic stimulus (ATL55, EIL1, MLO9).

#### Saccharomyces cerevisiae

Main GO terms enriched in CO proteins (N_CO_ = 996, 17%) are similar to the ones found in *A. thaliana* as shown in figure 3 (lower panel), i.e., **cell wall** (*P*-value<10^−12^), **response** (*P*-value<10^−3^) and specifically **stress** related genes (*P*-value<10^−4^) and **extracellular region** genes (*P*-value<10^−12^). Also nuclear pore proteins are enriched, although their overall number is low. Furthermore, in both *A. thaliana* and *S. cerevisiae*, annotations of DNA-binding, regulation and transcription display similar enrichment patterns to those observed in human for the subgroup of proteins that contain significant runs, MFI = 1 (figure S10). Noted differences between *A. thaliana* and *S. cerevisiae* are in response related genes. While in *A. thaliana* enrichment is with respect to RP (figure 3B) in *S. cerevisiae* it is with respect to RC (figure 3E). This looks similar to the disappearance of RP-enrichment of extracellular proteins in *A. thaliana vs* human. We will argue below that it is consistent to assume that major CO generation occurs at macroevolutionary steps. Since the macroevolutionary birth of the *S. cerevisiae* lineage predates that of *A. thaliana* which clearly predates the several macroevolutionary steps in the human lineage, it is consistent to assume that RP dependencies of the former species have washed out during the long periods of microevolution, due to accumulating mutations.

#### Bacterial enzymes

Since the number of CO proteins in a bacterial proteome is quite small, of the order of few tens, functional enrichment in a single bacterial species is usually not conclusive. Therefore, we analyzed the ensemble of all the reviewed bacterial enzymes in Swiss-Prot (n = 187740), which contains 6240 CO enzymes (3.3%). Enrichment levels display dominance of cell wall and response proteins (figure S10 C), consistently with previous observations [43], [44].

Furthermore, our methodology allows us to pick up extreme examples of CO in bacterial enzymes, namely sections of protein sequences having high RC, which further elucidate how CO sections may accumulate throughout evolution. Two outstanding examples are Lysostaphin enzymes in two different species that belong to the same genus, *Staphylococcus simulans* and *S. staphylolyticus*. In both cases these enzymes contain a long repetitive section of 15*x*13 amino acid approximate tandem repeats of AEVETSKAPVENT. This long section serves as a pro-peptide chain which is uniquely associated with these two enzymes. Another example is XYNA_RUMFL, belonging to *Ruminococcus flavefacien*. This enzyme has a long midsection which is highly enriched with Asparagine (N) and Glutamine (Q), captured by the FT QNN, and has no homolog elsewhere. As a last example we point to SYE_TRIEI which has a standard Glutamyl-tRNA synthetase domain on its first 480 amino-acids, followed by a highly repetitive domain for the next 400 amino-acids (containing the FTs ATD, ATT, PVA, TAT, TPV, VAT). This long chain has no homolog anywhere in the known protein world. All these examples serve as a show-case for CO sections of proteins that did not spread to other organisms; however they survived within these organisms, presumably because they don't have deleterious effects. As such they allow us a glimpse into how very rich CO can emerge. In analogy with paleontological evidence, we may assume that many other CO variations have been tried by nature, and have been either discarded or reshaped into useful novel genes.

### Comparative proteomic analysis of compositionally ordered sets of Human and Mouse

In this section we perform comparative proteomic analysis of human and mouse, based on Swiss-Prot data. The relationship between the proteins of these two species is summarized in Table 3. The 20248 human proteins and the 16513 mouse proteins are sorted out according to whether they are CO or not (NO) and, according to whether they are orthologs (V) of each other or not (X) as indicated by Swiss-Prot annotations. Some of the 8 subsets have interesting features that we describe below.

**Table 3:** Human and Mouse proteomes analysis.

Human	Mouse	Orthology	# of proteins	% of Human proteome	% of Mouse proteome
CO	CO	V	3312	16.4%	20%
CO	NO	V	831	4.1%	5%
NO	CO	V	626	3.1%	3.8%
NO	NO	V	10557	52.1%	63.9%
CO	—	X	1368	6.8%	—
NO	—	X	3554	17.6%	—
—	CO	X	125	—	0.8%
—	NO	X	1062	—	6.4%

Human and mouse proteomes were decomposed into compositionally ordered (CO) and non-ordered (NO) subsets as well as into Orthologous (V) and non-orthologous (X) proteins.

#### Analysis of the CO orthologous sets

**Figure 4:**
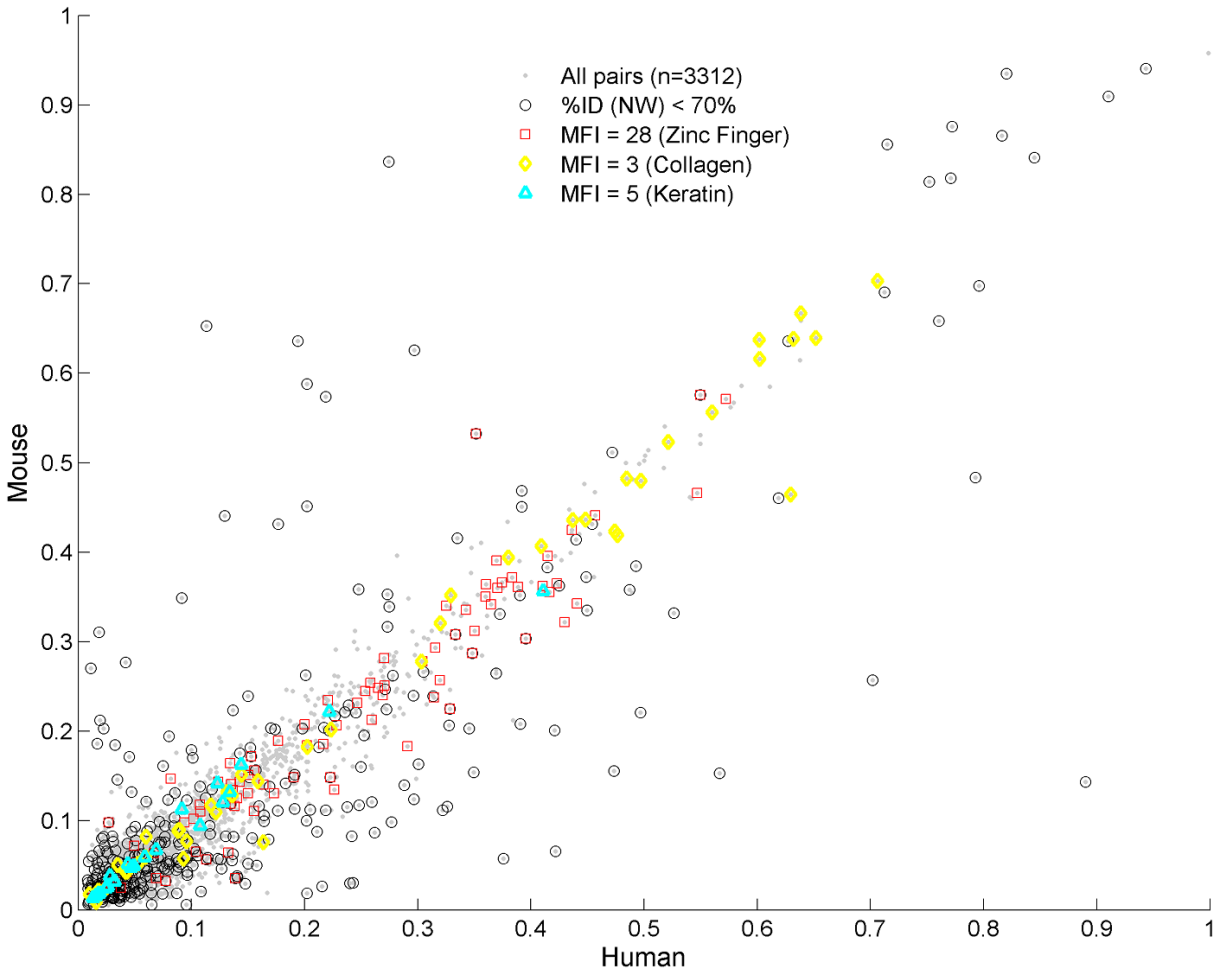
Comparison of CO orthologs in human and mouse. Comparison of CO orthologs in human and mouse according to their RC values. Each point corresponds to a pair of such proteins (n = 3312). Low homologies are marked by circles. Usually, their CO sections are comparable, however revealing higher harmonics in the mouse (Text S1 - section 7, figure S11). Off-diagonal pairs always display low homologies. In the upper-left diagonal CO sections of human and mouse resemble each other, having high similarity of FTs and MFI, despite the low RC in human. In the lower-right diagonal mouse CO sections do not resemble human CO sections, except for few exceptions (see text). High homology is obtained for protein pairs with similar MFIs, such as zinc finger (MFI = 28), collagen (MFI = 3) and keratin (MFI = 5) proteins, and lie along the diagonal.

Comparison of RC values in mouse and human for the 3312 orthologous CO proteins is shown in figure 4. Along the diagonal of figure 4 we find high similarity of sequences, FTs and their periodic properties. High RC values are associated with some well-known protein families, Zinc fingers (MFI = 28, 56), Collagen (MFI = 3, 6), Keratin (MFI = 5, 10). Because the lower harmonics are more prevalent, the existence of the higher harmonics suggests the effect of mutations, while the simultaneous conservation of function and high CO in both species suggests that selection played a role in maintaining them.

Pairs that deviate from the diagonal, and have high RC in one species but relatively low RC in the other, have also low sequence identity (<70%), as measured by Needleman–Wunsch (NW) global alignment. These low sequence similarity proteins comprise 11.5% (380/3312) of the studied set. Interestingly, we find that protein pairs in the upper off-diagonal (i.e., low RC in human and high RC in mouse, including SPR1B, MUC4, ZN239, K1C9, F186A, RPNT, SBSN, ZAN) display similarity between their FTs and MFIs in both species. For example, the protein K1C9 has MFI = 8 in both species and similar prevalent FTs (SGG and GSG). In contrast, the lower off-diagonal pairs (high RC in human and low RC in mouse, including CQ097, FILA, MUC2, MUC20, PGCA, PHGR1, SPRR3, INVO) have low similarity of the CO sections , i.e. usually their FTs and MFIs are different. Two exceptions are PRG4, with MFI = 8 in both species (FT = PTT), and TXND2 with MFI = 15 (FT = PKS).

Both types of observation are consistent with the fact that the mouse lineage exhibits higher substitution rates [45], [46]; hence some CO structures that existed on the common ancestor may more readily wash out in the mouse lineage if they are not needed for functional purposes. Few examples provided in Text S1 (section 7, figure S11) present evidence that the repetitive sections are subjected to mutational forces that lead to an increase in the intervals in mouse and to the creation of harmonics. In Text S1 (section 7, figure S12) we have further quantified the discrepancy in mouse intervals distribution compared to their human orthologs in all proteins that contain periodic structures (MFI>1), showing that high harmonics are more prevalent in mouse by a factor larger than 2.

Because orthologous proteins in human and mouse originate from a common ancestor, it is of particular interest to study the sets in which the CO property has been lost or gained in one of the species (Table 4, sets H2 and M2). Their average RC is at bare minimum, whereas their average RP is high, compared to the orthologous CO sets. This may indicate that in these cases while RC decreases (low FT coverage) functional evolutionary constraints retain periodicity which leads to the high RP. However, we did not find any significant functional enrichment is these subgroups. Therefore, it is possible that high RP based on only few FTs, reflecting low RC, may simply indicate the degeneration of CO, a phenomenon that we have encountered in the off-diagonal proteins in figure 4. Alternatively, the high RP and low RC in these sets may reflect the generation of new CO sections in the respective species with no evident functional purpose.

**Table 4:** Human and Mouse CO set – Enrichment by RC and RP.

species	CO Set name	Orthology	# of CO proteins	RP(*P*-value)	RC(*P*-value)
Human (n = 5511)	**H1**	V (CO in mouse)	3312	0.33	0.09
	**H2**	V (NO in mouse)	831	**0.4**(2.1×10^−35^)	**0.03**(6.02×10^−68^)
	**H3**	X	1368	**0.36**(2.25×10^−11^)	**0.19**(7.56×10^−62^)
Mouse (n = 4063)	**M1**	V (CO in human)	3312	0.33	0.08
	**M2**	V (NO in human)	626	**0.44**(1.16×10^−34^)	**0.04** (1.18×10^−51^)
	**M3**	X	125	0.34	**0.16**(9.8×10^−5^)

The CO sets of Human (H) and mouse (M) are decomposed into CO orthologous proteins (V) that appear in both species (H1, M1), to orthologous proteins that are CO in one species but not (NO) in the other (H2, M2) and to non-orthologous genes (X) belonging to the CO sets (H3, M3). The values of RC and RP are shown for each subgroup in each species. *P*-values correspond to Kolmogorov-Smirnov 2 sample test of each group in a species compared with the subgroup 1 of the same species (i.e., H1 and M1 respectively).

#### Analysis of CO non-orthologous novel protein sets

Another interesting case is that of CO sets which are novel (Table 4, sets H3 and M3), i.e. have been created after the two lineages have separated from each other. The latter are particularly abundant in human: whereas human has 1368 non-orthologous CO proteins (set H3), mouse has only 125 such proteins (set M3). This discrepancy by an order of magnitude is quite astounding, indicating that novel CO proteins have accumulated to a larger extent on the human lineage since its departure from the mouse lineage. Comparable novelty is observed also for all non-orthologous proteins (both CO and NO sets): 4992 in human vs 1187 in mouse. Sets H3 and M3 are highly enriched in RP and RC values in comparison to the CO orthologous sets (Table 4, sets H1, M1, respectively). Thus, CO of the novel proteins is higher than that of the older ones. A large fraction of the 1368 novel CO proteins in human are ZF, containing 433 out of a total of 977 ZF in the overall CO set of human (n = 5511). When ranking the proteins by their RC values, a GOrilla analysis (Text S3) provides *P*-values<10^−42^ for functions carried by ZF proteins, and when ranked by RP the analogous *P*-value reduces to 10^−80^. Other outstanding protein families are keratin-associated proteins (61 novel, out of 94 in the CO set) and protocadherins (44 novel, out of 55 in the CO set).

In view of the large disparity between the numbers in H3 and M3 we have also extracted the number of non-orthologous proteins in human and mouse using various other databases and methods, and re-assessed the ratio between human and mouse novel CO proteins (Text S1 – section 13 and Table S5). The ratios we obtain are in the range of 2 to 5. Thus, although the discrepancy is less pronounced than in the Swiss-Prot set, it is still large and significant. Consistently, all sets show that significantly many of the human novel CO proteins are Zinc Fingers.

### Compositional Order vocabulary serves as a signature of macroevolution

In order to study effects of CO for a wide range of species, we have extracted from the NCBI-RefSeq data-base well annotated proteomes, listed in Table 5, of 39 eukaryotes (including 7 protista), 36 bacteria and 19 archaea, distributed across the tree-of-life [47]. In this table, we ordered the species according to the kingdoms Animalia, Plantae, Fungi, Bacteria and Archaea. The ordering of the eukaryotes follows the tree-of-life, which is also a reasonable ordering of organism complexity. We explore the FT-vocabulary, a measure of proteomic CO richness, which is defined by the total number of Different Frequent Triplets (DFT). This is the count of FT types rather than the number of FT occurrences on the proteome. Proteomic DFT counts displayed in Table 5 are insensitive to redundancy, because two identical proteins in a proteome contribute the same FTs.

**Table 5:** List of 94 species.

Index	DFT	Species	Taxonomy
1	5076	Human (Homo Sapiens)	Animal (V)
2	4333	Chimpanzee(pan troglodytes)	Animal (V)
3	4873	Mouse (Mus musculus)	Animal (V)
4	4815	Rat (Rattus Norvegicus)	Animal (V)
5	4559	Dog (Canis lupus familiaris)	Animal (V)
6	2901	Platypus (Ornithorhynchus Anatinus)	Animal (V)
7	4419	Chicken (Gallus gallus)	Animal (V)
8	3216	Zebra Finch (Taeniopygia guttata)	Animal (V)
9	3989	Lizard (Anolis Carolinensis)	Animal (V)
10	5299	Zebrafish (Danio rerio)	Animal (V)
11	4019	Sea Squirt (Ciona intestinalis)	Animal (IV)
12	4146	Fruit Fly (Drosophila melanogaster)	Animal (IV)
13	3518	Mosquito (Anopheles Gambiae)	Animal (IV)
14	3225	Bee (Apis Mellifera)	Animal (IV)
15	3722	Nematode (C. elegans)	Animal (IV)
16	2630	Nematode (Brugia Malayi)	Animal (IV)
17	2262	Arabidopsis thaliana	Plant
18	2785	Medicago truncatula	Plant
19	2094	Populus trichocarpa	Plant
20	2286	Physcomitrella patens	Plant
21	2770	Chlamydomonas reinhardtii	Plant
22	1846	Rice (Oryza sativa Japonica)	Plant
23	1993	Sorghum bicolor	Plant
24	1037	Maize (Zea may)	Plant
25	1838	Nectria haematococca	Fungi
26	1858	Botryotinia fuckeliana B05.10	Fungi
27	1411	Aspergillus niger CBS 513.88	Fungi
28	936	Ajellomyces_capsulatus NAm1	Fungi
29	1439	candida albicans SC5314	Fungi
30	1112	Candida albicans WO1	Fungi
31	1077	S. Cerevisiae	Fungi
32	1033	S. Pombe	Fungi
33	2990	Dictyostelium Discoideum	Protista
34	1380	Entamoeba Histolytica	Protista
35	2319	Leishmania Major	Protista
36	2740	Phytophthora Infestans	Protista
37	1230	Plasmodium Chabaudi	Protista
38	3404	Plasmodium Vivax	Protista
39	2129	Thalassiosira Pseudonana	Protista
40	823	Staphylococcus aureus MRSA252	Bacteria, Firmicutes
41	644	Bacillus anthracis AMES	Bacteria, Firmicutes
42	432	Bacillus subtilis str168	Bacteria, Firmicutes (T)
43	413	Symbiobacterium thermophilum	Bacteria, Firmicutes
44	344	Mycoplasma penetrans HF-2	Bacteria, Firmicutes
45	332	Alicyclobacillus acidocaldarius	Bacteria, Firmicutes (T)
46	210	Lactococcus lactis cremoris MG1363	Bacteria, Firmicutes
47	154	Caldocellum saccharolyticum	Bacteria, Firmicutes (T)
48	137	Streptococcus agalactiae NEM316	Bacteria, Firmicutes
49	1007	streptomyces coelicolor A3(2)	Bacteria, Actinobacteria
50	670	Mycobacterium tuberculosis CDC1551	Bacteria, Actinobacteria
51	454	Arthrobacter aurescens TC1	Bacteria, Actinobacteria
52	274	Corynebacterium glutamicum ATCC13032	Bacteria, Actinobacteria
53	2452	Chlorobium chlorochromatii CaD3	Bacteria, Chlorobi
54	202	Bacteroides thetaiotaomicron VPI-5482	Bacteria, Bacteriodes
55	179	Bacteriodes fragilis YCH46	Bacteria, Bacteriodes
56	126	Bacteroides caccae ATCC 43185	Bacteria, Bacteriodes
57	83	Chlamydophila pneumoniae AR39	Bacteria, Chlamydiae
58	90	Chlamydia trachomatis A2497	Bacteria, Chlamydiae
59	330	Fusobacterium nucleatum ATCC 25586	Bacteria, Fusobacteria
60	77	Thermotoga maritima	Bacteria, Thermotogae (T)
61	45	Thermotoga lettingae TMO	Bacteria, Thermotogae (T)
62	82	Aquifex aeolicus	Bacteria, Aquificae (T)
63	267	Thermomicrobium roseum	Bacteria, Chloroflexi (T)
64	261	Thermus thermophilus	Bacteria, Deinococcus-Thermus (T)
65	1627	Nostoc punctiforme PCC 73102	Bacteria, Cyanobacteria, Nostocaceae
66	630	Gloeobacter violaceus PCC 7421	Bacteria, Cyanobacteria, Gloeobacteraceae
67	402	Prochlorococcus marinus MIT 9303	Bacteria, Cyanobacteria, Synechococcaceae
68	1167	Geobacter uraniireducens Rf4	Bacteria, Protobacteria, Delta
69	543	Yersinia pestis Antiqua	Bacteria, Protobacteria, Gamma
70	482	Shewanella baltica OS155	Bacteria, Protobacteria, Gamma
71	432	Bordetella pertussis Tohama I	Bacteria, Protobacteria, Beta
72	403	Caulobacter crescentus CB15	Bacteria, Protobacteria, Alpha
73	268	Brucella suis 1330	Bacteria, Protobacteria, Alpha
74	249	Ecoli K12 MG1655	Bacteria, Protobacteria, Gamma
75	100	Helicobacter cinaedi CCUG 18818	Bacteria, Protobacteria, Epsilo
76	1665	Cenarchaeum symbiosum A	Archaea
77	883	Nitrosopumilus maritimus SCM1	Archaea
78	582	Methanosphaera stadtmanae	Archaea
79	522	Haloquadratum walsbyi	Archaea
80	495	Methanospirillum hungatei	Archaea
81	285	Natronomonas Pharaonis	Archaea
82	193	Halobacterium salinarum R1	Archaea
83	182	Methanopyrus kandleri	Archaea (T)
84	173	Pyrobaculum aerophilum	Archaea (T)
85	141	Aeropyrum pernix K1	Archaea (T)
86	141	Methanococcus maripaludis	Archaea
87	130	Metallosphaera sedula	Archaea (T)
88	124	Sulfolobus solfataricus	Archaea (T)
89	114	Methanothermobacter thermautotrophicus	Archaea (T)
90	99	Archaeoglobus fulgidus DSM4304	Archaea (T)
91	98	Picrophilus torridus DSM9790	Archaea (T)
92	73	Pyrococcus furiosus	Archaea (T)
93	73	Pyrococcus abyssi GE5	Archaea (T)
94	48	Nanoarchaeum equitans Kin4-M	Archaea (T)

List of the 94 species distributed across the tree-of-life studied in the large-scale analysis and their taxonomic identities, Eukaryotes (1–39) and Prokaryotes (49–94). The ordering of species is according to the tree-of life [47]. Within Eukaryotes, kingdoms are first ordered from Animalia to Plantae (P) to Fungi (F). Animalia are classified as vertebrates (V), and invertebrates (IV). Within each kingdom ordering is according the phylogenetic distance from the first species, i.e. Human within Animalia, *A. thaliana* within Plantae and *Nectria* within Fungi. Protista (PRT) are added at the end with no phylogenetic analysis. Bacteria are also ordered according to the Phylum as presented in [47], where within each Phylum the ordering is according to DFT counts. Archaea are ordered by DFT counts. Mesophiles (M) and Thremophiles (T) are indicated.

One may discern in Table 5 a general trend of decrease in DFT counts among eukaryotes with increasing evolutionary distance from human, excluding the 7 protista which are added at the end of the list of eukaryotes in arbitrary order because of uncertainties in their phylogenies. Bacteria are ordered by the phylogenetic distance between phyla from firmicutes to protobacteria, with decreasing DFT counts within each phylum. Archaea are ordered by DFT counts. DFT counts of prokaryotes are mostly in the hundreds, with few exceptions in the thousands; the latter usually occur because of few highly ordered long genes, unlike in eukaryotes (see Methods sensitivity analysis, and Text S1 - section 2).

**Figure 5:**
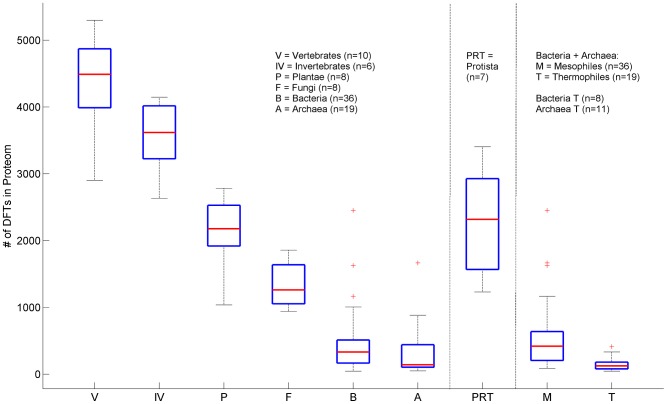
DFT Box-plot by Kingdom. Box plots of DFT counts across the tree-of-life. Each box delineates lower quartile, median and upper quartile values. Most extreme values (whiskers) are within 1.5 times the inter-quartile range from the ends of the box. Outliers are also displayed. Prokaryotes are displayed twice. First divided according to bacteria and archaea, and secondly as mesophiles and thermophiles. *P*-values according to non-parametric two-sample Kolmogorov-Smirnov test are 2.5×10^−2^ (V-IV), 6.5×10^−3^(IV-P), 9×10^−3^ (P-F), 1.7×10^−5^ (F-B), 2.3×10^−2^(B-A) and 1.4×10^−4^ (M-T). Protista species show large variability and cannot be distinguished from Plantae or Fungi. Abbreviations: Vertebrates (V), Invertebrates (IV), Plantae (P), Fungi (F), Protista (PRT) Bacteria (B) Archaea (A), Mesophiles (M), Thermophiles (T).

The data of Table 5 are grouped together into major taxonomic divisions in figure 5. The latter exhibit characteristic ranges of DFT counts that distinguish these divisions from one another, allowing for a meaningful and significant hierarchical order differentiating the successive kingdoms (Kolmogorov-Smirnov *P*-values≤10^−2^). The decrease of DFT may be also correlated with a decrease in the complexity of the organism. This correlation is not exact; however the trend is clear, yielding a decrease by factor of 5 from human to yeast. Protista are exceptional, with DFT counts overlapping with those of plants and fungi. This is consistent with the conventional view of protista as being a diverse grouping of organisms that may not be closely related via evolution. Among prokaryotes, we find an interesting systematic functional trend of DFT counts: thermophiles exhibit much lower numbers (few tens) than mesophiles, with differentiating *P*-value of 1.4×10^−4^. This is in agreement with the analysis of Pe'er et al. [33] which found bacterial thermophiles to be more closely related to archaea. It seems quite natural to expect that the low DFT counts of thermophiles are due to evolutionary pressure, since highly repetitive amino-acid sequences may be less stable under extreme temperatures. The few observed FTs may be important to induce favorable structural changes [48].

No hierarchical order of the kind displayed here can be achieved by measures such as the number of proteins, fraction of CO proteins, average protein lengths (Text S1 - section 3, figure S6) or other genomic characteristics [49]. It is interesting to note the species that seem to possess extreme DFT counts within their particular kingdoms. Fungi with the largest number of DFTs are plant pathogens (*Nectria haematococca*, *Botryotinia fuckeliana*). Bacteria and archaea with very large DFT counts live in aquatic or cold environments (*Chlorobium chlorochromatii*, *Cenarchaeum symbiosum*) or possess very complex functionalities (*Nostoc punctiforme*). This may support the view that ecological and environmental conditions, such as decrease in temperature, or inter-species hybridizations as in the case of amphidiploids in plants [50], had shaped DFT distributions in these species. In Text S1 (section 6) we present a reanalysis of the same data using a modified restrictive definition of FTs which further abolishes any length-dependent contribution (see Methods), resulting in figure S9. The same characteristics are obtained with slightly different *P*-values. We conclude that the proteomic DFT counts lead to a unique correlate of evolution, which is insensitive to the exact FT definition, providing a distinguishing hierarchical order-parameter.

**Figure 6:**
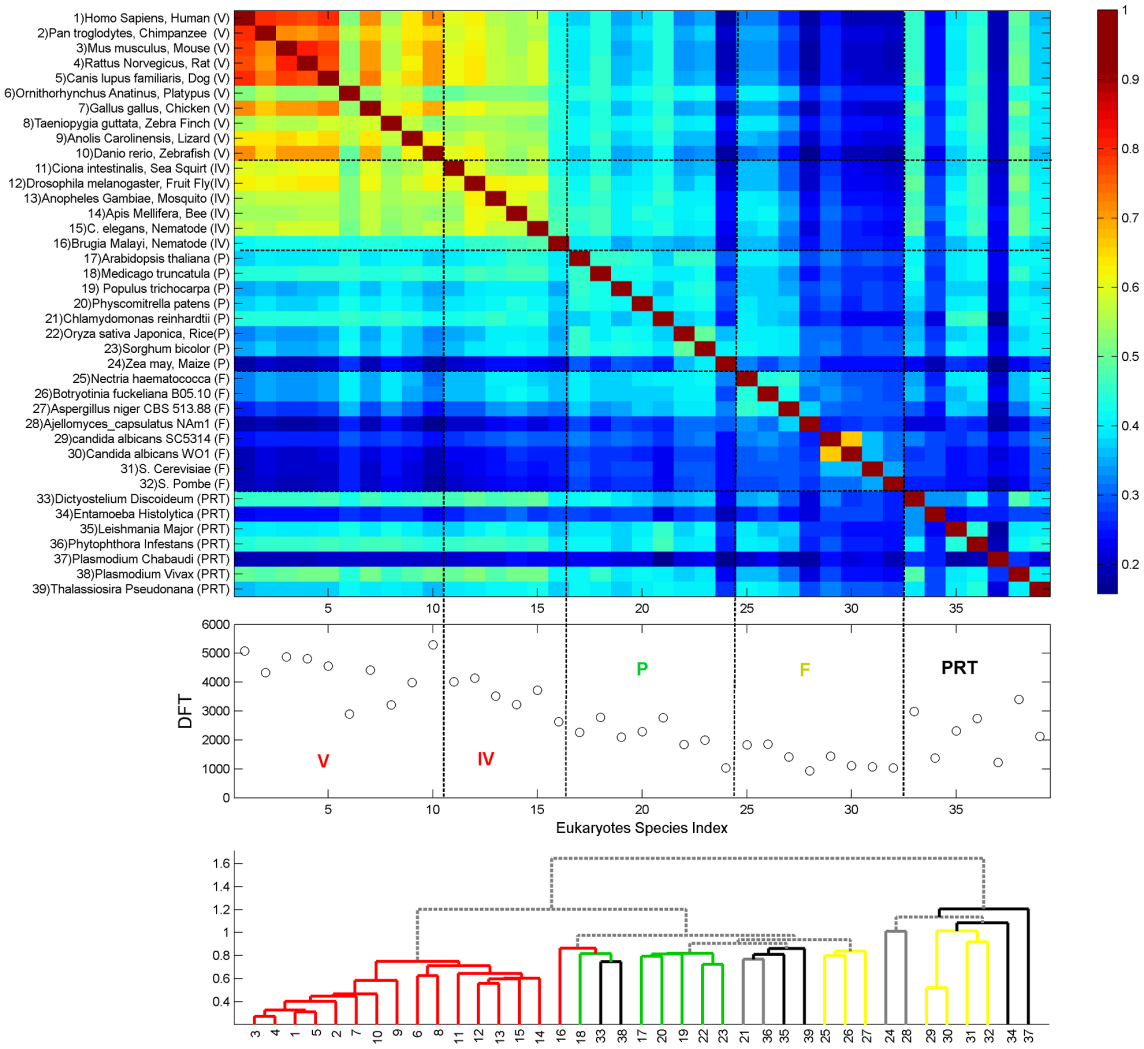
DFT enrichment in eukaryotes. DFT count and correlation *C_IJ_* of the 39 studied eukaryotes. Species are indexed and ordered as in table 5, according to the kingdoms Animalia, Plantae, Fungi and within each kingdom, according to their phylogenetic distance. The upper panel shows the heat-map of the correlation *C_IJ_*, the middle panel shows the DFT counts, and the lower panel shows the tree of hierarchical clustering based on Euclidian average distance of *C_IJ_*. Colors of the branches correspond to the taxonomic identity as indicated by the colored abbreviations in the middle panel. Abbreviations are the same as defined in figure 5. Solid gray branch corresponds to two proximate ends-leafs belonging to different taxonomic groups. Dashed gray branches link groups.

**Figure 7:**
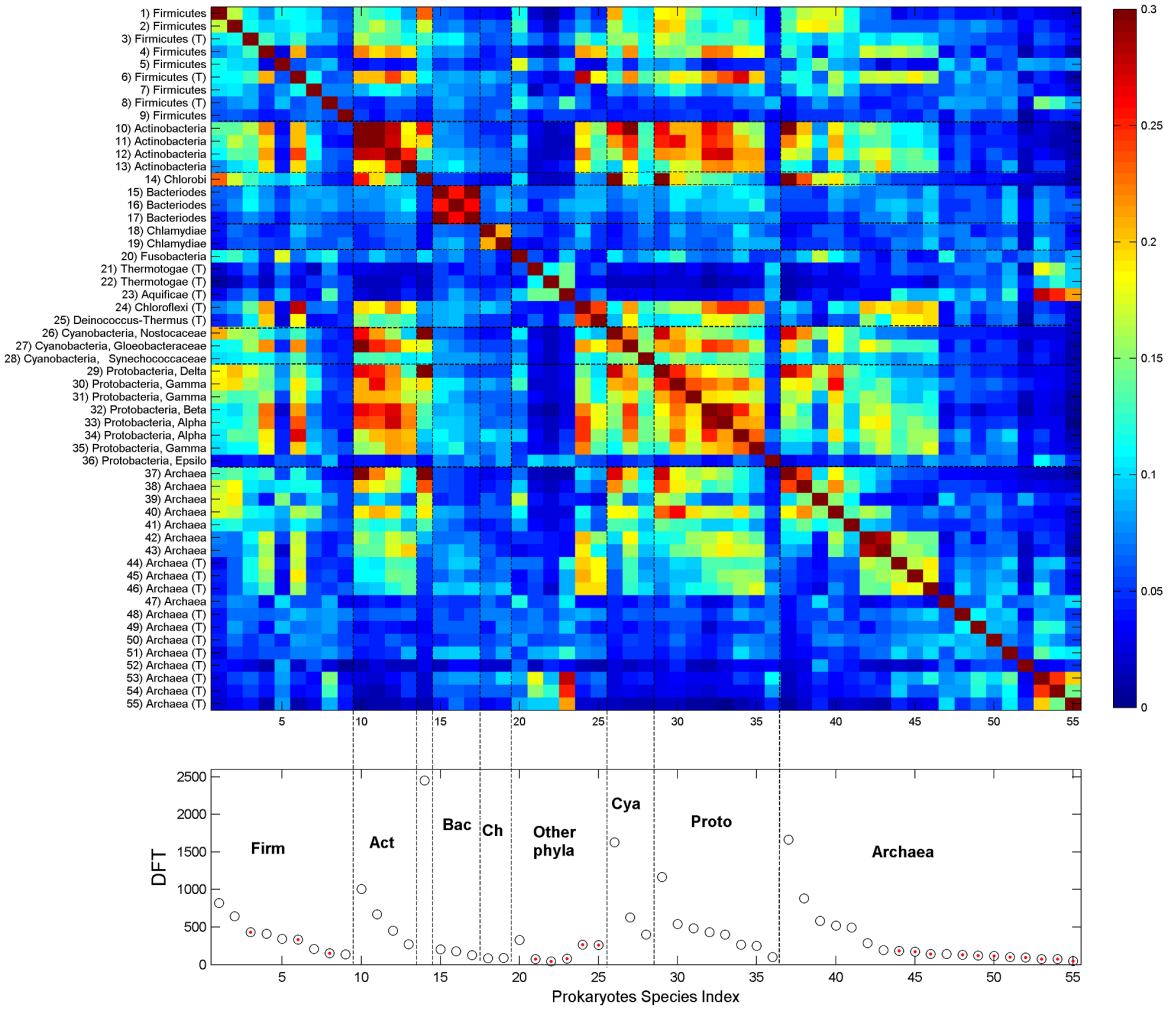
DFT enrichment in prokaryotes. DFT count and correlation *C_IJ_* of the 55 studied prokaryotes. Bacteria are grouped into phyla which are ordered according to their phylogenetic distance, from firmicutes to proteobacteria, and within each phylum species are ordered by DFT counts. Archaea are ordered by DFT counts. Upper panel displays the heatmap of *C_IJ_*, lower panel displays DFT counts (red points indicate thermophiles). Color scale is different from figure 6, in order to be able to trace trends which extend over several orders of magnitude. Abbreviations: Firmicutes (Firm); Actinobacteria (Act); Bacteriodes (Bac); Chlamydiae (Ch); Cyanobacteria (Cya), Protobacteria (Proto), Mesophiles (M), Thermophiles (T).

This observation motivated us to analyze the identities of the DFT contents of different proteomes. Defining *DFT_I_* to be the set of DFTs in proteome *I*, we look for the DFT-correlation between different proteomes *I* and *J*, defined by the Jaccard index: *C_IJ_ = (DFT_I_∩DFT_J_)/(DFT_I_ ∪DFT_J_)*. The results are displayed in figure 6, containing all eukaryotes, and figure 7, containing all prokaryotes. In eukaryotes, the divisions between the sets displayed in figure 5 are delineated also through the *C_IJ_*. Moreover, mammals (id 1–5) stick out among the vertebrates (id 1–10), having distinct DFT sets of their own. Invertebrates (id 11–16) have the largest correlation to vertebrates; and plants (id 17–24) show some correlation with animals, and less with fungi (id 25–32). In contrast, some protista species (id 33–39) show an unexpected correlation which gradually decreases from Animalia to Plantae to Fungi. We note the particular case of Zebra-Fish (id 10), whose DFT count supersedes that of human yet its correlation with mammals is not too high. Hierarchical clustering, using Euclidian average distance of *C_IJ_*, shows that the hierarchical tree largely overlaps the phylogentic tree. Animals, Plants and Fungi form separated clusters with only little misplacement (figure 6, bottom). Three Fungi that are plant pathogens, *Nectria haematococca*, *Botryotinia* and *Aspergillus niger* are grouped with Plants. Protista species are distributed across the tree.

In prokaryotes, bacteria (id 1–36) are ordered in phyla, with decreasing DFT counts within each phylum. Archaea (id 37–55) are ordered by DFT counts. The correlation among different bacterial phyla is relatively strong, except for bacterioidetes (id 15–17) and chlamydiae (id 18–19), which have weak similarity to other species. The thermophiles Thermotogae (id 21–22) and Aquificae (id 23) also have weak similarity to other species, but they have strong similarity to the last 3 archaea which are themophilic as well. The bacterium with outstanding DFT is *Chlorobium* due to a particularly long protein, the parallel beta-helix protein composed of 36800 amino acids. It has high correlation with some cyano- and proto- bacteria. Archaea mesophiles show significant correlation to other bacterial species. In contrast, some Archaea thermophiles have either distinct DFTs of their own, or they possess significant correlation with other thermophiles (either archaea or bacteria).

Another evident difference between the kingdoms is the identity of the most abundant FTs in the proteomes. They are presented in Table 6 for several selected species, highlighting those resulting in amino-acid runs. The latter are significantly more abundant in eukaryotes than in prokaryotes (figure S13).

**Table 6:** Predominant FTs in selected species.

	Human	Mouse	Fly	C. elegans	A. thaliana	S. cerevisiae	E. coli
1	**EEE**	**EEE**	**QQQ**	**SSS**	**SSS**	**SSS**	**LLL**
2	**SSS**	**SSS**	**SSS**	**PPP**	**EEE**	**QQQ**	LLA
3	**PPP**	**PPP**	**AAA**	**TTT**	**GGG**	**EEE**	**AAA**
4	**LLL**	**LLL**	**GGG**	**QQQ**	**PPP**	**NNN**	LAA
5	**AAA**	**AAA**	**PPP**	**GGG**	**QQQ**	**DDD**	ALL
6	CGK	**GGG**	**TTT**	**EEE**	**DDD**	SST	EAA
7	HTG	GEK	**NNN**	**AAA**	**LLL**	**TTT**	GRL
8	GEK	HTG	SGS	**KKK**	**AAA**	TSS	RLT
9	TGE	CGK	GSG	STS	**KKK**	STS	AAG
10	**GGG**	EKP	**EEE**	TSS	**NNN**	**LLL**	AAK
11	EKP	TGE	GSS	PPG	SSL	NSS	AEA
12	ECG	KPY	QQH	PGP	DLS	**KKK**	ALA
13	KPY	**QQQ**	SST	**DDD**	LLS	SSL	APA
14	**QQQ**	KAF	**HHH**	GPP	**TTT**	**PPP**	DRL
15	KAF	SSL	SSG	APG	SLL	SKK	LAE
16	GKA	**KKK**	**DDD**	GAP	SPS	LSS	LAL
17	IHT	GKA	SGG	APA	LDL	ATT	LLG
18	**KKK**	SPS	SAS	SST	LSG	NSN	**QQQ**
19	PGP	PGP	TSS	PAP	LSS	**AAA**	RYD
20	HQR	PSP	**LLL**	STT	SPP	SLS	TLT

List of predominant FTs in several species. FTs are ranked according to the number of CO proteins in which they are found. FTs containing a single amino-acid, which represent amino-acid runs on the protein's sequence, are highlighted. The latter are significantly more abundant in Eukaryotes (see, figure S13).

Last, we applied the same technique of DFT correlations (defined at protein levels) to human proteins. The analysis shows that it leads to classification of principal functional groups, notably various metabolic processes of macromolecule biosynthesis, response to unfolded proteins and numerous developmental, morphological and anatomical structure proteins (Text S1 - section 8, figure S14). A compendium of human protein information is presented in figure S15. There we sort all CO proteins according to the clustergram (figure S14) and present the distributions of DFT numbers in proteins, and protein numbers in which each FT occurs. We also zoom-in onto the 50 leading FTs exhibiting characteristics of co-occurrences.

### Universality

#### DFT distribution functions in proteomes

**Figure 8:**
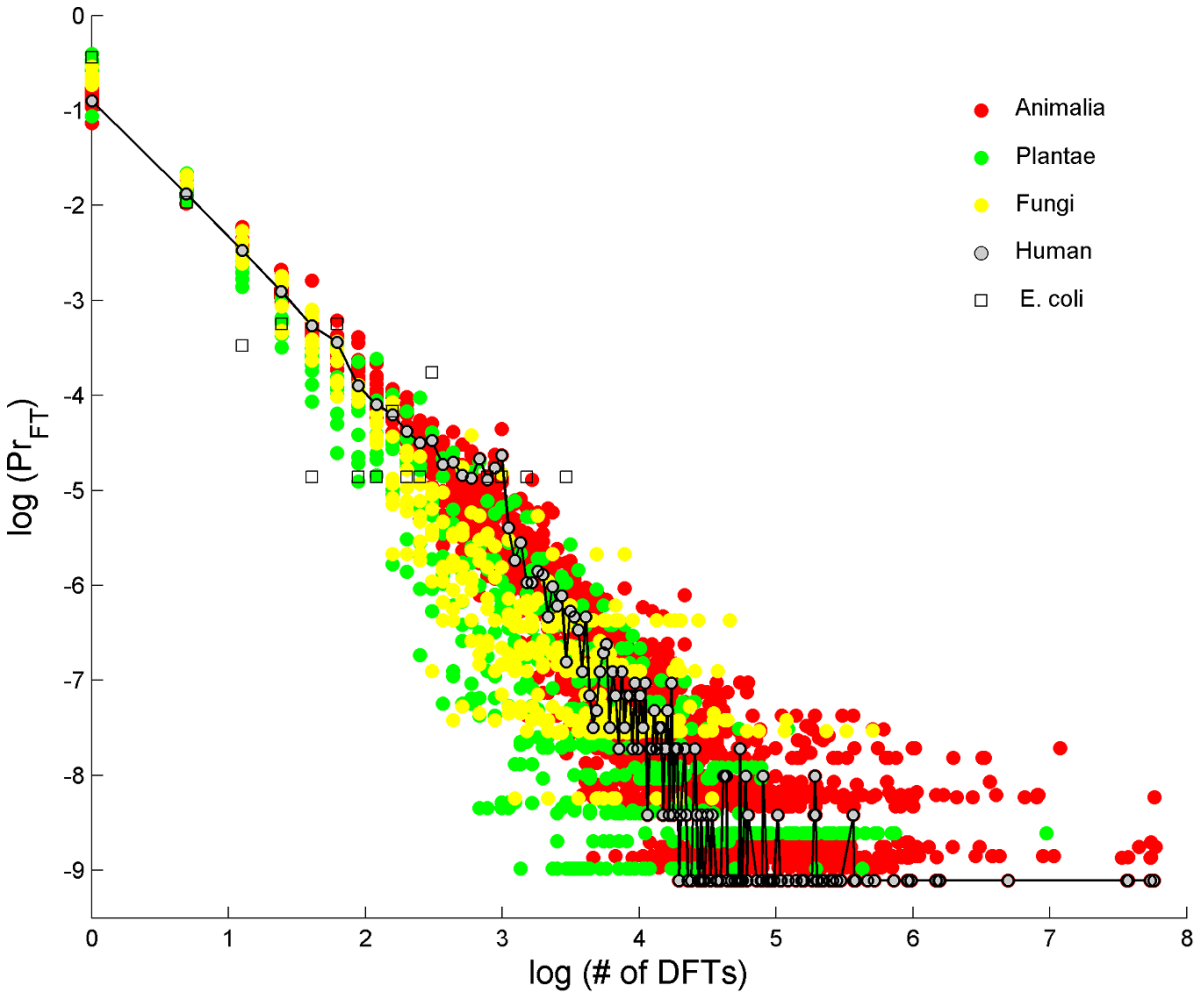
Universal DFT accumulation in proteomes. Probability of a number of DFT in a protein, on log-log scale, for 32 eukaryotes proteomes, colored differently for Animalia (red), Plantae (green) and Fungi (yellow). Few FTs occur quite often in the proteome while many FTs are rare. The cases of human and *E. coli* are shown as specific examples. All individual eukaryote species are very well fitted by a pure power-law (see Text S1 - section 9). *E. coli* serves as an example of a typical prokaryote.

The probability distributions of DFT abundance in the proteome are shown in figure 8 across 32 eukaryotes. They resemble power-laws for all individual eukaryotes (Text S1 - section 9, figure S16). Few DFT show up quite often on the proteome (examples are given in Table 6) and many DFT are quite rare, i.e. found on few proteins. Similarly, few proteins carry many DFT and many proteins carry much smaller numbers of DFT. Many of the prokaryotes seem to have similar DFT distribution behavior as well, but their variance is much larger. The specific example of *E. coli* added onto figure 8 serves to demonstrate the large variance observed for a single bacterium. Nevertheless, when individual distributions of many prokaryotes are superimposed, they reveal a power-law behavior as well (Text S1 - section 9, figure S17), suggesting universal characteristics. The general character of power-law distribution is similar to evolutionary genome universals such as the membership in paralogous gene families [1] and node-degree relations in biological networks [51]. Figure 8 may therefore add a new law to the ‘laws of genome evolution’ [52], this time at the peptide level. Further support for this view comes from observations of spontaneous expansions of triplets in higher taxa [35].

#### Universality of CO measure characteristics

**Figure 9:**
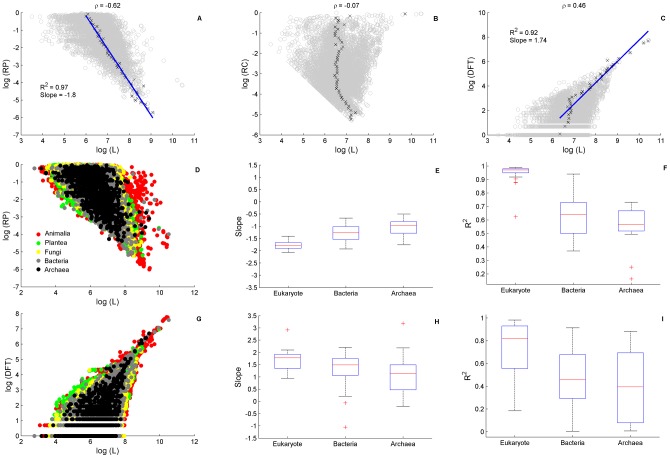
Universal dependence of RP and DFT on protein length. The relationship, on a log-log scale, between the CO measures RP, RC and DFT and protein length, L. Upper panel (A–C) display human proteins indicating strong correlation of RP (A) and DFT (C) but not RC (B), ρ indicated the Pearson correlation coefficient. A clear linear boundary in RC is due to its lower bound *3/L*. Linear regression analysis shows excellent power-law fits of RP and DFT dependence on L. Data was binned to 50 equally spaced intervals along the y-axis. ‘X’ symbols denote the average of L in each bin, error (SD) on the mean is at the size of the symbol and therefore not shown. The blue line is the result of a linear regression fit. Middle Panel (D–F) shows a superposition of RP-L data for all species (D) and the quality of its linear regression fits in (E,F). Slopes increase from Eukaryote to Prokaryotes (E) coupled with a decrease in the goodness of fit (F). Lower panel (G–I) is the same type of analysis for DFT-L dependence. Note that the slope trends are opposite. The ratio of the RP-L and DFT-L slopes is close to −1 in all species: it is −1.11±0.05 in eukaryotes. In prokaryotes, excluding 9 outliers, the ratio is −0.85±0.05.

Studying the behavior of CO measures we find that in single proteins RP and DFT show universal dependence on protein length *L* (figure 9). As an example, we show in figure 9A that RP for human is negatively correlated with *L*, while figure 9C shows that DFT for human is positively correlated with *L*. RC has no significant correlation with *L* (figure 9B). The left boundary in RC reflects its lower bound *3/L*. The distributions (over *L*) peak around the centers of their respective domains, and their averages may be well described by linear regressions. Figure 9D displays the behavior of RP for all species, while figure 9G is its analog for DFT. The universal behavior of these trends is quite obvious, as shown by the linear regression slopes for the average of these measures over all kingdoms in figures 9E and 9H, respectively.

Because FTs are not expected at random (see Methods), the behavior displayed in figures 9C and G implies that growth of protein length and CO are linked. This suggests the possibility that incorporation of CO may be an element of the mechanism of protein elongation. The decrease of RP, which is defined as the fraction of all FTs that participate in the MFI, may occur in one of two ways: either by increasing FT occurrences, as hinted by the increase in DFT, or by a decrease in the number of FTs participating in the most prominent periodic structure. Assuming that many of the longer proteins may be considered to be of older evolutionary origin [53], the decrease in RP could be blamed on mutations that were accumulated during evolutionary history. A direct analysis of the relationship between RP and protein age shows that high RP is associated with relatively young protein age (Text S1 - section 12, figure S20).

Another feature of universality is provided by the rank-ordered interval distribution functions. The latter is reminiscent of the Zipf-law (Text S1 - section 9, figures S18, S19), a hallmark of many dynamic evolving systems [54], as well as languages where word frequencies follow this law [55].

## Discussion

Many efforts to order major taxa according to known genomic measures are inconclusive [49; figure S6]. Aside from clear differences in the karyotypes, the best genomic discriminating factor between eukaryotes and prokaryotes is the prevalence of amino acid repeats in the former [26]. Their role in evolutionary processes, in particular fast evolution of protein function and development of phenotypic complex traits is well accepted [11]–[16]. However, because of the large diversity of repetitive sections, it is difficult to find genomic variants and determinants that may further elucidate their importance. Existing methods are usually tuned to capture a few aspects of the nature of compositional order, but a unifying framework has been missing [56].

Here, we presented such a unifying framework, by generalizing the concept of compositional bias to that of compositional order (CO), which captures all scales of repetitive peptides, from runs to repetitive domains. This is achieved by identifying multiple occurrences of frequent triplets that are not expected at random. As such, their existence on protein sequences allows for detecting various patterns, and provides novel measures of order. We focused in this study on three CO measures: ‘regularity’, the relative coverage of FTs in proteins (RC), which correlates well with sequence entropy; ‘periodicity’ of FT recurrences (RP); and FT-vocabulary, the number of different FTs (DFT), representing the richness in the vocabulary of CO on a protein or within a proteome.

### RC, RP and evolution

RC and RP provide novel perspectives on the evolution of proteins and proteomes by putting various observations into a common framework and applying comparative analyses. One of the astounding facts is the CO enrichment of novel proteins in human compared to mouse, leading to an increase in RC and RP. To a large extent, the latter is due to the large increase of ZF proteins in the human lineage.

Whereas an increase in RP is correlated with a general increase of the CO component of the proteome, we observe that a decrease of RP reflects the effect of mutations along the microevolution of a lineage. Rapid evolution by high mutation rate will tend to erase the periodic nature of repetitive sections in protein sequences. Thus, when comparing CO orthologous proteins in human and mouse, we find that some of the latter exhibit a clear decrease in RP, which may be blamed on the higher substitution rate along the mouse lineage. Our analysis of interval distributions is consistent with faster evolutionary substitution rate on the mouse lineage [45], [46]. A similar conclusion that RP functional enrichment of proteins deteriorates with evolutionary age of the organism follows from the study of response and extracellular proteins, for which RP-enrichment was seen to decrease from human through *A. thaliana* to *S. cerevisiae*.

When a protein's RP decreases along evolution this is evidence that the particular period, rather than its harmonics, may be less important to its function. Interesting cases are collagen and keratin proteins in human and cell wall proteins in plants, fungi and bacteria. In these cases prevalent intervals reflect the existence of underlying repetitive motifs, but protein enrichment with respect to RC and not RP indicates the tendency of these sections to rapidly accumulate mutations for functional purposes. Thus RC, rather than RP, correlates with these functions.

### Universality of the CO measures

We note that the balance between the three forces of evolution, mutation selection and innovation, acting on CO sections in proteins is universal. This we conclude from three observations:

1. the power-law distribution of DFTs in proteomes (figure 8). Interestingly, the accumulation of FTs in the proteome is similar to evolutionary genome universals such as the membership in paralogous gene families [1]. Such power-law behavior is observed in node-degree relations in biological networks [51], suggesting a particular role in protein-protein interaction (PPI) and metabolism, networks of similar functional node-degree architecture. Indeed, various metabolic processes, notably of macromolecule synthesis, are found in our human CO set. Other evidences for the role in PPI comes from the observation of CO enrichment in PPI hubs proteins [57], and the association of variations in repetitive sections with the evolution of PPI network topology [58].
2. the distribution of intervals resembling in character to Zipf's law (figure 1B). Zipf's law, the rule of word frequency in text, is a hallmark of linguistic structure [55]. The intervals between FT recurrences correlate with the lengths of larger motifs (see, e.g. the ZF protein PRDM9 in table 1), thus their frequency represents motifs frequency to a large extent. The appearance of Zipf's law also suggests fast evolution of motifs, such that there is no characteristic length scale of motifs, i.e. the distribution of FT intervals is scale free. In analogy of conventional interpretation of Zipf's law [59], our analysis suggests fast evolution of immune system and response related proteins in human, as well as cell-wall and response proteins in plants, fungi and bacteria. This strengthens the view that CO vocabulary is a hallmark of evolving diverse functionalities, a consequence of the necessity of some proteins to interact and adapt to fluctuating environmental conditions.
3. the relationships of RP and DFT with protein length (figure 9). RP decreases while DFT increases with protein length, in all species. The ratio of the power-law exponents of RP and DFT is approximately constant, indicating a balance between repetitive structure degeneration and CO vocabulary escalation. The positive correlation between RP and protein age (figure S20) implies that insertion of raw repetitive material is a possible mechanism responsible to protein growth. This also further associates RP with relatively new functions.

### DFT and macroevolution

The observation that DFT counts increase from archaea to vertebrates, providing a clear delineating hierarchy of major clades of organisms, is a unique case of correlating proteomic information with evolution. Since it relates to major taxonomic groupings, the evolutionary context to which it belongs is macroevolution [60]. Macroevolutionary changes are invariably connected to major genomic changes. Novel taxa and novel functions are marked by gene and chromosome rearrangement [61], and gene duplications [62] which may occur even after speciation [63]. This is also when major effects may occur in CO properties, as reflected by DFT counts. Thus we posit that changes in DFT reflect macroevolutionary events. In other words, we envisage major CO accumulation to occur mostly during macroevolutionary events. The following microevolutionary forces of mutation and selection can diminish or modify the CO, leading to the presently observed structures.

Eldredge & Gould [64] emphasized that long periods of small evolutionary changes are intertwined by relatively short periods of major changes, a phenomenon they called punctuated equilibrium. The Cambrian explosion period is a striking example, where changes from unicellular to multicellular species occurred within few 10 MY after billions of years dominated by microevolution. More recently, large-scale analysis of various measurements combining data from fossil records [65] showed that macroevolutionary steps indeed occur in rare bursts at time scales >1MY, presumably as a consequence of permanent changes in ecological and environmental properties [66]. Thus we should expect that major changes of DFT counts have occurred at relatively short periods of time, while most of evolutionary history accounted for smaller changes that accumulate during microevolution.

Gould [67] pointed out that one should not be influenced by our parochial focus on human, believing that evolution proceeds in the direction of complexification, since speciation may just as well take a turn toward simplification. Nevertheless, even if macroevolution can go both ways, it must still be true that high complexity of an extant organism, as well as a high DFT count, is a good indicator that its lineage has gone through many steps of macroevolution. CO structures that we observe on proteomes had survived while being modified by mutation under selection constraints. This suggests that analogously to birth-death-innovation models of protein domains evolution [68], similar forces shape the evolution of repeats at the peptide level.

In prokaryotes we find that DFT counts do not discriminate between major phyla. We observe, however, a clear distinction between mesophiles and thermophiles, suggesting that CO generation and conservation is also condition dependent. Thermophiles have characteristically lower DFT counts. The ones that we observe presumably have been selected for functional purposes. Evidence for this is the crucial role of CO in the induction of necessary structural changes under extreme conditions [48], and the prevalence of functional peptide motifs in extremophiles [69].

DFT content, rather than DFT counts, serves as another handle on proteomic relationships. Boundaries of prokaryote phyla may be discerned by their DFT content dissimilarity. This result is of particular interest when compared to previous attempts to find amino-acid sequence correlates of kingdom and super-kingdom divisions. Using information about single, double and triple amino-acid distributions, Pe'er *et al.* [33] observed some separations in a principal component analysis. Triplets turned out to be the best distinguishing elements. In our analysis, also based on triplets, but constraining them further to fit into FTs, we find that DFT correlations are highly significant, exposing DFT contents to be an important sequence correlate of kingdom identity.

Macroevolution is also affected by the landscape of inter-species interaction as in the case of plants and insects [70]. Here, the role of inter-species interaction is insinuated by DFT proximity of species in one kingdom to species in a neighbor kingdom, as exhibited by the similarity of fungal plant-pathogens to plants. Such interaction was previously suggested based on analysis of simple sequence repeats contents [34]. Interestingly, many of the CO proteins are response and immune related. Therefore, it is possible that these proteins coevolved as part of the interaction between the species defense systems. Thus, the fundamental factors that shape evolution of different lineages, i.e. unequal distribution of changes over time and correlation with ecological and environmental properties, also seem to shape CO composition at the kingdom level.

Lynch & Conery [71] studied the ordering of species based on the effective population size *x* mutation rate, N_e_u. While this measure is based on comparisons within species, i.e. it is of microevolutionary nature, their results suggest that increasing genomic complexity, associated with transitions from prokaryotes to eukaryotes, is a consequence of magnified random genetic drift. Comparing N_e_u with DFT counts over eukaryotes we find that they anti-correlate (Pearson correlation = −0.6, *P*-value≈10^−2^, Text S1 - section 11, table S4). This suggests that drift plays a central role in shaping FT evolution. Furthermore, we have seen that some CO in bacterial enzymes exhibit de novo creation which has presumably reached fixation through drift, without having any clear functional advantage, although we cannot exclude the possibility that these sections have emerged recently. However, there are various cases that are indicative of the effect of purifying and positive selection. Purifying selection is indicated by the very small numbers of DFT in thermophiles, while evidence for positive selection is provided by functional enrichment of various protein families with respect to increased CO (i.e., RC or RP), and by the mutations in synonymous and non-synonymous sites (Text S1 - section 10, table S3). To better quantify the relative contribution of positive and negative selection in specific protein families, one should resort to studying the traditional dN/dS ratio.

In summary we claim therefore that the FT tools that we have introduced and studied have proved themselves as meaningful measures of biological investigation. Moreover, they turn out to be very useful in providing the means for specifying which features are correlated with different protein annotations, and how the latter can be studied in a comparative genomics perspective. We believe that the highlight of this formalism is the fact that proteomic DFT counts turn out to delineate correctly major biological kingdoms, thus leading us to posit that CO vocabulary is intimately linked with major evolutionary forces.

## Methods

There exist known mathematical tools that come to mind for studying compositional order. One is the Shannon entropy [3], [5], and the other is the Fourier transform. The first is low when a clear imbalance in multiplicity of different amino-acids occurs, and the second should provide peaks for dominant periodicities. But the question remains what should be the basic variables. Entropy misses out on the co-occurrence of amino-acids in repetitive *k*-mers, while Fourier decomposition is much too noisy to allow for useful analysis. The difficulty and the need in constructing new and more general characterization of repeat patterns have been recently emphasized in a survey of existing methods [56]. Here, we establish a unifying framework for studying **all types of compositional order (CO)** within protein sequences. The basis for our systematic study is the identification of multiple occurrences of amino-acid triplets that appear far beyond random, which we define as Frequent Triplets (FTs). FTs allow for defining CO observables that facilitate the quantification and identification of structural elements of CO.

### Definition and analysis of frequent triplets (FTs)

**Figure 10:**
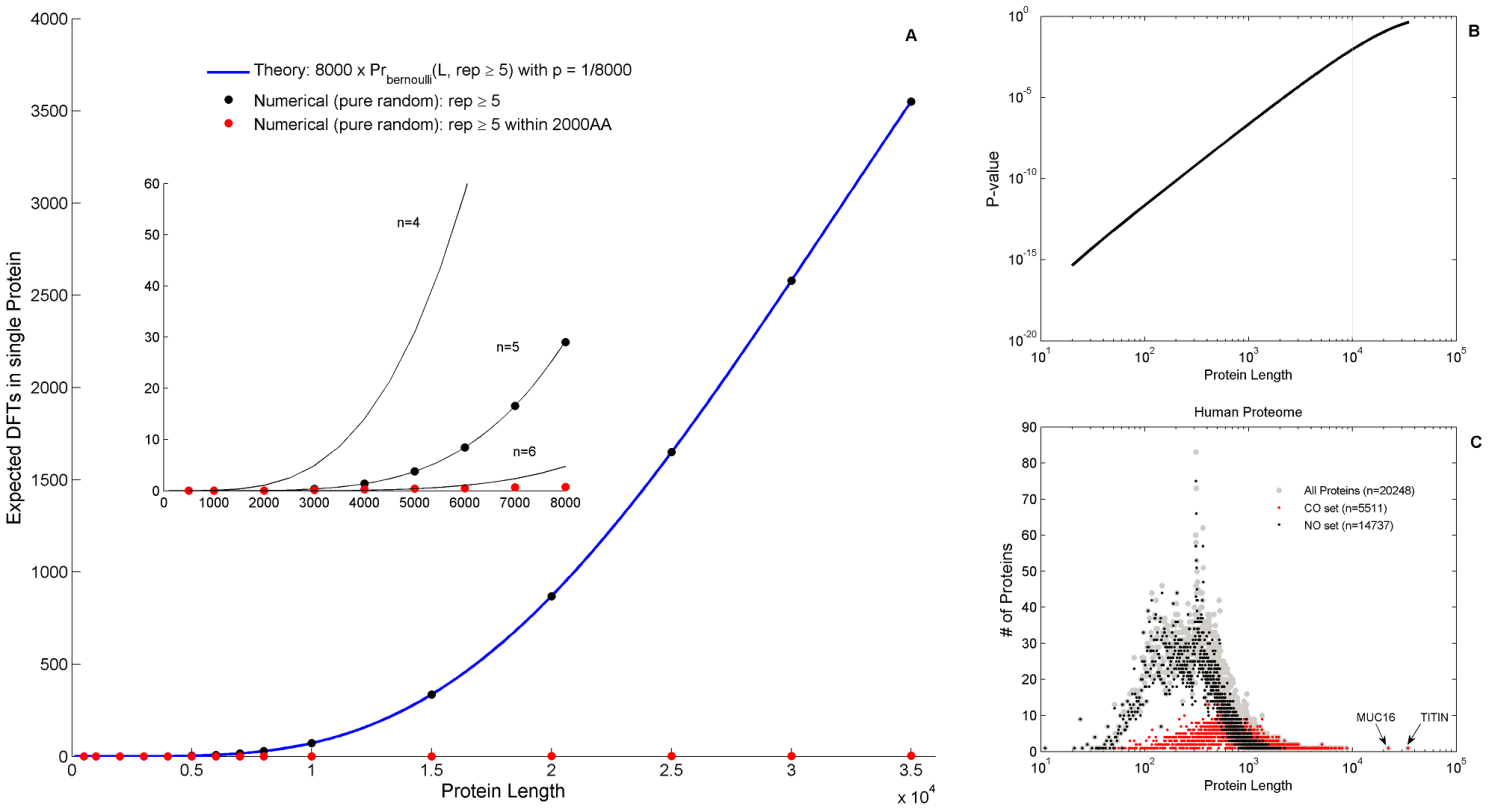
Frequent Triplets – Theory and simulation. Expected values of Frequent Triplets (FTs) in random proteins as function of sequence length. Length range is up to 35,000 amino-acids, approximately the length of the longest proteins found among the proteomes of the 94 species studied (TITIN in human, and beta-helical in *Chlorobium*). A) Blue curve is the theoretical expected value given by the Bernoulli probability, for *n = 5*. Dark circles are the corresponding results of a numerical search of triplets showing perfect match to the theoretical estimation. Red circles are the numerical results for restrictive FTs defined by *n = 5* and *M = 2000*. Inset: same data is shown up to *L = 8000* for clarity. Additional black curves represent the theoretical estimation for *n = 4–6*. B) *P*-value for FT misidentification as function of length on log-scale. C) Length distribution of human proteins showing log-normal characteristics. Length of CO proteins is right-shifted (see also Text S1 -section 3, figure S6d). Further analysis based on a human “unigram” reference model is provided in Text S1 - sections 1 and 2, where the few very long proteins are analyzed in detail.

Triplets of amino-acids represent a set of 8000 elements. The multiple appearance of a single triplet on a protein should have been a rare event had the sequence consisted of an independent arrangement of amino-acids. The probability to observe any triplet exactly *n* time in a sequence of length *L*, is given by the Bernoulli distribution: 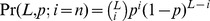, where *p* = 1/8000 in a uniform random model. The expected value, *E*, of the number of different triplets that appear at least *n* times in a protein is therefore:
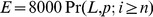
(1)thus, E/8000 is the *P*-value for FT misidentification. Numerical search for the occurrences of every one of the possible 8000 triplets on purely random protein sequences perfectly matches this theoretical estimate (figure 10). For the vast majority of proteins *n = 5* is sufficiently restrictive for eliminating any random occurrences while maximizing the signal, the number of identified FTs. However, for very large proteins (*L>8000*) *E* becomes quite large. Although the number of such long proteins is very small (3 in human, TITIN, MUC16 and SYNE1), a more restrictive definition that reduces *E*, can be formulated by requiring that repetitions (≥*n*) of a triplet should occur **within a section of length** ***M*** on the protein's sequence. The latter may become useful for the analysis of long proteins.

In Text S1, we present both the uniform random model of Eq. 1 and a unigram random model based on first order statistics of all human proteins (section 1), and provide detailed analysis of the human proteome comparing it to the two random models for various values of *M* (section 2). The analysis of random models suggests that a regular assignment of a triplet as FT, using *n = 5* yields *P*-values*<10^−3^* for proteins with *L<8000* and is sufficient for any practical use. The fact that the regular definition captures high-order structures in long proteins that are otherwise missed by restrictive definitions provides further justification for the use of regular FTs (Text S1 - section 2, figure S5). Thus, we define Frequent Triplets (FT) of a protein to be those amino-acid triplets that are observed to occur five times or more, not necessarily as tandem repeats, on its sequence. FT of a species proteome will be any FT that appears in at least one protein of this species.

Setting a threshold *n* for FT definition allows comparing CO between different species. With *n = 5* a reasonable description of CO can be obtained for all eukaryotes and prokaryotes. Lowering the threshold one runs into the problem of random effects, while raising the threshold is too restrictive (figure 10, inset) ending up with almost no FT assignments for some prokaryotes. We find that 27% of the human proteins are CO, i.e. contain FTs, while this fraction reduces to 17% in yeast and to 3% in *E-coli*. The fraction of CO proteins in the proteome is shown in Text S1 (section 3, figure S6b) for the main taxonomic groups we study. Similar orders of magnitude of ordered sequences are obtained by other methods [26]. Our methodology has another important outcome for comparative analysis: it tends to unify the mean compositionally ordered protein length across the tree-of-life (Text S1 - section 3, figure S6 c,d). In other words, CO proteins display higher length similarity between eukaryotes and prokaryotes than average lengths defined over their complete proteomes.

### Measures of compositional order (CO)

Many protein sequences show significant periods or quasi-periodic repeats. We wish to distinguish between the two aspects of ***regularity*** and ***periodicity***. This is because both can be viewed as orderly elements of a protein sequence, as in many tandem repeats of any *k*-mer, but they are not necessarily correlated.

#### Regularity

Sequence regularity is traditionally estimated by the single amino-acids entropy [3], [5]. The Shannon entropy can be generalized, using as its basis any *k*-mer of amino-acids, as follows
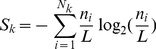
(2)Where *N_k_* is the number of possible *k*-mers in a sequence, *N_k_ = 20^k^*; *L* is the length of the sequence and *n_i_* is the number of occurrences of the *i_th_ k*-mer in the sequence. *S_k_* measures sequence regularity, and is low when a clear imbalance in *k*-mer multiplicity occurs. For comparative analysis, it is useful to normalize the entropy by it maximal value, defining the normalized entropy, *nS_k_*. This describes by how much a given sequence differs from a random sequence of maximum entropy. For *k = 1*, *L≫N_1_*, the maximum of *S_1_* is *log_2_(20)*, and the normalized entropy is given by *nS_1_ = S_1_/log_2_(20)*. For *k>2*, usually *N_k_≫L* thus the normalized entropy is given by *nS_k_ = S_k_/log_2_(L)*.

Considering all FTs that appear on a given protein sequence, we define their **‘relative coverage’ RC** of this particular protein as the number of distinct amino-acid loci covered by FTs divided by the protein length. This parameter correlates significantly with the normalized entropy *nS_3_* (Text S1 - section 4): high RC implies very distinctive CO, hence also very low *nS_3_* entropy. Thus, RC is a good tool for estimating compositional bias. Note that the dynamic range of RC is considerably larger than that of *nS_3_* (figure S7 C), thus making RC an easier tool to use.

#### Periodicity

Many CO sequences show significant periodic or quasi-periodic repeats (table 1). Entropy does not provide information about the periodicity of a sequence, neither does RC. We introduce measures based on the **intervals** between all consecutive occurrences of the **same** FT, *I_FTi_*. Considering all consecutive intervals of each FT, *I = {I_FTi_}*, one obtains the discrete empirical probability distribution function of all consecutive intervals, *P(I)*. We define the **Most Frequent Interval (MFI)** of a CO protein as:
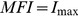
(3)where *I_max_* is the interval at which *P(I)* is maximal. Because MFI is based on the intervals of all FTs, and not on the intervals of the most repetitive single FT, it is a robust estimator. Rarely, there are two or more intervals with equal maximal probability, in which cases MFI is taken as the lowest interval, because it is often also the lower harmonic of the higher MFI. The significance of MFI may be further evaluated by considering the number of interval occurrences at MFI (see figure 1c).

Based on the MFI we define the **‘relative periodicity’ RP** as the number of FT occurrences within the MFI divided by total number of FT occurrences:
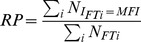
(4)RP measures the relative richness of CO observed within the periodic structure, compared to the overall CO observed in the sequence, and approaches zero as MFI becomes less significant. RP has no significant correlation with neither RC nor *nS_k_*, and therefore contains independent information (Text S1 - section 4 and figure S7).

#### Vocabulary

We define a measure of FT-vocabulary as the total number of Different Frequent Triplets (**DFT)**. This is the count of FT types rather than the number of FT occurrences, and can be applied either to single proteins or to a full proteome. The correlation between two DFT sets *I*, *J* representing either two proteomes or two proteins is estimated by the Jaccard score *C_IJ_ = (DFT_I_∩DFT_J_)/(DFT_I_ ∪DFT_J_)*, the size of the intersection divided by the size of the union of the sample sets.

### The case of overlapping triplets

Triplets may overlap. For example, in a protein containing the sequence AAAAAAA there are 5 occurrences of the single FT AAA, lagged by interval 1 and of total coverage of 7 amino-acids. This defines an MFI = 1 case, here a run of A. Similarly, MFI = 2 is a property of tandem repeats such as AQAQAQAQAQAQ, composed of two FTs (AQA and QAQ) that occur 5 times each, separated by interval 2 and with total coverage of 12. The occurrence of overlapping triplets is highly reduced in random models. Hence, one could have submitted runs to a constraint weaker than *n = 5*. This has little effect on global analysis, hence we stick to a single definition using always *n = 5* as the minimal number of occurrence of an FT. As a result proteins which have only short runs (<7) are excluded from our set of CO proteins.

### Relation to other repetitive phenomena and motifs characterization

Several tools have been developed for analyzing tandem repeats; both at the DNA level [72] and at the protein level [2], [73]. While such methods may provide detailed local information on repeat properties, such as unit length, purity, and alignment-based repeat-variations, they are applicable to a subgroup of CO proteins with particular periodic structures. Moreover, such methods usually involve more than one stage of filtering and therefore require further choices of internal parameters to be able to account for each repetitive phenomenon such as tandem repeats or cryptically simple repeats. Our CO measures provide global information and are not restricted to a specific type of periodic structure. They span the spectrum of possible high-order structures, and therefore are not always directly comparable to other existing motif characterizations.

Nevertheless, within high RP proteins one finds cases of tandem repeats, for which the CO measures can account for some basic motif properties, such as unit length and purity. In a highly pure motif repeat pattern, for example PRDM9 (Table 1), the identified DFTs compose the motif of unit length 28. Thus, DFT = MFI indicates a highly pure motif. Even if part of the motif is variable, the identified FTs can appear in equally distant intervals leading to high RP, and MFI still indicates the unit length. Thus, RP is independent of motif purity, which may be accounted for by the difference between DFT and MFI. DFT<MFI indicates a decrease in purity. Note that DFT>MFI corresponds to larger regularity than periodicity, as in some low-complexity regions, and will usually indicate low RP but moderate or high RC.

### Functional enrichment

For large groups of proteins we employ the bioinformatics tool provided by GOrilla [39], [40]. In addition, to explore possible dependencies on CO measures, enrichment levels were estimated by text search of key-terms in GO annotations. Specifically, we estimate *N_tot_*, the total number of proteins with order measure value>some threshold. For a given threshold, we obtain the number of proteins that are associated with a certain GO term, *N_fun_*. The dependence of the ratio *N_fun_/N_tot_* on the threshold provides an enrichment pattern which allows for quantifying dependencies on CO measures also for small functional groups. Enrichment *P*-values are estimated by the Hypergeometric test, comparing the values of *N_fun_* and *N_tot_* obtained at the maximum of the ratio *N_fun_/N_tot_* with their values at the minimal threshold. To avoid small number effects, *N_fun_* is limited to 10% of the maximal *N_fun_* (i.e. at the minimal threshold), and in any case *N_fun_*>15, such that there exists sufficient data for statistical inference of enrichment levels.

### Data bases

Full proteomes of 94 species distributed across the tree-of-life were downloaded from NCBI Ref-Seq, and served as the basis of the large-scale analysis of compositional order enrichment. Swiss-Prot reviewed proteomes of several representative species from animalia (human, mouse), plantae (A. thaliana), fungi (*S. cerevisiae*) and bacteria (E. coli) were downloaded and analyzed as well. For *S. cerevisiae* we compared in addition the Swiss-Prot proteome to SGD data-base for validation of proteins annotation. Generally, Swiss-Prot contains fewer proteins than NCBI. It provides however high quality information about proteins' biological functions, and contains non-redundant sets. Therefore, we used Swiss-Prot for detailed protein functional analysis, and also to test the sensitivity of CO measures to the choice of data base (see the following sensitivity analysis).

### Sensitivity analysis

#### Interval distributions and the parameters specifying an FT

We have opted for the use of amino-acid triplets, i.e. *k*-mers with *k = 3*. Moreover, we have defined an FT using a constant threshold for its number of occurrences, *n = 5*. In Text S1 (section 5) we have tested other alternatives, i.e. different values of *k* and different thresholds that depend on protein length. The results, presented as rank-ordered interval distributions in figure S8, show that the regular FT-definition is optimal for capturing large repetitive motifs that are missed by alternative definitions of a frequent *k*-mer.

#### Proteomic DFT enrichment in different databases

Comparison of the Swiss-Prot human proteome (20,248 proteins) with ref-seq data (34,084 proteins) by reconstructing the list of Different Frequent Triplets (DFT), showed less than 0.1% reduction in the number of DFTs in spite of the large difference in numbers of listed proteins. This exemplifies the redundancy of proteins in ref-seq data, and proves that the DFT count is a stable measure. In mouse, where Swiss-Prot lists only 16,513 proteins, compared to 28,868 proteins in ref-seq, we found a reduction of 10% in Swiss-Prot DFT counts. This seems to indicate that the Swiss-Prot mouse proteome is incomplete.

Ref-Seq DFT counts of different species are provided in Table 5, grouped into kingdoms. There exist outstanding DFT counts in prokaryotes, such as for *C. chlorochromatii*, where few extremely long genes are responsible for the FT assignment of more than half the DFT. Such exceptions occur in prokaryotes and account for the outliers in figure 5. However, no such case exists in eukaryotes. Even TITIN, which carries many FTs, is responsible for the FT assignment of less than 2% of all DFT in human. Further analysis of the sensitivity to long proteins is provided in Text S1 (sections1–2).

### Graphic User Interface for practical research of CO

We provide a friendly GUI MATLAB package that implements the search of triplets in proteins to help interested practitioners using our method for further research. The GUI accepts proteins in FASTA format. Both single proteins and full proteomes can be uploaded. The GUI allows changing the key parameters *n* and *M*. The output is a list of CO proteins and their CO properties, that is automatically saved as TEXT file and can be accessed with MicroSoft EXCEL. Key properties and figures are presented in the GUI output. The GUI is available at: http://neuron.tau.ac.il/~horn/research.html

## Supporting Information

Figure S1 **Human proteins length distribution.** Protein length distribution of the CO (black) and NO (gray) sets using our ***regular*** FT definition. The vast majority of NO proteins have length well below 2000 amino-acids. Three additional proteins beyond the scale of 8000 amino-acids (SYNE1, MUC16, TITIN) belong to the CO set.
(TIF)

Figure S2 **Frequent triplet (FT) identification in random models of protein sequences.** The expected value, *E*, of the number of identified FTs in a single protein (left y-axis) and the *P*-values (estimated as *E*/8000) for misidentification of a single FT in a single protein (right y-axis) are shown for a uniform model of protein sequences with alphabet of 20 amino-acids (A–B) and for a unigram model generated from the human proteome (C–D). A, C correspond to a search of any triplet that appears at least 5 times on a single protein. B, D correspond to a search of any triplet that appears at least 5 times on a single protein within a section smaller than 2000 letters (*M = 2000*). Errors on the expected values are smaller than the symbol sizes (based on numerical simulation of thousands of non-overlapping proteins sampled from the two models).
(TIF)

Figure S3 **Variation of CO properties as function of** ***M*** **in human and random models.** Numerical search of triplets in human Swiss-Prot proteome containing 20248 proteins and in two random models (uniform and unigram) with identical protein length distributions to human at various values of *M* shown in the x-axis. A) The number of identified CO proteins, *N_CO_*, at various M are presented as fraction of *Nco = 5511* at *M = Inf*, showing saturation of the identified CO set at *L>1000*.B) The fraction of proteins for which the identified FTs at various *M* differ by *Δ* from the identified FTs at *M = Inf*. The black bar represents the case where *Δ>0*, i.e. considering all proteins for which 1 FT difference or more was measured. At *M = 2000* this fraction is approximately 5%. Red bars represent the case of *Δ>1%* of the number of FTs identified at *M = Inf*. This fraction is smaller than *5%* for all *M*. C) *N_CO_* found in a uniform random model compared with *Nco* found in human proteome, showing a minor fraction of <3%. D) regular DFTs of human for various *M* (black bars), compared with those obtained for the uniform random model (red). 2 long proteins (TITIN and MUC16 of length 34350, 22152, respectively) may contribute a large number to the total DFTs found in the proteome. However, excluding them reduces DFT counts considerably in the random model (green) but not in the human proteome (gray). This shows that the human proteome is not sensitive to the contribution of few proteins, even the very long ones, in contrast to the random model. It also shows that the number of erroneous regular FTs in long proteins may be large and should be investigated separately. E–F) same as C–D for the human unigram model.
(TIF)

Figure S4 **Dependence of DFT counts in the proteome on single protein contribution.** Proteins were sorted by the number of FTs identified in them according to the regular (A) and restrictive definition (B) in human proteome (black), in uniform model (red) and in unigram model (blue). Long CO proteins were removed one by one (rank-ordered by length) from the set and the DFT count was reassessed. In A, the two long proteins (see text) contribute many DFTs in the random models, but not in human.
(TIF)

Figure S5 **Interval recurrences in TITIN and MUCIN-16 of human.** The interval distribution of human TITIN protein as obtained for both regular and restrictive FT definitions (A). This is compared to the regular FT definition results of uniform (B) and unigram (C) models of this protein. Random models do not show any significant interval recurrences while the TITIN protein has clear high-order structures. Same is shown for MUCIN-16 (D–F).
(TIF)

Figure S6 **Genomic measures by kingdoms.** A) Boxplots of the number of proteins in proteomes. B) The fraction of CO proteins, i.e. FT-containing ones in the proteome. The fraction in eukaryotes is generally higher than in prokaryotes. Within eukaryotes, the fractions are not correlated with phylogenetic distance or species complexity. C) The average protein length in proteomes showing considerable variability, with prokaryotes having the smallest average protein length. D) The average length of CO proteins tends to be higher and flat, showing little variability across the tree-of-life. Note the different scales in C and D. Species are grouped as in figure 5 of the main text, vertebrates (V) invertebrates (IV), plants (P), Fungi (F), Bacteria (B) and Archeae (A). Dataset was downloaded from NCBI ref-seq.
(TIF)

Figure S7 **CO measures.** Scatter plots of compositional bias entropy measures of single amino-acids and of triplets of amino-acids, the relative coverage RC, and the relative periodicity RP, for human (blue) and yeast (yellow). A) The relationship among the normalized entropies, *nS_1_* and *nS*_3_ (Pearson correlation is 0.61 in human and 0.76 in yeast). B) The relationship between *nS_1_ a*nd RC. C) RC is highly correlated with *nS_3_* (Pearson correlation of 0.93 in human and 0.94 in yeast). D–F) the relationship between RP and*nS_1_*, *nS_3_*, and RC, respectively. Correlation between RP and all these measures are very weak, indicating that RP provides independent information.
(TIF)

Figure S8 **Sensitivity tests based on the rank-ordering interval distributions.** Sensitivity test of human rank-ordering interval distributions for different *k*-mers (*k*) and repetition thresholds (*th*). Threshold is defined in terms of the ratio of the number of repetitions by the protein length. The proteins that are found to have *k*-mers that pass the threshold define a new CO set, which is compared with our original CO (defined by 5 repeats and *k = 3*). The percentage of overlap between the two sets is shown. Note that *k = 4*, *th = 0.1%*and the regular FT definition *k = 3*, *th = 5* provide approximately the same CO proteins, and their interval distributions are very similar: they practically overlap, except for their tails.
(TIF)

Figure S9 **DFT hierarchy evaluated with restrictive FTs.** Similar box plots of DFT counts across the tree-of-life to the one presented in figure 5, but using the restrictive FT definition. Each box delineates lower quartile, median and upper quartile values. Most extreme values (whiskers) are within 1.5 times the inter-quartile range from the ends of the box. Outliers are also displayed. Prokaryotes are displayed twice. First grouped according to bacteria and archaea, and secondly as mesophiles and thermophiles. *P*-values according to non-parametric two-sample Kolmogorov-Smirnov test are 2.5×10^−2^ (V-IV), 3.6×10^−3^(IV-P), 9.8×10^−3^ (P-F), 7.86×10^−6^ (F-B), 2.3×10^−2^ (B-A) and 1.38×10^−4^ (M-T). Protista species show large variability and cannot be distinguished from Plantae or Fungi by the DFT measure.
(TIF)

Figure S10 **Dependence on run length.** Certain GO terms that depend on runs length as measured by the number of repetitions at MFI = 1. Run length is associated with DNA-binding, regulation and transcription in A. thaliana (A) and S. cerevisiae (B). Bacterial enzymes show similar behavior of cell wall proteins (C).
(TIF)

Figure S11 **Interval shift in mouse.** Examples of orthologous sequences in human and mouse with low sequence similarity. Numbers followed by AA (green) indicate the numbers of amino-acid before and after the repetitive section. Within the repetitive section the leading FTs at MFI are highlighted (blue) and the number of amino-acids between recurrences of FTs is given in ( ) for visual convenience. These numbers allow for easy observation of the existence of “harmonics”.
(TIF)

Figure S12 **Differential Analysis of phase in human and mouse.** Comparison of orthologous CO proteins in human and mouse with MFI>1 and which have low sequence similarity ID<70% (n = 204). The most abundant FT at MFI (Flag) of human sequence (A) and of mouse sequence (B) were each searched in both species. The histogram of the difference in the average interval of the occurrences of a flag between the human and mouse protein pairs is shown. Leftward shift is estimated by the fraction of proteins that had negative difference compared with positive difference, i.e. measuring the extent by which regularity tends to disappear in mouse. Left shift factors yield 2.6 (A) and 0.7 (B).
(TIF)

Figure S13 **Comparison of runs in eukaryotes and prokaryotes.** Comparison of runs in eukaryotes and prokaryotes carried out for the leading 45 FTs, covering all species. The threshold of 45 was chosen because it is the minimal DFT count among all species. Plotted are the numbers of FTs composed of a single amino-acid, representing runs. The Box-plot demonstrates the significant abundance of runs in eukaryotes (*P*-value = 1.067×10^−16^, non-parametric two-sample kolmogorov-smirnov test).
(TIF)

Figure S14 **Clustergram of DFT correlation among human proteins.** Hierarchical clustering based on the matrix C_IJ_ of 5511 human CO proteins is shown at the top. Heatmap of C_IJ_ is shown at the bottom, revealing about 10 large clusters in addition to several small ones. The big group in the middle, around index 3000, contains mostly ZF proteins.
(TIF)

Figure S15 **Summary of DFT presence in the human proteome.** DFT are ordered according to their abundance in the human Swiss-Prot proteome (x-axis, main panel) and proteins are ordered according to the classification of the clustergram in figure S14 (y-axis, main panel). The abundance of each FT is shown in the upper panel (blue) and the number of DFTs in each protein is shown on the left panel (blue). On the right, zoom in into the 50 most prevalent FTs showing the co-occurrences in groups of proteins. Note that their rank is slightly different from Table 6, which is based on NCBI-RefSeq.
(TIF)

Figure S16 **Linear fits of the DFT probability distribution functions in eukaryotes.** Linear fits of the DFT probability distribution functions are shown for human (A), *A. thaliana* (B) and *S. cerevisiae* (C). A–C) black circles are the data points and colored dashed lines are the fits over range 0–4 of the x-axis. *P*-values are 2.5×10^−36^, 2.7×10^−32^, 3×10^−24^ in human, *A. thaliana* and *S. cerevisiae*, respectively. All eukaryote data have corresponding power-law fits with *P*-values smaller than 10^−17^. D) The slopes (i.e. the power law exponents) of all eukaryotes we have analyzed. The cases in A–C are indicated by arrows.
(TIF)

Figure S17 **Linear fits of the DFT probability distribution functions in prokaryotes.** A) Individual DFT probability distribution function of all bacterial species (n = 36) superimposed (black circles). Linear fit, for the range 0–3, was applied to all data points (*P*-value∼10^−177^, slope = −1.6). B) Same procedure applied for all archaea species (n = 19). Linear fit with *P*-value∼10^−65^ and slope = −1.35.
(TIF)

Figure S18 **Linear fits of the rank-ordered probability distribution functions in eukaryotes.** rank-ordered distribution function (black circles) and the corresponding linear fits over the range 0–6 are shown for human (A), *A. thaliana* (B) and *S. cerevisiae* (C) as colored dashed lines. Applying such fits to all eukaryotes we find that the power-law exponents are close to −1 (D), displaying a universal behavior that is close to the Zipf law. *P*-values in all cases are practically 0.
(TIF)

Figure S19 **Slopes of the rank-ordered probability distribution functions.** Slopes of all individual interval probability distribution functions as obtained by a linear fit. *P*-values among all species were lower than 10^−39^.
(TIF)

Figure S20 **Protein age** ***vs*** **RP.** The average age of proteins (black) is shown versus elevated RP. Error bars on the mean age are also shown. The statistical significance of the difference between the age distribution for a given RP threshold and the age distribution of the entire CO set was estimated according to Wilcoxon rank-sum test (red).
(TIF)

Table S1 **Full list of all Human CO proteins.** Detailed sequence information is provided for all identified Human CO proteins in the Swiss-Prot record.
(XLSX)

Table S2 **Characteristics of the 10 longest human proteins.** Characteristics of the 10 longest human proteins, displaying their length, assigned DFT counts according to the ***regular*** and ***restrictive*** definitions, and error estimates on the latter, given in ‘()’, for the uniform and unigram random models respectively. In the unigram model the inhomogeneous drop with decreasing length is due to statistical fluctuations. Last column shows that amendment due to periodicity considerations (see figure S5) increases considerably the number of validated FTs.
(DOCX)

Table S3 **Mutation and selection in cell wall proteins in yeast by DNA analysis.** Analysis of repetitive motifs in cell wall proteins of *S. cervisiaea*. First and second columns show the name, function and starting location of the repetitive section. Third and fourth columns show the sequences at the protein and DNA levels respectively. Motifs are divided into identical sections forming groups which are colored with gray (group 1), blue (group 2) and white (group 3). The first group is taken as a reference motif and the remaining groups are compared to it in the following way: Yellow colored letters indicate mutations that cause the generation of a distinct motif, i.e. amino-acids different from group 1. Red colored letters indicate synonymous mutations within each motif group, thus do not change the motif composition at the amino-acid level, indicating that some amino-acids are protected by selection.
(DOCX)

Table S4 **Comparison between DFT counts and N_e_u values.** Listed are all eukaryotes for which we could retrieve the N_e_u measure from Lynch & Conery 2003. Left: species are ordered according to DFT counts in ascending order. Right: Same species ordered according to N_e_u in descending order. Both measures provide an hierarchical ordering of major clades. They also significantly correlate (correlation coefficient = −0.6, *P*-value = 0.012). Clade notations are the same as in figure 5 of the main text.
(DOCX)

Table S5 **Non-orthologous genes and proteins in human and mouse using Ensembl.** For non-orthologous genes we compare the numbers of different genes, available protein coding sequences and the number of CO proteins, for five different data sets. 1) Known genes with RefSeq Protein IDs 2) All genes, i.e., including novel and putative, with RefSeq IDs 3) All genes with UniProtKB/TrEMBL accessions 4) All genes with Entrez IDs 5) no filter applied. The ratio between the number of CO human and mouse proteins is between 2 to 5. As found in Swiss-Prot, a large fraction of the novel CO human proteins are zinc fingers.
(XLSX)

Text S1 **Supporting analysis.** Description of all the supporting analyses (13 sections) that are mentioned in the main text.
(DOCX)

Text S2 **GOrilla analysis of Human CO proteins.** Results of functional enrichments in Human CO proteins using GOrilla web-tool. Enrichments of processes, functions and cellular components are shown for three analyses: (i) comparing the Human CO proteins (‘target’) to all Human proteins (‘background’) (ii) Ranking Human CO proteins by RC (ii) Ranking Human CO proteins by RP.
(DOCX)

Text S3 **GOrilla analysis of Human novel CO proteins.** Results of functional enrichments in Human novel CO proteins using GOrilla web-tool. Enrichments of processes, functions and cellular components are shown for (i) Human novel CO proteins ranked by RC (ii) Human novel CO proteins ranked by RP.
(DOCX)
