# Supplementary figures and images for: Systematic Analysis of Compositional Order of Proteins Reveals New Characteristics of Biological Functions and a Universal Correlate of Macroevolution
Source: PLoS Comput Biol. 2013 Nov 21;9(11):e1003346. doi: 10.1371/journal.pcbi.1003346 (PMC3836704; doi:10.1371/journal.pcbi.1003346)

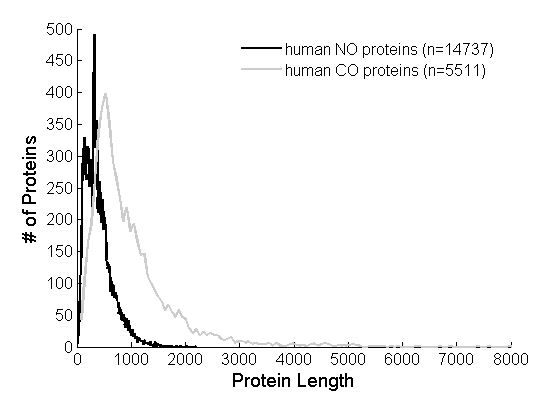

Supplement: Figure S1 — Human proteins length distribution. Protein length distribution of the CO (black) and NO (gray) sets using our regular FT definition. The vast majority of NO proteins have length well below 2000 amino-acids. Three additional proteins beyond the scale of 8000 amino-acids (SYNE1, MUC16, TITIN) belong to the CO set. (TIF) [file pcbi.1003346.s001.tif]

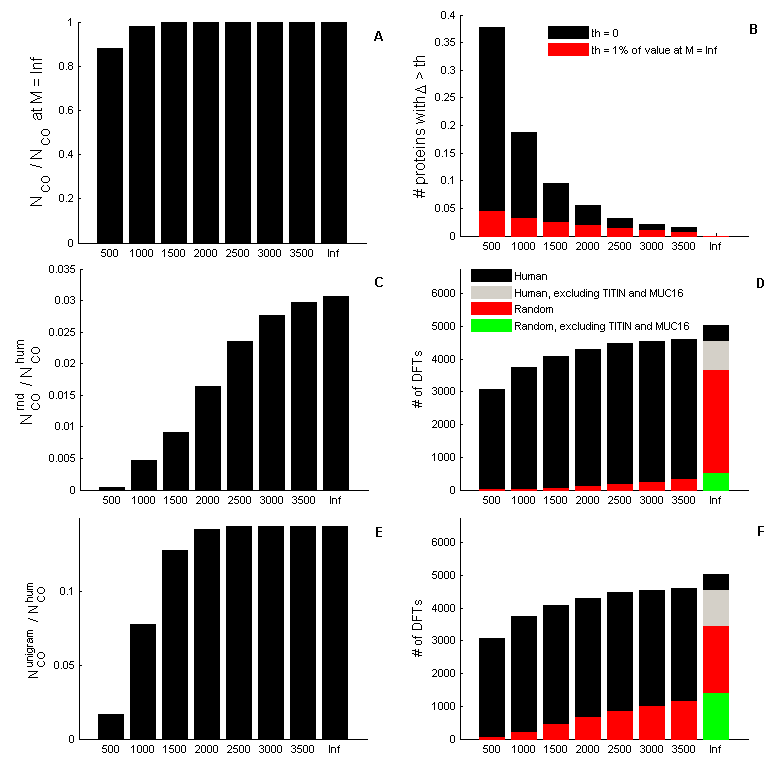

Supplement: Figure S3 — Variation of CO properties as function of M in human and random models. Numerical search of triplets in human Swiss-Prot proteome containing 20248 proteins and in two random models (uniform and unigram) with identical protein length distributions to human at various values of M shown in the x-axis. A) The number of identified CO proteins, NCO, at various M are presented as fraction of Nco = 5511 at M = Inf, showing saturation of the identified CO set at L>1000.B) The fraction of proteins for which the identified FTs at various M differ by Δ from the identified FTs at M = Inf. The black bar represents the case where Δ>0, i.e. considering all proteins for which 1 FT difference or more was measured. At M = 2000 this fraction is approximately 5%. Red bars represent the case of Δ>1% of the number of FTs identified at M = Inf. This fraction is smaller than 5% for all M. C) NCO found in a uniform random model compared with Nco found in human proteome, showing a minor fraction of <3%. D) regular DFTs of human for various M (black bars), compared with those obtained for the uniform random model (red). 2 long proteins (TITIN and MUC16 of length 34350, 22152, respectively) may contribute a large number to the total DFTs found in the proteome. However, excluding them reduces DFT counts considerably in the random model (green) but not in the human proteome (gray). This shows that the human proteome is not sensitive to the contribution of few proteins, even the very long ones, in contrast to the random model. It also shows that the number of erroneous regular FTs in long proteins may be large and should be investigated separately. E–F) same as C–D for the human unigram model. (TIF) [file pcbi.1003346.s003.tif]

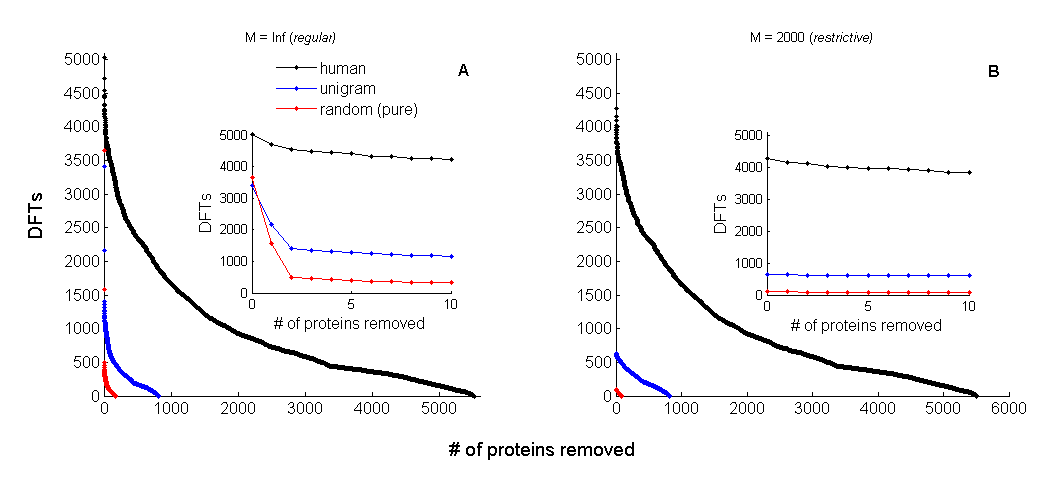

Supplement: Figure S4 — Dependence of DFT counts in the proteome on single protein contribution. Proteins were sorted by the number of FTs identified in them according to the regular (A) and restrictive definition (B) in human proteome (black), in uniform model (red) and in unigram model (blue). Long CO proteins were removed one by one (rank-ordered by length) from the set and the DFT count was reassessed. In A, the two long proteins (see text) contribute many DFTs in the random models, but not in human. (TIF) [file pcbi.1003346.s004.tif]

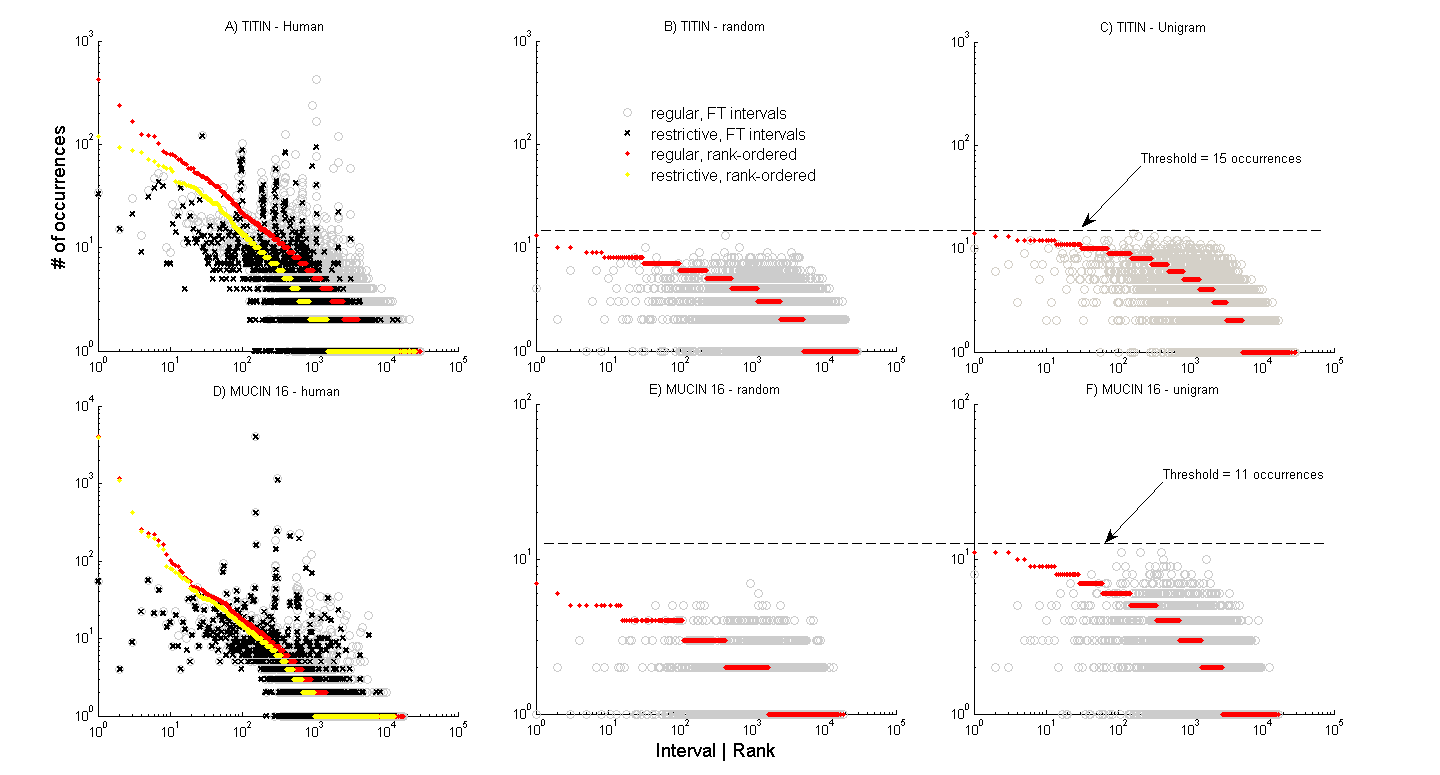

Supplement: Figure S5 — Interval recurrences in TITIN and MUCIN-16 of human. The interval distribution of human TITIN protein as obtained for both regular and restrictive FT definitions (A). This is compared to the regular FT definition results of uniform (B) and unigram (C) models of this protein. Random models do not show any significant interval recurrences while the TITIN protein has clear high-order structures. Same is shown for MUCIN-16 (D–F). (TIF) [file pcbi.1003346.s005.tif]

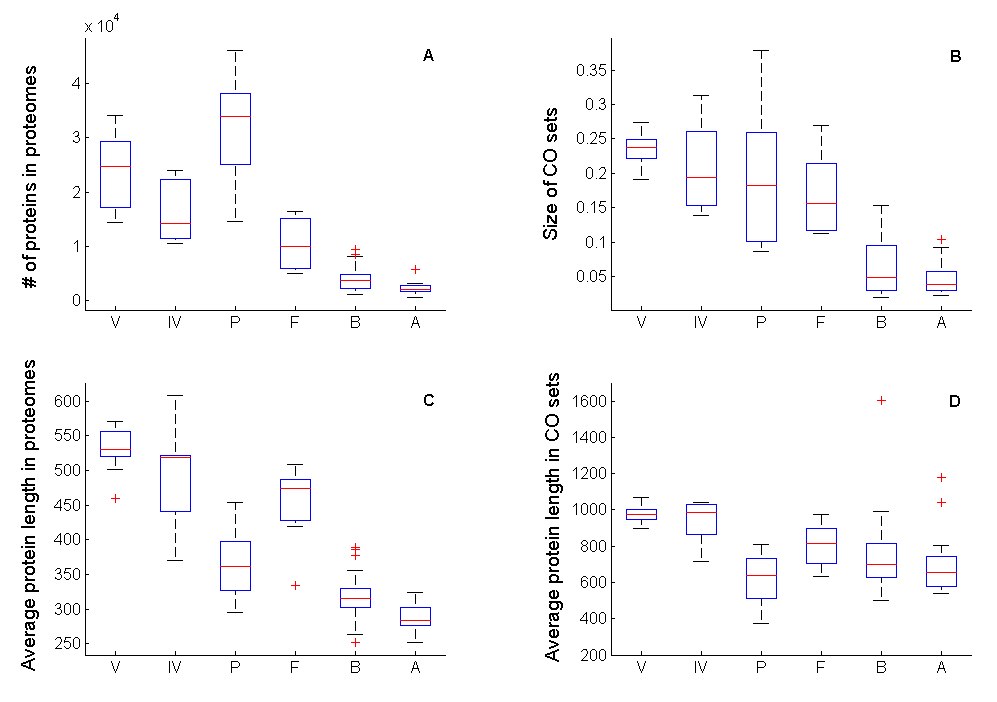

Supplement: Figure S6 — Genomic measures by kingdoms. A) Boxplots of the number of proteins in proteomes. B) The fraction of CO proteins, i.e. FT-containing ones in the proteome. The fraction in eukaryotes is generally higher than in prokaryotes. Within eukaryotes, the fractions are not correlated with phylogenetic distance or species complexity. C) The average protein length in proteomes showing considerable variability, with prokaryotes having the smallest average protein length. D) The average length of CO proteins tends to be higher and flat, showing little variability across the tree-of-life. Note the different scales in C and D. Species are grouped as in figure 5 of the main text, vertebrates (V) invertebrates (IV), plants (P), Fungi (F), Bacteria (B) and Archeae (A). Dataset was downloaded from NCBI ref-seq. (TIF) [file pcbi.1003346.s006.tif]

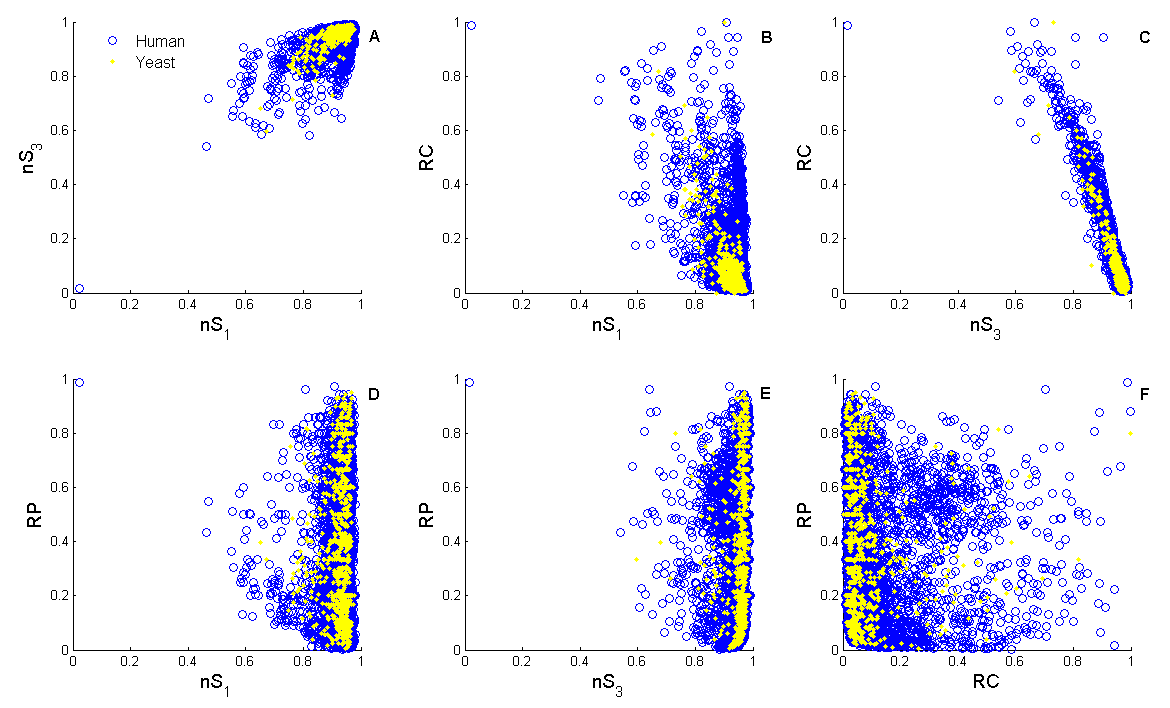

Supplement: Figure S7 — CO measures. Scatter plots of compositional bias entropy measures of single amino-acids and of triplets of amino-acids, the relative coverage RC, and the relative periodicity RP, for human (blue) and yeast (yellow). A) The relationship among the normalized entropies, nS1 and nS 3 (Pearson correlation is 0.61 in human and 0.76 in yeast). B) The relationship between nS1 and RC. C) RC is highly correlated with nS3 (Pearson correlation of 0.93 in human and 0.94 in yeast). D–F) the relationship between RP andnS1, nS3, and RC, respectively. Correlation between RP and all these measures are very weak, indicating that RP provides independent information. (TIF) [file pcbi.1003346.s007.tif]

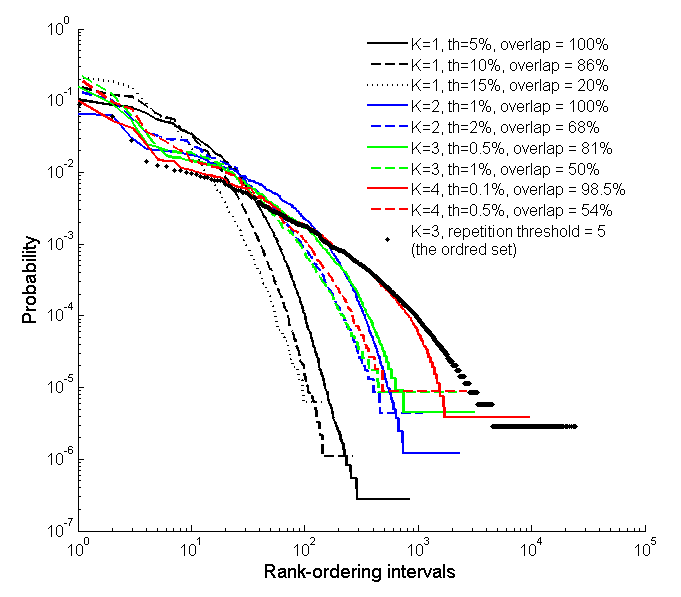

Supplement: Figure S8 — Sensitivity tests based on the rank-ordering interval distributions. Sensitivity test of human rank-ordering interval distributions for different k-mers (k) and repetition thresholds (th). Threshold is defined in terms of the ratio of the number of repetitions by the protein length. The proteins that are found to have k-mers that pass the threshold define a new CO set, which is compared with our original CO (defined by 5 repeats and k = 3). The percentage of overlap between the two sets is shown. Note that k = 4, th = 0.1%and the regular FT definition k = 3, th = 5 provide approximately the same CO proteins, and their interval distributions are very similar: they practically overlap, except for their tails. (TIF) [file pcbi.1003346.s008.tif]

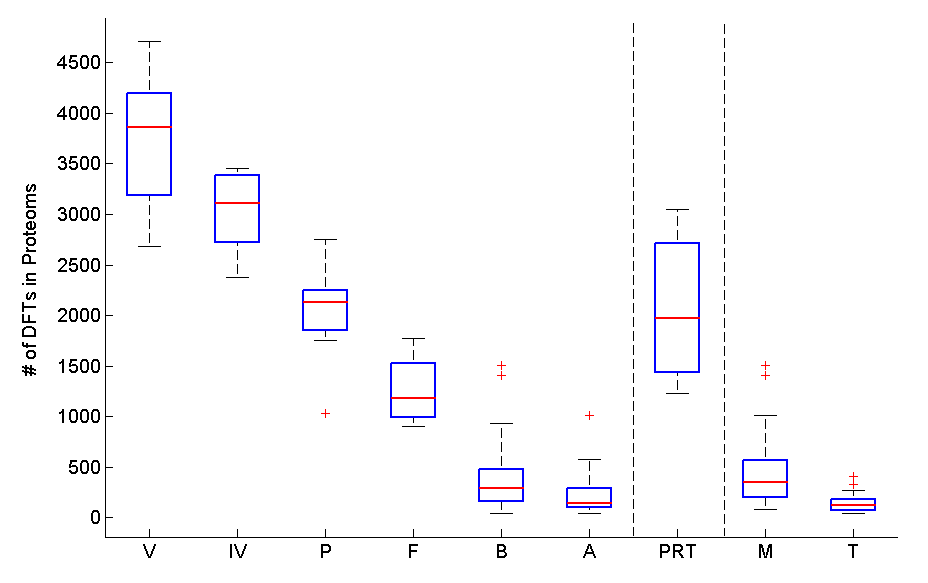

Supplement: Figure S9 — DFT hierarchy evaluated with restrictive FTs. Similar box plots of DFT counts across the tree-of-life to the one presented in figure 5, but using the restrictive FT definition. Each box delineates lower quartile, median and upper quartile values. Most extreme values (whiskers) are within 1.5 times the inter-quartile range from the ends of the box. Outliers are also displayed. Prokaryotes are displayed twice. First grouped according to bacteria and archaea, and secondly as mesophiles and thermophiles. P-values according to non-parametric two-sample Kolmogorov-Smirnov test are 2.5×10^−2^ (V-IV), 3.6×10^−3^(IV-P), 9.8×10^−3^ (P-F), 7.86×10^−6^ (F-B), 2.3×10^−2^ (B-A) and 1.38×10^−4^ (M-T). Protista species show large variability and cannot be distinguished from Plantae or Fungi by the DFT measure. (TIF) [file pcbi.1003346.s009.tif]

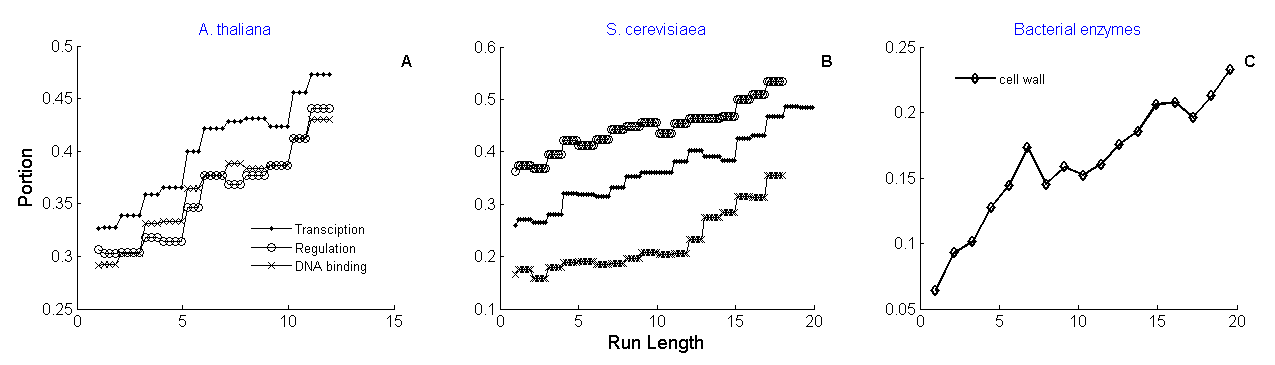

Supplement: Figure S10 — Dependence on run length. Certain GO terms that depend on runs length as measured by the number of repetitions at MFI = 1. Run length is associated with DNA-binding, regulation and transcription in A. thaliana (A) and S. cerevisiae (B). Bacterial enzymes show similar behavior of cell wall proteins (C). (TIF) [file pcbi.1003346.s010.tif]

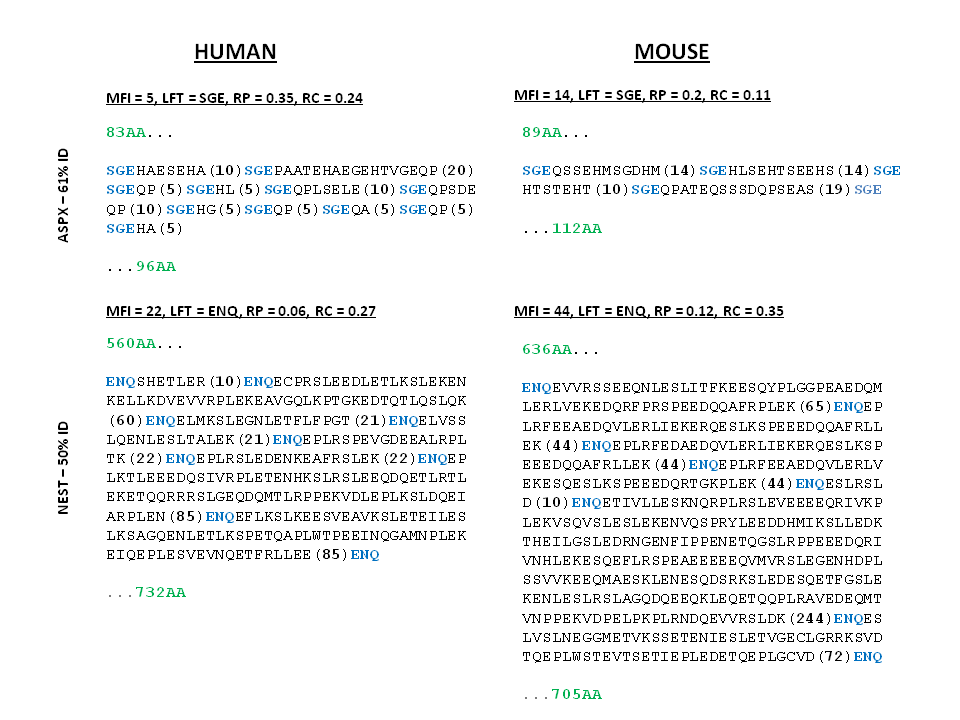

Supplement: Figure S11 — Interval shift in mouse. Examples of orthologous sequences in human and mouse with low sequence similarity. Numbers followed by AA (green) indicate the numbers of amino-acid before and after the repetitive section. Within the repetitive section the leading FTs at MFI are highlighted (blue) and the number of amino-acids between recurrences of FTs is given in ( ) for visual convenience. These numbers allow for easy observation of the existence of “harmonics”. (TIF) [file pcbi.1003346.s011.tif]

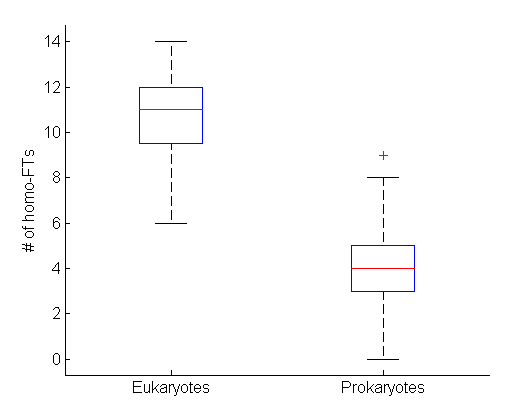

Supplement: Figure S13 — Comparison of runs in eukaryotes and prokaryotes. Comparison of runs in eukaryotes and prokaryotes carried out for the leading 45 FTs, covering all species. The threshold of 45 was chosen because it is the minimal DFT count among all species. Plotted are the numbers of FTs composed of a single amino-acid, representing runs. The Box-plot demonstrates the significant abundance of runs in eukaryotes (P-value = 1.067×10^−16^, non-parametric two-sample kolmogorov-smirnov test). (TIF) [file pcbi.1003346.s013.tif]

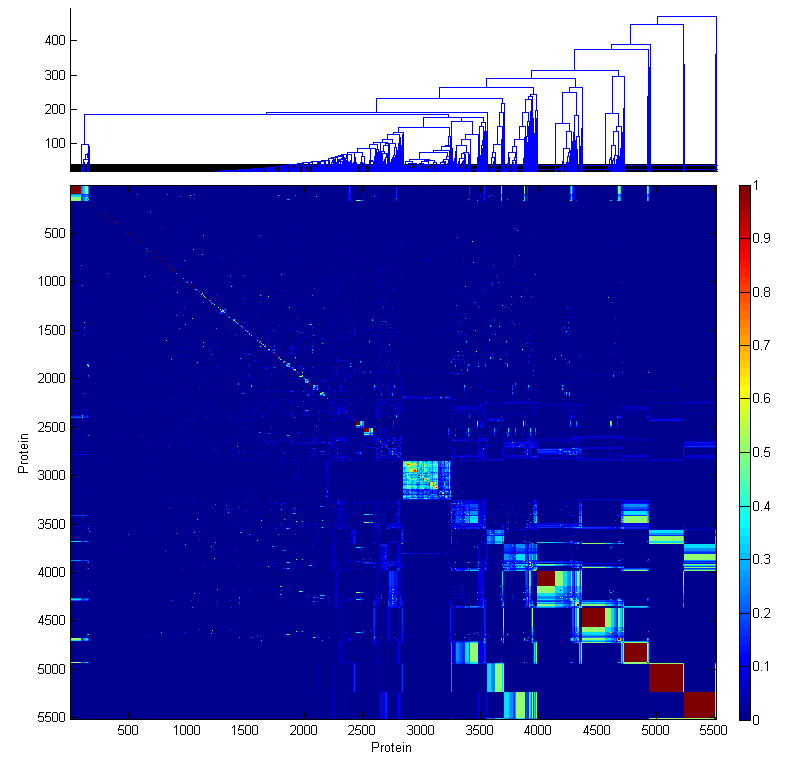

Supplement: Figure S14 — Clustergram of DFT correlation among human proteins. Hierarchical clustering based on the matrix CIJ of 5511 human CO proteins is shown at the top. Heatmap of CIJ is shown at the bottom, revealing about 10 large clusters in addition to several small ones. The big group in the middle, around index 3000, contains mostly ZF proteins. (TIF) [file pcbi.1003346.s014.tif]

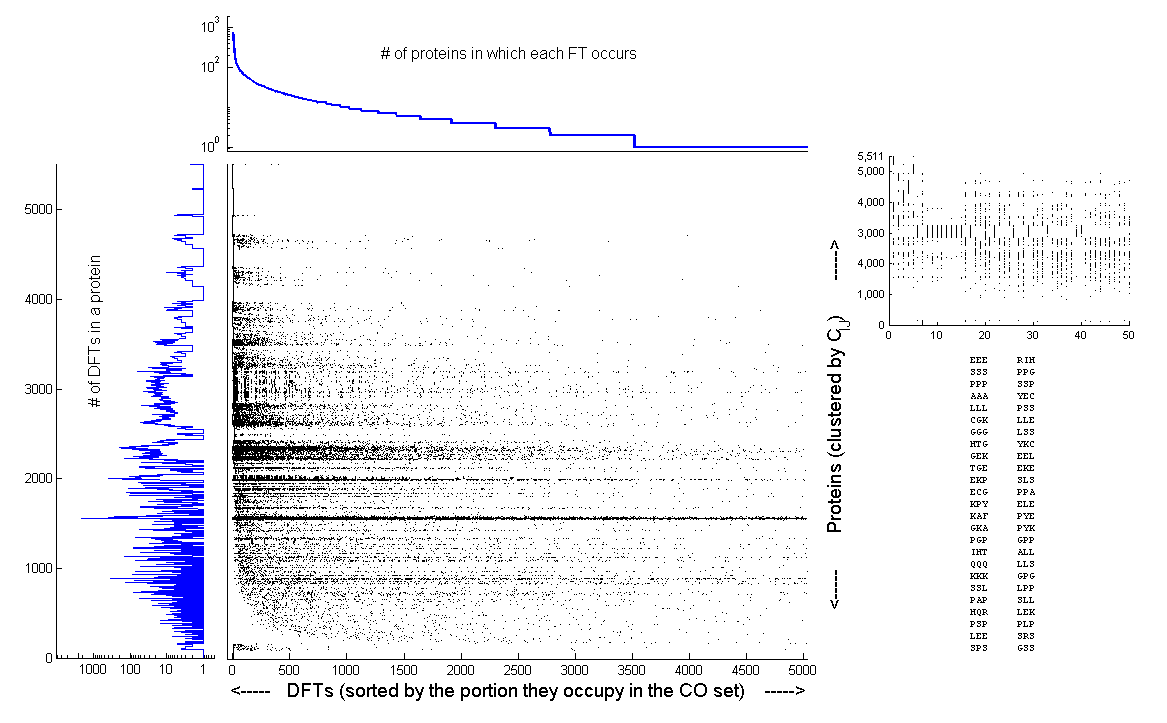

Supplement: Figure S15 — Summary of DFT presence in the human proteome. DFT are ordered according to their abundance in the human Swiss-Prot proteome (x-axis, main panel) and proteins are ordered according to the classification of the clustergram in figure S14 (y-axis, main panel). The abundance of each FT is shown in the upper panel (blue) and the number of DFTs in each protein is shown on the left panel (blue). On the right, zoom in into the 50 most prevalent FTs showing the co-occurrences in groups of proteins. Note that their rank is slightly different from Table 6, which is based on NCBI-RefSeq. (TIF) [file pcbi.1003346.s015.tif]

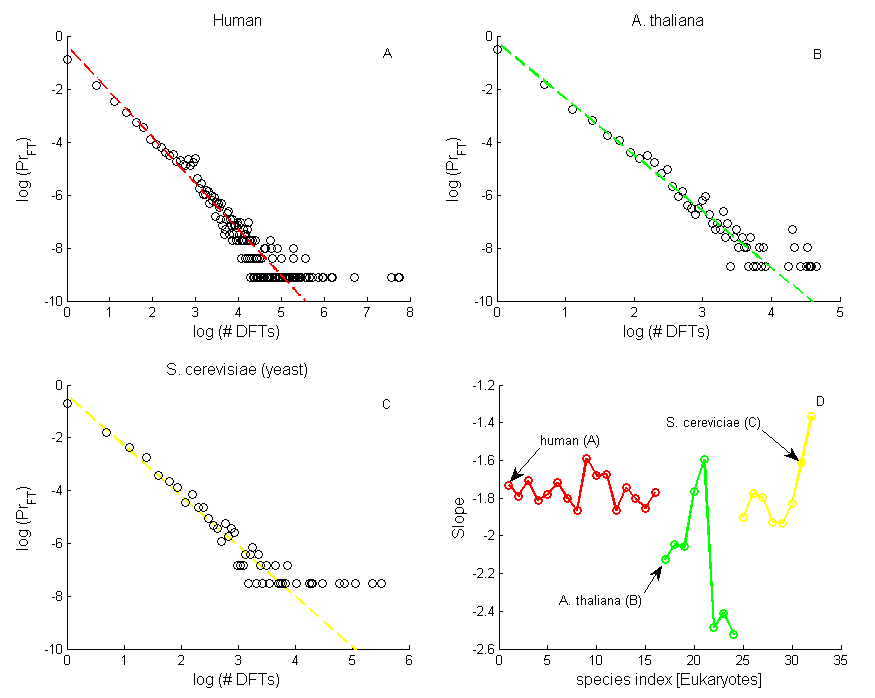

Supplement: Figure S16 — Linear fits of the DFT probability distribution functions in eukaryotes. Linear fits of the DFT probability distribution functions are shown for human (A), A. thaliana (B) and S. cerevisiae (C). A–C) black circles are the data points and colored dashed lines are the fits over range 0–4 of the x-axis. P-values are 2.5×10^−36^, 2.7×10^−32^, 3×10^−24^ in human, A. thaliana and S. cerevisiae, respectively. All eukaryote data have corresponding power-law fits with P-values smaller than 10−17. D) The slopes (i.e. the power law exponents) of all eukaryotes we have analyzed. The cases in A–C are indicated by arrows. (TIF) [file pcbi.1003346.s016.tif]

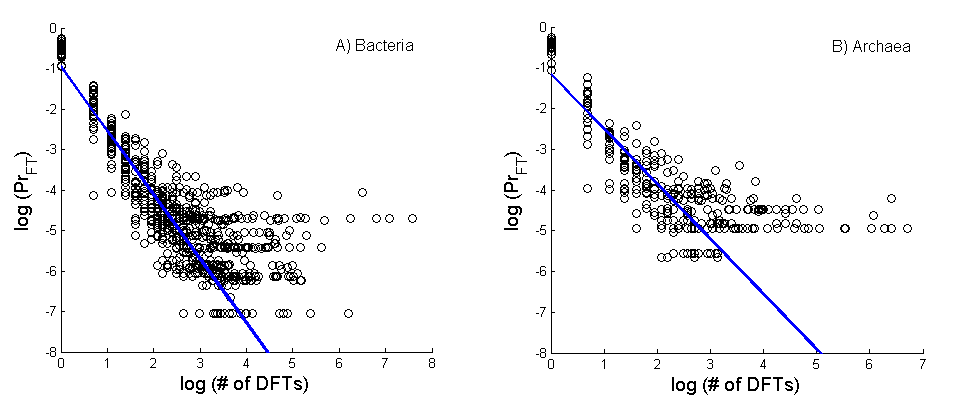

Supplement: Figure S17 — Linear fits of the DFT probability distribution functions in prokaryotes. A) Individual DFT probability distribution function of all bacterial species (n = 36) superimposed (black circles). Linear fit, for the range 0–3, was applied to all data points (P-value∼10−177, slope = −1.6). B) Same procedure applied for all archaea species (n = 19). Linear fit with P-value∼10−65 and slope = −1.35. (TIF) [file pcbi.1003346.s017.tif]

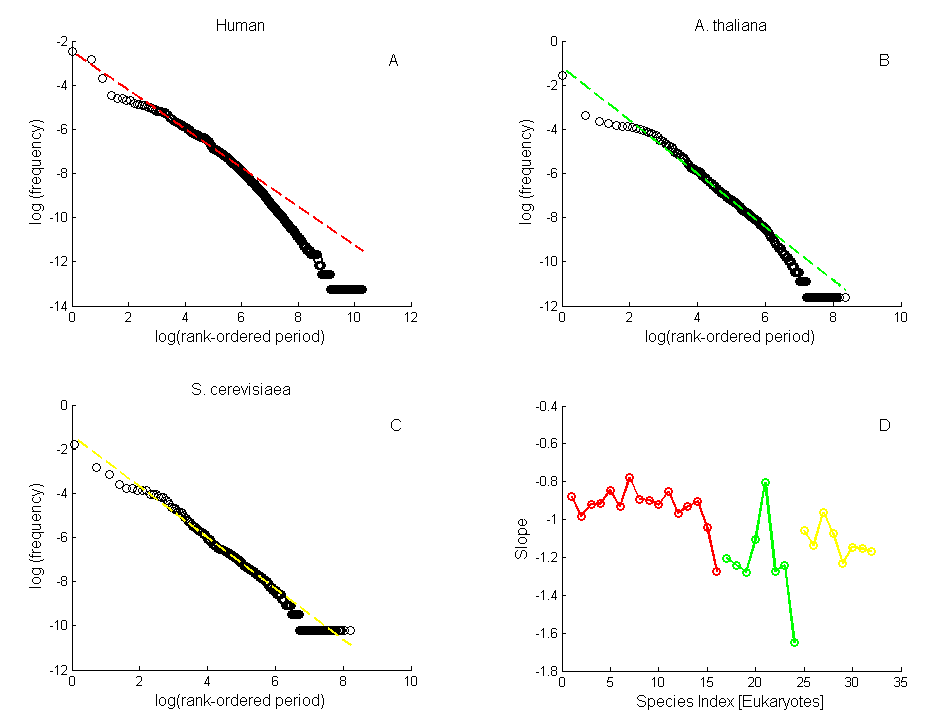

Supplement: Figure S18 — Linear fits of the rank-ordered probability distribution functions in eukaryotes. rank-ordered distribution function (black circles) and the corresponding linear fits over the range 0–6 are shown for human (A), A. thaliana (B) and S. cerevisiae (C) as colored dashed lines. Applying such fits to all eukaryotes we find that the power-law exponents are close to −1 (D), displaying a universal behavior that is close to the Zipf law. P-values in all cases are practically 0. (TIF) [file pcbi.1003346.s018.tif]

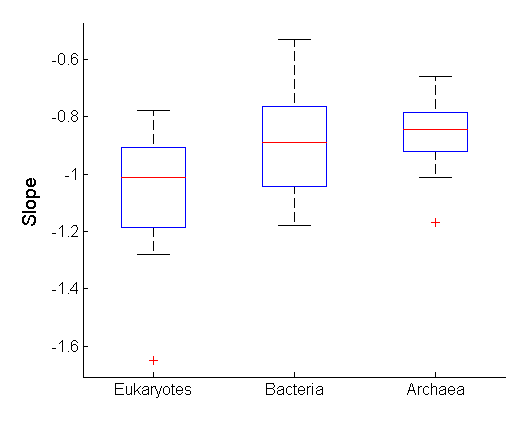

Supplement: Figure S19 — Slopes of the rank-ordered probability distribution functions. Slopes of all individual interval probability distribution functions as obtained by a linear fit. P-values among all species were lower than 10−39. (TIF) [file pcbi.1003346.s019.tif]

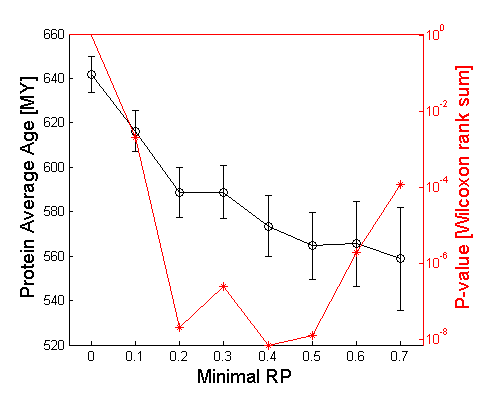

Supplement: Figure S20 — Protein age vs RP. The average age of proteins (black) is shown versus elevated RP. Error bars on the mean age are also shown. The statistical significance of the difference between the age distribution for a given RP threshold and the age distribution of the entire CO set was estimated according to Wilcoxon rank-sum test (red). (TIF) [file pcbi.1003346.s020.tif]
