# Supplementary material for: Systematic Analysis of Compositional Order of Proteins Reveals New Characteristics of Biological Functions and a Universal Correlate of Macroevolution
Source: PLoS Comput Biol. 2013 Nov 21;9(11):e1003346. doi: 10.1371/journal.pcbi.1003346 (PMC3836704; doi:10.1371/journal.pcbi.1003346)
Supplement: Table S2 — Characteristics of the 10 longest human proteins. Characteristics of the 10 longest human proteins, displaying their length, assigned DFT counts according to the regular and restrictive definitions, and error estimates on the latter, given in ‘()’, for the uniform and unigram random models respectively. In the unigram model the inhomogeneous drop with decreasing length is due to statistical fluctuations. Last column shows that amendment due to periodicity considerations (see figure S5) increases considerably the number of validated FTs. (DOCX) [file pcbi.1003346.s022.docx]

| **Name** | **Length** | **Regular DFT** | **Restrictive DFT (Uniform, Unigram)** | **+ FT selection**  **by intervals**  (Threshold set by  Unigram) |
| --- | --- | --- | --- | --- |
| TITIN_HUMAN | 34350 | 2375 | 413 (2, 59) | 1642 |
| MUC16_HUMAN | 22152 | 1032 | 530 (2, 38) | 927 |
| SYNE1_HUMAN | 8797 | 389 | 98 (1, 15) | 117 |
| OBSCN_HUMAN | 7968 | 397 | 171 (1, 13) | 265 |
| DYST_HUMAN | 7570 | 292 | 69 (1, 11) | 159 |
| MACF1_HUMAN | 7388 | 288 | 70 (1, 12) | 166 |
| FSIP2_HUMAN | 6907 | 238 | 59 (0, 11) | 106 |
| SYNE2_HUMAN | 6885 | 262 | 75 (0, 10) | 133 |
| NEBU_HUMAN | 6669 | 351 | 238 (0, 11) | 342 |
| GPR98_HUMAN | 6306 | 207 | 46 (0, 10) | 84 |

**Table S2: Characteristics of the 10 longest human proteins**

Characteristics of the 10 longest human proteins, displaying their length, assigned DFT counts according to the ***regular*** and ***restrictive*** definitions, and error estimates on the latter, given in ‘()’, for the uniform and unigram random models respectively. In the unigram model the inhomogeneous drop with decreasing length is due to statistical fluctuations. Last column shows that amendment due to periodicity considerations (see figure S5) increases considerably the number of validated FTs.
