# Supplementary material for: Systematic Analysis of Compositional Order of Proteins Reveals New Characteristics of Biological Functions and a Universal Correlate of Macroevolution
Source: PLoS Comput Biol. 2013 Nov 21;9(11):e1003346. doi: 10.1371/journal.pcbi.1003346 (PMC3836704; doi:10.1371/journal.pcbi.1003346)
Supplement: Table S3 — Mutation and selection in cell wall proteins in yeast by DNA analysis. Analysis of repetitive motifs in cell wall proteins of S. cervisiaea. First and second columns show the name, function and starting location of the repetitive section. Third and fourth columns show the sequences at the protein and DNA levels respectively. Motifs are divided into identical sections forming groups which are colored with gray (group 1), blue (group 2) and white (group 3). The first group is taken as a reference motif and the remaining groups are compared to it in the following way: Yellow colored letters indicate mutations that cause the generation of a distinct motif, i.e. amino-acids different from group 1. Red colored letters indicate synonymous mutations within each motif group, thus do not change the motif composition at the amino-acid level, indicating that some amino-acids are protected by selection. (DOCX) [file pcbi.1003346.s023.docx]

| Gene | Repeat Start Location | Motifs | DNA of Motifs |
| --- | --- | --- | --- |
| TIR4  (cell wall) | 138 | 1. SSVAPSSSEVVS  2. SSVAPSSSEVVS  3. SSVAPSSSEVVS  4. SSVASSSSEVAS  5. SSVAPSSSEVVS  6. SSVASSSSEVAS  7. SSVAPSSSEVVS  8. SSVAPSSSEVVS  9. SSVASSSSEVAS  10.SSVAPSSSEVVS | 1. TCTTCTGTTGCACCATCCTCAAGTGAAGTTGTCAGC  2. TCTTCCGTTGCACCATCCTCAAGTGAAGTTGTCAGC  3. TCTTCCGTTGCACCATCCTCAAGTGAAGTTGTCAGC  4. TCTTCCGTTGCTTCATCCTCAAGTGAAGTTGCCAGC  5. TCCTCTGTTGCGCCATCCTCAAGTGAAGTTGTCAGC  6. TCTTCCGTTGCTTCATCCTCAAGTGAAGTTGCCAGC  7. TCCTCTGTTGCGCCATCCTCAAGTGAAGTTGTCAGC  8. TCTTCCGTTGCACCATCCTCAAGTGAAGTTGTCAGC  9. TCTTCCGTTGCTTCATCCTCAAGTGAAGTTGCCAGC  10.TCCTCTGTTGCGCCATCCTCAAGTGAAGTTGTCAGC |
| AGA1  (cell wall) | 189 | 1.TSTSPSS  2. TSTSPSS  3. TSTSSSS  4. TSTSSSS  5. TSTSSSS  6. TSTSPSS  7. TSTSSSL  8. TSTSSSS  9. TSTSQSS  10.TSTSSSS  11.TSTSPSS  12.TSTSSSS  13.TSTSPSS | 1. ACATCTACATCTCCAAGCTCT  2. ACATCTACATCTCCAAGCTCT  3. ACATCTACCTCATCAAGTTCG  4. ACATCTACCTCATCAAGTTCG  5. ACATCTACCTCATCAAGTTCG  6. ACATCTACATCTCCAAGTTCG  7. ACATCCACATCTTCAAGTTTG  8. ACATCCACATCTTCAAGTTCT  9. ACATCTACATCCCAAAGTTCT  10.ACATCTACCTCATCAAGTTCG  11.ACATCTACATCTCCAAGCTCT  12.ACATCTACCTCATCAAGTTCA  13.ACATCTACATCTCCAAGTTCT |

**Table S3: Mutation and selection in cell wall proteins in yeast by DNA analysis**

Analysis of repetitive motifs in cell wall proteins of *S. cervisiaea*. First and second columns show the name, function and starting location of the repetitive section. Third and fourth columns show the sequences at the protein and DNA levels respectively. Motifs are divided into identical sections forming groups which are colored with gray (group 1), blue (group 2) and white (group 3). The first group is taken as a reference motif and the remaining groups are compared to it in the following way: Yellow colored letters indicate mutations that cause the generation of a distinct motif, i.e. amino-acids different from group 1. Red colored letters indicate synonymous mutations within each motif group, thus do not change the motif composition at the amino-acid level, indicating that some amino-acids are protected by selection.
