# Supplementary material for: Systematic Analysis of Compositional Order of Proteins Reveals New Characteristics of Biological Functions and a Universal Correlate of Macroevolution
Source: PLoS Comput Biol. 2013 Nov 21;9(11):e1003346. doi: 10.1371/journal.pcbi.1003346 (PMC3836704; doi:10.1371/journal.pcbi.1003346)
Supplement: Table S4 — Comparison between DFT counts and Neu values. Listed are all eukaryotes for which we could retrieve the Neu measure from Lynch & Conery 2003. Left: species are ordered according to DFT counts in ascending order. Right: Same species ordered according to Neu in descending order. Both measures provide an hierarchical ordering of major clades. They also significantly correlate (correlation coefficient = −0.6, P-value = 0.012). Clade notations are the same as in figure 5 of the main text. (DOCX) [file pcbi.1003346.s024.docx]

| **Species** | **DFT** | **Clade** | **Species** | **Ne *x* u** | **Clade** |
| --- | --- | --- | --- | --- | --- |
| Zea mays | 1037 | P | Cryptococcus neoformans | 0.02526 | F |
| Cryptococcus neoformans | 1050 | F | Saccharomyces cerevisiae | 0.02294 | F |
| Saccharomyces cerevisiae | 1077 | F | Dictyostelium discoideum | 0.01825 | PRT |
| Neurospora crassa | 1780 | F | Neurospora crassa | 0.0113 | F |
| Oryza sativa | 1846 | P | Toxoplasma gondii | 0.00688 | PRT |
| Arabidopsis thaliana | 2262 | P | Leishmania major | 0.00521 | PRT |
| Leishmania major | 2319 | PRT | Drosophila melanogaster | 0.00374 | IV |
| Toxoplasma gondii | 2840 | PRT | Zea mays | 0.0033 | P |
| Dictyostelium discoideum | 2990 | PRT | Caenorhabditis elegans | 0.00328 | IV |
| Anopheles gambiae | 3518 | IV | Arabidopsis thaliana | 0.00323 | P |
| Caenorhabditis elegans | 3722 | IV | Ciona intestinalis | 0.00305 | IV |
| Fugu rubripes | 3746 | IV | Anopheles gambiae | 0.00298 | IV |
| Ciona intestinalis | 4019 | IV | Strongylocentrotus purpuratus | 0.0023 | IV |
| Drosophila melanogaster | 4146 | IV | Fugu rubripes | 0.00101 | IV |
| Mus musculus | 4873 | V | Oryza sativa | 0.00077 | P |
| Homo sapiens | 5076 | V | Homo sapiens | 0.00031 | V |
| Strongylocentrotus purpuratus | 5477 | IV | Mus musculus | 0.00027 | V |

**Table S4: Comparison between DFT counts and N_e_u values**

Listed are all eukaryotes for which we could retrieve the N_e_u measure from Lynch & Conery (2003). Left: species are ordered according to DFT counts in ascending order. Right: Same species ordered according to N_e_u in descending order. Both measures provide an hierarchical ordering of major clades. They also significantly correlate (correlation coefficient = -0.6, *P*-value = 0.012). Clade notations are the same as in figure 5 of the main text.
