# Supplementary material for: Systematic Analysis of Compositional Order of Proteins Reveals New Characteristics of Biological Functions and a Universal Correlate of Macroevolution
Source: PLoS Comput Biol. 2013 Nov 21;9(11):e1003346. doi: 10.1371/journal.pcbi.1003346 (PMC3836704; doi:10.1371/journal.pcbi.1003346)
Supplement: Text S1 — Supporting analysis. Description of all the supporting analyses (13 sections) that are mentioned in the main text. (DOCX) [file pcbi.1003346.s026.docx]

**Supporting Information – Text S1**

**Section 1: Analyses of random models of protein sequences: establishing the statistical significance of Frequent Triplet (FT) definitions**

In this section we provide analyses of random models which will allow us to establish *P*-value limits on the validity of FT assignments for proteins. Using a uniform random model of 20 amino-acids with equal probability we derived in Methods the following theoretical expectation value, *E*, for observation of different frequent triplets (DFTs) on a protein of length *L*:

(1) *E = 8000* $\sum_{i=n}^{L} \frac{L!}{i !\left( L-i \right)!}p^{i}{(1-p)}^{L- i}$

which is based on the binomial probability function with *p=1/8000*, signifying the probability of observing a single amino acid triplet anywhere along the protein sequence. Our ***regular*** FT definition requires *n=5*. Finding such an FT on a random sequence may be used as a measure for refuting an FT finding on a real protein, i.e. it is a measure of the possible error in an FT search. Hence we define our *P*-value for a single FT as E/8000. The expectation values, and the derived *P*-values, depend on *L*. Whereas they are very small for short proteins, they become large for very long proteins. Although such long proteins are exceptional, we introduce a ***restrictive*** FT definition, requiring the observation of at least 5 repetitions to occur within a window of length *M* amino-acids anywhere on the protein. In other words, a ***regular*** FT is disqualified from the restrictive set if no 5 consecutive occurrences appeared within at least one section of length M.

Searching for regular FTs, we find that the maximal length of human proteins that are not CO (defined as NO) is about 2000 amino-acids (Fig. S1). Therefore, and because *E* vanishes for L < 2000 (methods, Fig. 10), we chose for our analysis *M = 2000* as the restrictive working point. At *M = 2000*, this ***restrictive*** definition decreases the expected values and the *P*-values considerably for the same underlying uniform background model (Fig. S2a,b), such that even for the largest protein the *P*-value < 10e-3.

Next we turn to a background random model based on the unigram of amino acid frequencies observed on human proteins. We chose human for this purpose since it has the largest number of CO proteins among all the species that we have analyzed. Simulation results show an increase of the expectation values and *P*-values for the ***regular*** (Fig. S2c) and ***restrictive*** (Fig. S2d) FT definitions. They are somewhat larger than in the previous random model because the unigram distribution leads to a decrease in the effective number of amino acids: A fit to *E* in Fig. S2c, within the domain of *L <8000,* can be obtained using Eq. 1 with an effective alphabet of 16 letters.

**Section 2: Detailed analyses of CO parameters in the human proteome and random models with regular and various restrictive FT definitions**

Here we study the sensitivity of CO key parameters in the human proteome to the choice of the constraint *M*, used in the ***restrictive*** FT definition, and we compare the results to the random models described in section 1 (Fig. S3). Using Swiss-Prot human data, we generated artificial random proteomes (10 uniform and 10 unigram models) that have identical length distribution to the human proteome. FT search was conducted on the real human proteome as well as on the two random models. First, we analyzed how the size of the human CO set, *N_CO_*, changes with *M* (Fig. S3a). It shows that *N_CO_* is stable for *M* > 1000. Moreover, since *M* affects the number of identified FTs in a single protein that has length *L > M*, we explored the change, Δ, in this number between *M = Inf* and any of the *M =* [500, 3500] (Fig. S3b)*.* Already at *M* =500, we find that only a small fraction (*< 5%*) of proteins show a difference of *Δ > 1%* of the number of FTs identified at *M = Inf*.

Comparing the human to the two random models for all choices of M, we find that in the uniform random model the number of FT-containing proteins is always lower than 3% of the human *N_CO_* (Fig. S3c), and in the unigram model it is always lower than 15% (Fig. S3e). The number of different frequent triplets (DFTs) in the entire proteome, our global variability-measure of proteomic FT-richness, is also much higher in the human compared with any random model and for any choice of *M* (Fig. S3d,f). Thus the signal to noise ratio is large for all *M*.

Moreover, unlike in human, the number of DFTs identified in the uniform random model depends crucially on two very long proteins, random analogs of TITIN and MUC16 with length of 34350 and 22152, respectively. To test the actual contribution of each individual protein to the proteomic DFT count, we also re-assessed the number of DFTs in the proteomes of proteins, sorted by their number of FT-assignments, if the individual protein had been removed from the proteome (Fig. S4). This was done with both the regular (Fig. S4a) and restrictive definition (M = 2000, Fig. S4b).

In fact, we find in human just 11 proteins with length *L* > 6000 amino acids, 32 proteins with *L >* 5000 and 80 proteins with *L >* 4000. Only TITIN and MUC16 extend to lengths where regular FT assignments may become problematic. For the two largest proteins, the restrictive FT assignments are much smaller than the regular ones. Next we will argue that the restrictive definition is, in fact, much too restrictive. The reason is that it is based on counts of FTs within some window. A different argument establishing non-random behavior is that of periodicity. If most of the DFTs in the long protein participate in periodic structures, they may be regarded as bona-fide members of compositional order.

In Fig. S5 we study in detail the periodic structures of the two long proteins TITIN and MUC16. The non-random high-order structures that exist in these proteins are significant and very evident (Fig. S5 *a* and *d*). Both contain many repetitive elements of very large intervals (as well as small intervals). Although many of these intervals are captured also by restrictive FTs, the number of DFTs indentified in TITIN increases significantly once these high-order structures are taken into account as evidence for the non-random character of their corresponding FTs. Assuming that a regular FT is not disqualified if one of its intervals is significant, i.e. appears much more than random (as set by the threshold in Fig. S5), then the number of DFTs in TITIN becomes 1639. All except 3 of the 413 DFTs found by the restrictive definition (see Table S2) are contained in this list, hence we arrive at a total count of 1642 DFTs. This number is much closer to the value obtained by the regular FT assignment (2375) than by the restrictive FT assignment (413). MUCIN 16 displays similar behavior. Here we find 922 validated FTs by the interval argument + additional 5 found exclusively by the restrictive definition, leading to a total of 927. This is again much closer to the value 1032 of the regular FT definition than 530 of the restrictive FT definition. The situation for all human proteins longer than 6000 amino-acids is summarized in Table S2. We conclude therefore that the restrictive definition provides a lower bound to the DFT count in a protein, and many more FTs are validated by the periodicity argument. The numbers vary according to the length of the protein, but the order of magnitude is set by the regular DFT count. Hence we chose to use the regular FT definition throughout this paper, remembering however that for studies of very long proteins special care has to be taken when selecting the FTs that can be clearly validated as indicators of compositional order.

We emphasize that the problem of FT-assignment in long proteins is less acute at the proteome level, i.e., for the estimation of proteomic DFT counts, as also demonstrated in Fig. S4. This is because when the total proteome is concerned, even the largest proteins contribute a small fraction of the total DFT count since many of the DFTs are observed to occur on other proteins. Hence the total number of DFTs on the proteome is not diminished as much as one may have expected from the pure random model.

The conclusion from sections 1 and 2 is that the ***regular*** definition is sufficient and contains more biologically relevant information. Its sole disadvantage is in introducing large numbers of FT assignments in very long proteins, which we discussed and analyzed separately.

**Section 3: CO set size and length distribution across the tree-of-life**

Following the boxplot presented in the main text (Fig. 5), we show in this section that other types of measurements such as the number of genes in the proteome (Fig. S6a), the number of CO proteins (Fig. S6b), and their length averages (Fig. S6 c and d, respectively) do not show any clear delineation of the main taxonomic groups that comes close to the result of the DFT count. Note that the average protein length of CO proteins is higher than the average length of all proteins in an organism. It tends to be flat across the tree-of-life, further emphasizing that CO measures do not need any normalization factors that depend on protein length.

**Section 4: Analysis of the relationship between CO measures**

The CO sets of human (blue) and yeast (yellow) are measured and compared in Fig. S7. We measured normalized single amino-acid entropy, *nS_1_*, and normalized triplet entropy *nS_3_*, and compared them to our new CO measures (see Methods), the relative coverage (RC) of FTs and the relative periodicity (RP). The results show that RC is a good measure of regularity since it strongly anti-correlates with entropy. RP does not have any significant correlation with neither RC nor with any of the entropies. Evaluating the Pearson correlation coefficients, ρ, for all 94 species, we find that correlation of RC with nS_3_ is very strong (ρ = -0.925 ± 0.02) and that the correlation of RC with RP is weak (ρ = -0.07 ± 0.13), indicating that they contain independent information.

**Section 5: Sensitivity analysis of CO sets - interval distribution for different *k*-mers, and alternative repeat threshold definitions.**

Here we demonstrate (Fig. S8) that the composition of the CO set is stable provided that one searches for sufficiently rare events (i.e. *k*-mers with *k ≥ 3*). We studied changes in the properties of CO sets and in the interval distributions for alternative choices of FT definitions with *k=1-4*. In addition, we studied different alternative definitions of the repeat threshold *n*. Specifically, we allowed the minimal number of repeats to depend on the length of the protein. In Fig. S8 we define the minimal number for repeats, above which a triplet is assigned as an FT, in terms of a certain percentage of the protein’s length.

For any *k*, it is possible to find a relative-to-length threshold that would lead to a CO set that overlaps strongly with the CO defined by our regular FT definition (i.e., with *k=3,* and fixed threshold of minimum 5 repeats*)*. Thus, for *k=4* (*th = 0.1%*) the overlap is larger than 98%. But only for *k ≥ 3,* also the interval distributions largely overlap, except in the tails.

Power-law tails are obtained only for large *k*-values. Both *k =3* and *4*, whether with fixed threshold of occurrences or relative-to-length threshold, provided the heaviest tail. This we view as a definite advantage, indicating that they are appropriate markers of repetitive motifs. To the extent that CO and its associated periodic structures are created by an evolutionary mechanism, we wish it to be somewhat stable against mutational changes. The choices of *k=1* or *2* are highly sensitive to mutations, which lead to fast decrease of order in the intervals. This provides an a-posteriori justification for our analysis being based on FTs.

**Section 6: Stability of the DFT kingdom hierarchy pattern**

The hierarchical delineation of the major taxonomic groups presented in Fig. 5 of the main text, has been evaluated by using ***regular*** FTs. Here we demonstrate that the same characteristic features of the hierarchy are obtained if one replaces regular FTs with ***restrictive*** FTs (Fig. S9). The use of the latter leads to a slight quantitative improvement of some *P*-values (for IV-P and F-B) in this hierarchy. We have also performed few other tests, showing that various other changes leave this hierarchy intact:

1) Eliminating long proteins. Excluding all proteins with *L* > 4000 amino-acids one retains the same hierarchical separation with similar *P*-values for the separation between the main taxonomic groups: 2.5x10^-2^ (V-IV), 3.6x10^-3^(IV-P), 9.8x10^-3^ (P-F), 1.6x10^-6^ (F-B), 6.7x10^-3^ (B-A) and 3x10^-4^ (M-T). Even excluding all proteins with *L* > 2000 still provides clear ordering but with increase of some *P*-values: 0.08 (V-IV), 0.03(IV-P), 9.8x10^-3^ (P-F), 1.6x10^-6^ (F-B), 6.7x10^-3^ (B-A) and 8.3x10^-4^ (M-T).Only when limiting ourselves to proteins with *L* < 1000, we observe a deterioration of the *P*-values, but there still retains a clear ordering over the whole tree-of-life.

2) Variable threshold of repeats. We increased the number of minimal repeats required with protein length, keeping an upper bound on the empirical *P*-value < 10e-3. By numerical simulations of thousands of random proteins we conclude that the following scaling of repeat threshold achieves this *P*-value: 5 for L < 2000; 6 for 2000 < L <3000, 7 for 3000 < L < 4000, 8 for 4000 < L < 8000 and 15 for L > 8000. In addition, for long proteins (L > 2000) an amino-acid triplet is classified as an FT also if it has at least 5 occurrences, and at least one interval which appear more than twice. Using this modified FT definitions we obtain similar *P*-values for the separation between taxonomic groups: 2.5x10-2 (V-IV), 3.6x10-3 (IV-P), 1.4x10-3 (P-F), 3.65x10-6 (F-B), 5.8x10e-3 (B-A) and 5x10-5 (M-T).

**Section 7: Comparison of Human *vs* Mouse Orthologous CO proteins**

An outstanding feature of repeat patterns in orthologous CO proteins of human and mouse is the clear tendency for higher harmonics in mouse. In Fig. S11 we present two examples, the proteins **ASPX** and **NEST**. The existence of harmonics is evident from the occurrences of intervals with integer multiples within a single protein. Note that sometimes, presumably due to deletions or insertions, the intervals of an FT deviate from pure harmonics by one amino-acid. This serves as evidence that the repetitive sections are subjected to mutational forces that lead to an increase in the intervals and to the creation of harmonics.

To better quantify this tendency we analyzed this apparent shift in the distribution of intervals between the two species, human (A) and mouse (B), summarized in Fig. S12. We chose CO orthologous genes with large periodic sections (MFI > 1) and low sequence similarity (< 70%), defining a set of 204 proteins. Thus, we excluded from this analysis proteins containing significant runs (MFI = 1) because in many cases runs are distributed in several locations in a protein, and therefore may lead to large biases in the estimation of consecutive intervals. For this set of 204 proteins pairs we perform the following dual test: we identify 2 **“**Flag**”**s, FT_A and FT_B, representing the prefix of the underlying repetitive section in each of the proteins of the two species, respectively. A flag is the FT that has the largest number of occurrences within the MFI of the associated protein, i.e. the leading FT. In some cases FT_A (and MFI_A) may be different from FT_B (and MFI_B); therefore, we search for the two flags (FT_A and FT_B) in both species. For each flag we quantify the extent to which its interval distribution in species A differs from its interval distribution in the homologue protein in species B. The latter is estimated by the difference of the mean interval of the two distributions, Δ. The histograms of Δ, for the 204 proteins, are shown in Fig. S12, for FT_human (left) and for FT_mouse (right). A tendency to higher harmonics will lead to a larger Δ and this will cause a shift in the histogram. A fair estimate of such a shift is the count of proteins with negative *vs* positive Δ. This count-based shift estimate is unbiased with respect to the underlying interval length which may differ significantly among proteins. Fig. S12 demonstrates that there is a significant difference between interval shifts when the human flag (left) and when the mouse flag (right) are searched in both species, indicating that there is a much larger discrepancy in mouse intervals than in human ones. Assuming that the mouse lineage suffers from higher mutation rates, this may explain the larger interval modification observed in mouse.

**Section 8: DFT correlation among human proteins**

In the main text we have presented the correlation between the DFT of two proteomes *I* and *J*, defined as *C_IJ_ = (DFT_I_ ∩ DFT_J_) / (DFT_I_ U DFT_J_)*. Similarly, one may define the correlation *C_IJ_* among proteins, measuring the overlap of FT vocabulary between two proteins *I* and *J*. Estimating *C_IJ_* of human CO proteins, we find that above 50% of them can be classified into distinct clusters, as shown in Fig. S14. Performing functional enrichment of GO annotations using GOrilla (see methods) we find that some clusters are enriched with several processes that are unique to them, while certain biological processes are enriched in few clusters (usually 1 to 3 clusters). The latter include various metabolic processes, notably those responsible for the biosynthesis of macromolecules; anatomical structure; morphological; developmental; and regulation processes. Outstanding processes unique to some clusters are those involved with response to unfolded proteins; actin related functions; and transport related functions, notably those involved in brain function. The largest cluster shown in the middle of Fig. S14 comprises most ZF proteins and is involved in numerous DNA, RNA and protein binding functions.

Relying on the order induced by this clustergram we present in Fig. S15 a compendium of our analysis of human proteins. The y-axis of the main panel orders the proteins according the clustergram and the x-axis represents the different FTs according to their frequency of appearance in CO proteins. The top panel reflects this frequency, and the left panel displays the DFT count of proteins. On the right we display a smaller version of the main panel, restricted to the first 50 FTs (the most prominent ones). A list of these FTs is also provided.

**Section 9: Universality of CO measures**

The distribution of DFT occurrences in the proteome is universal. The histogram of the number of DFT (Fig. 8) can be fits excellently fitted by a power-law in all species. Three examples are shown in Fig. S16 for human (Fig. S16a), *A. thaliana* (Fig. S16b) and *S. cerevisiae* (Fig. S16c). We estimated the fits in all Eukaryotes and found that the power-law coefficient is close to 2 in most of them (Fig. S16d). Although the fit of a single prokaryote species DFT distribution to a power-law is poor, when many prokaryotes are superimposed the fit improves and similar behavior to eukaryotes emerges (Fig. S17).

The rank-ordered interval distribution can be fitted by a power-law as well (Fig. S18), with exponents close to -1 in all kingdoms (Fig. S19). This behavior is reminiscent of the Zipf-law, a hallmark of many dynamic evolving systems, as well as languages where words frequency follows this law. In fact, it should be easy to notice that even very long single proteins such as the TITIN and MUCIN16 (see Fig. S5 *a* and *e,* respectively) show this type of behavior. Recurring intervals of leading FTs can be associated with unit-lengths of motifs. Such motifs are the analogs of words in texts.

**Section 10: DNA Analysis - Evidence of mutation and selection acting on repetitive motifs**

We have analyzed DNA sequences of few selected proteins, taking *S. cerevisiae* as an example (table S3). Repetitive motifs within a single protein can be classified into groups, where within each groups the motifs at the protein level are identical. This shows that some mutations have led to generation of distinct motifs. The latter are indicated by yellow, and may lead to novel FTs, presumably serving a function. Looking at the DNA of the same protein sections, one finds that within a group there may exist several point mutations at the third synonymous site (red letters), such that at the protein level the motif retains its amino-acid composition. This is strong evidence for selection. It is reasonable to assume though, that all distinct groups have originated from a single motif because of their high purity, and composition similarity.

**Section 11: Comparison of DFT counts and the Ne*u measure**

The clear evolutionary trend of DFT counts brings to mind the work of Lunch and Conery [2003] which argued, based on the N_e_u measure (*effective population size* *x* *mutation rate*, based on silent sites), that "the transitions from prokaryotes to unicellular eukaryotes to multicellular eukaryotes are associated with orders-of-magnitude reductions in population size; by magnifying the power of random genetic drift, reduced population size provides a permissive environment for the proliferation of various genomic features that would otherwise be eliminated by purifying selection."

We compared our DFT count with N_e_u and found that they anti-correlate (table S4). Over the eukaryotes, where we could use the data listed in their supplementary material (n = 17), the correlation turns out to be -0.6 (*P*-value = 0.012). We find that the DFT count provides a slightly better hierarchical order over the tested eukaryotes. While the high correlation of DFT counts with N_e_u is an indication of the strong power of drift taking place in FT-aspects of evolution, there exists other evidence for purifying and positive selection (see Discussion).

**Section 12: Protein age versus RP**

Capra et al. (2012) [53] have developed a bioinformatic tool (‘ProteinHistorian’) that allows for estimating the age of proteins based on a given phylogenetic tree. They reported a weak but significant positive correlation (Pearson correlation ~ 0.2) between human proteins evolutionary age and their length. Here we wish to further explore the association of protein age with CO proteins and their CO measures, specifically with RP. Thus, we applied their tool to our human CO set.

The average age of CO proteins (641.8 MY) is only slightly smaller than that of the NO proteins that are not CO (681.4 MY), with P-value of < 10^-5^ (Wilcoxon rank sum test). The median ages are the same. However, when testing for the age of proteins above a certain value of the relative periodicity RP we find a clear and significant decrease in the age of protein with elevated threshold of RP (Fig. S20). This was obtained by calculating the mean age, and the error on the mean, taking data points that correspond to a relative error (σ/μ) < 5%. This is the case up to the minimal threshold of RP = 0.7. Afterwards the errors become huge. Estimating for each point the significance of the difference between the decreased mean ages of the subgroups of proteins to the entire CO set, using Wilcoxon rank sum test, we find that this decrease is highly significant (*P*-values < 10^-4^). These results suggest that high RP is not only correlated with short protein length (Fig. 9), but also with relatively young protein age.

**Section 13: Comparing between Human and Mouse Proteomes with various databases and methods**

In the main text we have analyzed and compared the Swiss-Prot proteomes of human (n=20248) and mouse (n=16513). Swiss-Prot is an expert protein data base which is manually curated, and such is the information it provides on homology. Thus, protein orthologs are identified not solely based on sequence comparisons but also on manual curation of their cellular function.

However, because the mouse proteome in Swiss-Prot is smaller than expected, we compare here between the two proteomes using various other databases and methods. To this end, we have analyzed the non-orthologous genes and proteins using BioMart-Ensembl database with various filters.

Using Ensembl Gene database V.72 we extracted non-orthologous genes and protein coding sequences in human (GRCh37.p11) and in mouse (GRCm38.p1). We used 5 different filters: 1) Known genes with RefSeq protein IDs 2) All genes, i.e., including novel and putative, with RefSeq protein IDs 3) All genes with UniProtKB/TrEMBL accessions 4) All genes with Entrez IDs 5) No filter applied. We then run our CO algorithm and extracted the number of CO protein coding sequences. The results are summarized in Table S5. One finds that the ratio between novel CO human proteins and CO mouse varies between 2 to 5, depending on the method of choice.
