# Supplementary material for: Systematic Analysis of Compositional Order of Proteins Reveals New Characteristics of Biological Functions and a Universal Correlate of Macroevolution
Source: PLoS Comput Biol. 2013 Nov 21;9(11):e1003346. doi: 10.1371/journal.pcbi.1003346 (PMC3836704; doi:10.1371/journal.pcbi.1003346)
Supplement: Text S2 — GOrilla analysis of Human CO proteins. Results of functional enrichments in Human CO proteins using GOrilla web-tool. Enrichments of processes, functions and cellular components are shown for three analyses: (i) comparing the Human CO proteins (‘target’) to all Human proteins (‘background’) (ii) Ranking Human CO proteins by RC (ii) Ranking Human CO proteins by RP. (DOCX) [file pcbi.1003346.s027.docx]

**Supporting Information – Text S1**

Analysis of all human CO protein (n=5511) by GOrilla [Eden et al., 2007], shown for two-unranked test (1), and for lists ranked by RC (2) and RP (3).

**1) Two-unranked test** of 5511 human compositionally ordered proteins as target against the human proteome as background with p-value threshold of 10^-6^ yields:

Process


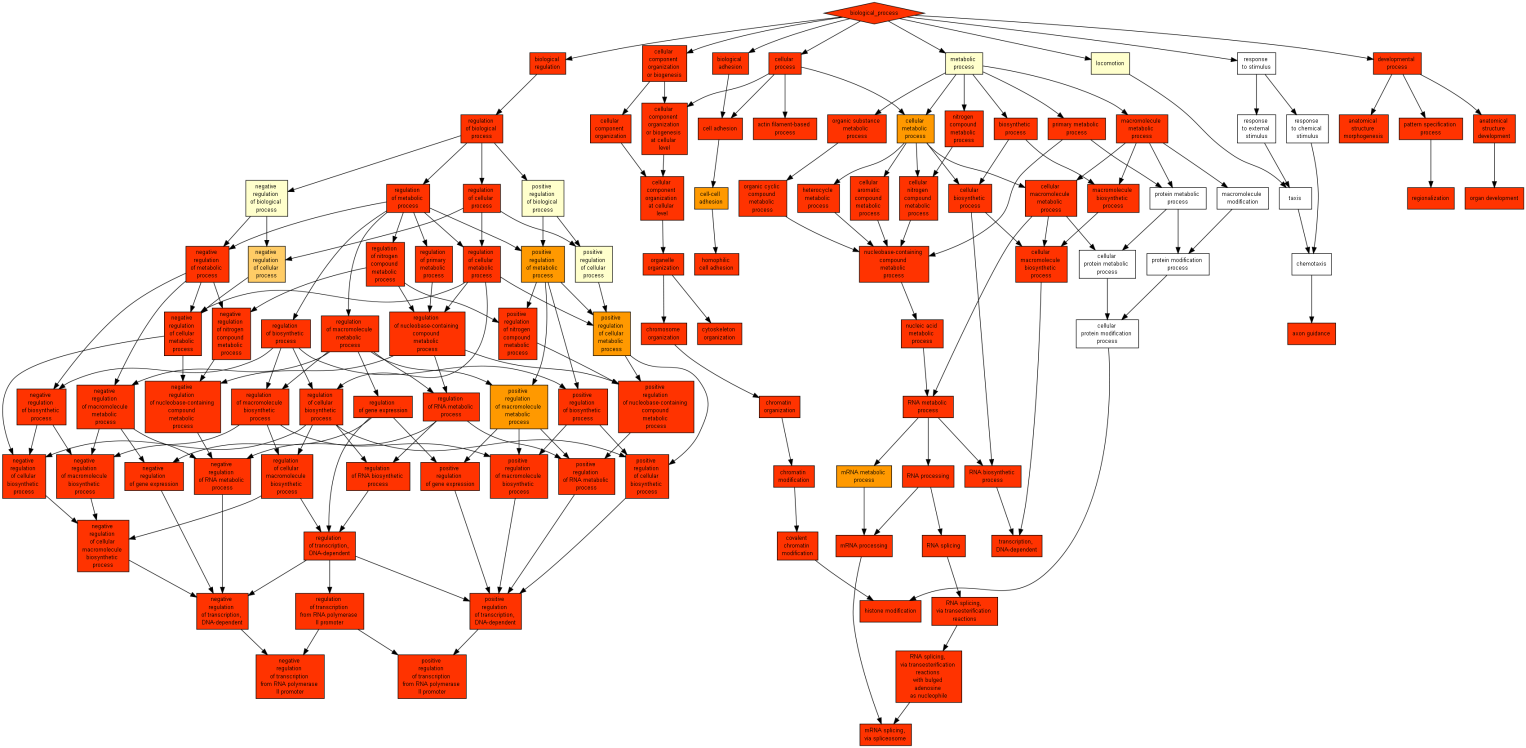


| **GO term** | **Description** | [**P-value**](http://cbl-gorilla.cs.technion.ac.il/GOrilla/6lguiwpv/GOResultsPROCESS.html#p_value_info) | [**FDR q-value**](http://cbl-gorilla.cs.technion.ac.il/GOrilla/6lguiwpv/GOResultsPROCESS.html#fdr_info) | [**Enrichment (N, B, n, b)**](http://cbl-gorilla.cs.technion.ac.il/GOrilla/6lguiwpv/GOResultsPROCESS.html#enrich_info) |
| --- | --- | --- | --- | --- |
| [GO:0006351](http://www.godatabase.org/cgi-bin/amigo/go.cgi?query=GO:0006351&view=details) | transcription, DNA-dependent | 1.05E-153 | 1.17E-149 | 1.90 (17741,2116,5033,1140) |
| [GO:0016070](http://www.godatabase.org/cgi-bin/amigo/go.cgi?query=GO:0016070&view=details) | RNA metabolic process | 2.38E-140 | 1.32E-136 | 1.70 (17741,2951,5033,1423) |
| [GO:0032774](http://www.godatabase.org/cgi-bin/amigo/go.cgi?query=GO:0032774&view=details) | RNA biosynthetic process | 5.8E-140 | 2.14E-136 | 1.81 (17741,2308,5033,1187) |
| [GO:0090304](http://www.godatabase.org/cgi-bin/amigo/go.cgi?query=GO:0090304&view=details) | nucleic acid metabolic process | 1.06E-129 | 2.94E-126 | 1.61 (17741,3405,5033,1558) |
| [GO:0051252](http://www.godatabase.org/cgi-bin/amigo/go.cgi?query=GO:0051252&view=details) | regulation of RNA metabolic process | 3.39E-126 | 7.5E-123 | 1.68 (17741,2851,5033,1356) |
| [GO:0006355](http://www.godatabase.org/cgi-bin/amigo/go.cgi?query=GO:0006355&view=details) | regulation of transcription, DNA-dependent | 1.93E-122 | 3.56E-119 | 1.68 (17741,2771,5033,1319) |
| [GO:0010468](http://www.godatabase.org/cgi-bin/amigo/go.cgi?query=GO:0010468&view=details) | regulation of gene expression | 1.69E-121 | 2.67E-118 | 1.62 (17741,3207,5033,1470) |
| [GO:0019219](http://www.godatabase.org/cgi-bin/amigo/go.cgi?query=GO:0019219&view=details) | regulation of nucleobase-containing compound metabolic process | 4.72E-121 | 6.54E-118 | 1.61 (17741,3239,5033,1480) |
| [GO:2001141](http://www.godatabase.org/cgi-bin/amigo/go.cgi?query=GO:2001141&view=details) | regulation of RNA biosynthetic process | 1.76E-120 | 2.16E-117 | 1.67 (17741,2792,5033,1322) |
| [GO:2000112](http://www.godatabase.org/cgi-bin/amigo/go.cgi?query=GO:2000112&view=details) | regulation of cellular macromolecule biosynthetic process | 7.45E-118 | 8.24E-115 | 1.63 (17741,3008,5033,1392) |
| [GO:0010556](http://www.godatabase.org/cgi-bin/amigo/go.cgi?query=GO:0010556&view=details) | regulation of macromolecule biosynthetic process | 1.08E-114 | 1.09E-111 | 1.61 (17741,3070,5033,1406) |
| [GO:0051171](http://www.godatabase.org/cgi-bin/amigo/go.cgi?query=GO:0051171&view=details) | regulation of nitrogen compound metabolic process | 2.5E-114 | 2.3E-111 | 1.58 (17741,3319,5033,1491) |
| [GO:0009059](http://www.godatabase.org/cgi-bin/amigo/go.cgi?query=GO:0009059&view=details) | macromolecule biosynthetic process | 1.61E-108 | 1.37E-105 | 1.66 (17741,2606,5033,1228) |
| [GO:0034645](http://www.godatabase.org/cgi-bin/amigo/go.cgi?query=GO:0034645&view=details) | cellular macromolecule biosynthetic process | 1.63E-108 | 1.29E-105 | 1.66 (17741,2592,5033,1223) |
| [GO:0031326](http://www.godatabase.org/cgi-bin/amigo/go.cgi?query=GO:0031326&view=details) | regulation of cellular biosynthetic process | 6.55E-106 | 4.84E-103 | 1.57 (17741,3212,5033,1433) |
| [GO:0009889](http://www.godatabase.org/cgi-bin/amigo/go.cgi?query=GO:0009889&view=details) | regulation of biosynthetic process | 3.83E-104 | 2.65E-101 | 1.56 (17741,3240,5033,1438) |
| [GO:0060255](http://www.godatabase.org/cgi-bin/amigo/go.cgi?query=GO:0060255&view=details) | regulation of macromolecule metabolic process | 1.99E-94 | 1.29E-91 | 1.47 (17741,4013,5033,1669) |
| [GO:0006139](http://www.godatabase.org/cgi-bin/amigo/go.cgi?query=GO:0006139&view=details) | nucleobase-containing compound metabolic process | 1.98E-92 | 1.22E-89 | 1.47 (17741,3872,5033,1617) |
| [GO:0031323](http://www.godatabase.org/cgi-bin/amigo/go.cgi?query=GO:0031323&view=details) | regulation of cellular metabolic process | 5.9E-92 | 3.44E-89 | 1.44 (17741,4245,5033,1737) |
| [GO:0080090](http://www.godatabase.org/cgi-bin/amigo/go.cgi?query=GO:0080090&view=details) | regulation of primary metabolic process | 4.81E-88 | 2.66E-85 | 1.44 (17741,4158,5033,1697) |
| [GO:0006725](http://www.godatabase.org/cgi-bin/amigo/go.cgi?query=GO:0006725&view=details) | cellular aromatic compound metabolic process | 4.78E-82 | 2.52E-79 | 1.43 (17741,4030,5033,1637) |
| [GO:0019222](http://www.godatabase.org/cgi-bin/amigo/go.cgi?query=GO:0019222&view=details) | regulation of metabolic process | 1.91E-80 | 9.6E-78 | 1.39 (17741,4669,5033,1836) |
| [GO:0046483](http://www.godatabase.org/cgi-bin/amigo/go.cgi?query=GO:0046483&view=details) | heterocycle metabolic process | 2.42E-80 | 1.16E-77 | 1.43 (17741,4025,5033,1630) |
| [GO:1901360](http://www.godatabase.org/cgi-bin/amigo/go.cgi?query=GO:1901360&view=details) | organic cyclic compound metabolic process | 1.4E-72 | 6.48E-70 | 1.39 (17741,4207,5033,1663) |
| [GO:0034641](http://www.godatabase.org/cgi-bin/amigo/go.cgi?query=GO:0034641&view=details) | cellular nitrogen compound metabolic process | 1.22E-70 | 5.4E-68 | 1.39 (17741,4165,5033,1643) |
| [GO:0006807](http://www.godatabase.org/cgi-bin/amigo/go.cgi?query=GO:0006807&view=details) | nitrogen compound metabolic process | 5.36E-67 | 2.28E-64 | 1.38 (17741,4211,5033,1645) |
| [GO:0044260](http://www.godatabase.org/cgi-bin/amigo/go.cgi?query=GO:0044260&view=details) | cellular macromolecule metabolic process | 7.59E-63 | 3.11E-60 | 1.30 (17741,5443,5033,2013) |
| [GO:0050794](http://www.godatabase.org/cgi-bin/amigo/go.cgi?query=GO:0050794&view=details) | regulation of cellular process | 1.59E-59 | 6.29E-57 | 1.23 (17741,7499,5033,2611) |
| [GO:0071704](http://www.godatabase.org/cgi-bin/amigo/go.cgi?query=GO:0071704&view=details) | organic substance metabolic process | 3.63E-57 | 1.39E-54 | 1.33 (17741,4562,5033,1719) |
| [GO:0050789](http://www.godatabase.org/cgi-bin/amigo/go.cgi?query=GO:0050789&view=details) | regulation of biological process | 1.69E-51 | 6.25E-49 | 1.20 (17741,7922,5033,2698) |
| [GO:0043170](http://www.godatabase.org/cgi-bin/amigo/go.cgi?query=GO:0043170&view=details) | macromolecule metabolic process | 3.34E-50 | 1.19E-47 | 1.25 (17741,5951,5033,2114) |
| [GO:0044249](http://www.godatabase.org/cgi-bin/amigo/go.cgi?query=GO:0044249&view=details) | cellular biosynthetic process | 2.06E-49 | 7.14E-47 | 1.36 (17741,3546,5033,1368) |
| [GO:0065007](http://www.godatabase.org/cgi-bin/amigo/go.cgi?query=GO:0065007&view=details) | biological regulation | 5.04E-47 | 1.69E-44 | 1.18 (17741,8369,5033,2805) |
| [GO:0009058](http://www.godatabase.org/cgi-bin/amigo/go.cgi?query=GO:0009058&view=details) | biosynthetic process | 1.76E-42 | 5.72E-40 | 1.32 (17741,3704,5033,1390) |
| [GO:0006357](http://www.godatabase.org/cgi-bin/amigo/go.cgi?query=GO:0006357&view=details) | regulation of transcription from RNA polymerase II promoter | 1.17E-23 | 3.71E-21 | 1.48 (17741,1112,5033,466) |
| [GO:0009987](http://www.godatabase.org/cgi-bin/amigo/go.cgi?query=GO:0009987&view=details) | cellular process | 4.14E-22 | 1.27E-19 | 1.08 (17741,11975,5033,3665) |
| [GO:0008380](http://www.godatabase.org/cgi-bin/amigo/go.cgi?query=GO:0008380&view=details) | RNA splicing | 4.17E-22 | 1.25E-19 | 1.94 (17741,292,5033,161) |
| [GO:0071842](http://www.godatabase.org/cgi-bin/amigo/go.cgi?query=GO:0071842&view=details) | cellular component organization at cellular level | 1.6E-20 | 4.66E-18 | 1.27 (17741,2592,5033,935) |
| [GO:0006397](http://www.godatabase.org/cgi-bin/amigo/go.cgi?query=GO:0006397&view=details) | mRNA processing | 4.89E-20 | 1.39E-17 | 1.79 (17741,371,5033,188) |
| [GO:0071841](http://www.godatabase.org/cgi-bin/amigo/go.cgi?query=GO:0071841&view=details) | cellular component organization or biogenesis at cellular level | 4.92E-20 | 1.36E-17 | 1.27 (17741,2626,5033,943) |
| [GO:0022610](http://www.godatabase.org/cgi-bin/amigo/go.cgi?query=GO:0022610&view=details) | biological adhesion | 1.38E-19 | 3.71E-17 | 1.53 (17741,767,5033,332) |
| [GO:0007155](http://www.godatabase.org/cgi-bin/amigo/go.cgi?query=GO:0007155&view=details) | cell adhesion | 1.38E-19 | 3.63E-17 | 1.53 (17741,767,5033,332) |
| [GO:0045893](http://www.godatabase.org/cgi-bin/amigo/go.cgi?query=GO:0045893&view=details) | positive regulation of transcription, DNA-dependent | 2.21E-19 | 5.68E-17 | 1.46 (17741,962,5033,399) |
| [GO:0006996](http://www.godatabase.org/cgi-bin/amigo/go.cgi?query=GO:0006996&view=details) | organelle organization | 2.63E-19 | 6.61E-17 | 1.34 (17741,1681,5033,638) |
| [GO:0051254](http://www.godatabase.org/cgi-bin/amigo/go.cgi?query=GO:0051254&view=details) | positive regulation of RNA metabolic process | 9.58E-19 | 2.36E-16 | 1.44 (17741,1010,5033,413) |
| [GO:0045934](http://www.godatabase.org/cgi-bin/amigo/go.cgi?query=GO:0045934&view=details) | negative regulation of nucleobase-containing compound metabolic process | 1.98E-18 | 4.77E-16 | 1.48 (17741,853,5033,358) |
| [GO:0032502](http://www.godatabase.org/cgi-bin/amigo/go.cgi?query=GO:0032502&view=details) | developmental process | 4.02E-18 | 9.46E-16 | 1.21 (17741,3586,5033,1228) |
| [GO:0051253](http://www.godatabase.org/cgi-bin/amigo/go.cgi?query=GO:0051253&view=details) | negative regulation of RNA metabolic process | 4.55E-18 | 1.05E-15 | 1.50 (17741,776,5033,330) |
| [GO:0051172](http://www.godatabase.org/cgi-bin/amigo/go.cgi?query=GO:0051172&view=details) | negative regulation of nitrogen compound metabolic process | 4.99E-18 | 1.13E-15 | 1.47 (17741,863,5033,360) |
| [GO:0010628](http://www.godatabase.org/cgi-bin/amigo/go.cgi?query=GO:0010628&view=details) | positive regulation of gene expression | 1.61E-17 | 3.57E-15 | 1.42 (17741,1036,5033,417) |
| [GO:0045892](http://www.godatabase.org/cgi-bin/amigo/go.cgi?query=GO:0045892&view=details) | negative regulation of transcription, DNA-dependent | 2.01E-17 | 4.36E-15 | 1.50 (17741,748,5033,318) |
| [GO:2000113](http://www.godatabase.org/cgi-bin/amigo/go.cgi?query=GO:2000113&view=details) | negative regulation of cellular macromolecule biosynthetic process | 3.53E-17 | 7.51E-15 | 1.46 (17741,843,5033,350) |
| [GO:0010558](http://www.godatabase.org/cgi-bin/amigo/go.cgi?query=GO:0010558&view=details) | negative regulation of macromolecule biosynthetic process | 5.11E-17 | 1.07E-14 | 1.45 (17741,871,5033,359) |
| [GO:0010557](http://www.godatabase.org/cgi-bin/amigo/go.cgi?query=GO:0010557&view=details) | positive regulation of macromolecule biosynthetic process | 5.42E-17 | 1.11E-14 | 1.40 (17741,1099,5033,436) |
| [GO:0045935](http://www.godatabase.org/cgi-bin/amigo/go.cgi?query=GO:0045935&view=details) | positive regulation of nucleobase-containing compound metabolic process | 1.35E-16 | 2.72E-14 | 1.39 (17741,1125,5033,443) |
| [GO:0010629](http://www.godatabase.org/cgi-bin/amigo/go.cgi?query=GO:0010629&view=details) | negative regulation of gene expression | 1.94E-16 | 3.84E-14 | 1.46 (17741,819,5033,339) |
| [GO:0051276](http://www.godatabase.org/cgi-bin/amigo/go.cgi?query=GO:0051276&view=details) | chromosome organization | 2.84E-16 | 5.52E-14 | 1.53 (17741,611,5033,266) |
| [GO:0051173](http://www.godatabase.org/cgi-bin/amigo/go.cgi?query=GO:0051173&view=details) | positive regulation of nitrogen compound metabolic process | 3.87E-16 | 7.39E-14 | 1.38 (17741,1146,5033,448) |
| [GO:0007010](http://www.godatabase.org/cgi-bin/amigo/go.cgi?query=GO:0007010&view=details) | cytoskeleton organization | 4.41E-16 | 8.27E-14 | 1.54 (17741,593,5033,259) |
| [GO:0048856](http://www.godatabase.org/cgi-bin/amigo/go.cgi?query=GO:0048856&view=details) | anatomical structure development | 6.95E-16 | 1.28E-13 | 1.26 (17741,2150,5033,770) |
| [GO:0045944](http://www.godatabase.org/cgi-bin/amigo/go.cgi?query=GO:0045944&view=details) | positive regulation of transcription from RNA polymerase II promoter | 1.8E-15 | 3.27E-13 | 1.50 (17741,650,5033,277) |
| [GO:0008150](http://www.godatabase.org/cgi-bin/amigo/go.cgi?query=GO:0008150&view=details) | biological_process | 1.97E-15 | 3.52E-13 | 1.04 (17741,14369,5033,4259) |
| [GO:0016043](http://www.godatabase.org/cgi-bin/amigo/go.cgi?query=GO:0016043&view=details) | cellular component organization | 2.4E-15 | 4.21E-13 | 1.20 (17741,3188,5033,1088) |
| [GO:0071840](http://www.godatabase.org/cgi-bin/amigo/go.cgi?query=GO:0071840&view=details) | cellular component organization or biogenesis | 5.29E-15 | 9.15E-13 | 1.20 (17741,3222,5033,1096) |
| [GO:0007156](http://www.godatabase.org/cgi-bin/amigo/go.cgi?query=GO:0007156&view=details) | homophilic cell adhesion | 1.26E-14 | 2.15E-12 | 2.11 (17741,137,5033,82) |
| [GO:0031327](http://www.godatabase.org/cgi-bin/amigo/go.cgi?query=GO:0031327&view=details) | negative regulation of cellular biosynthetic process | 1.61E-14 | 2.7E-12 | 1.40 (17741,921,5033,366) |
| [GO:0006396](http://www.godatabase.org/cgi-bin/amigo/go.cgi?query=GO:0006396&view=details) | RNA processing | 2.11E-14 | 3.48E-12 | 1.50 (17741,612,5033,260) |
| [GO:0016568](http://www.godatabase.org/cgi-bin/amigo/go.cgi?query=GO:0016568&view=details) | chromatin modification | 2.82E-14 | 4.59E-12 | 1.64 (17741,381,5033,177) |
| [GO:0009890](http://www.godatabase.org/cgi-bin/amigo/go.cgi?query=GO:0009890&view=details) | negative regulation of biosynthetic process | 4.01E-14 | 6.44E-12 | 1.39 (17741,935,5033,369) |
| [GO:0031328](http://www.godatabase.org/cgi-bin/amigo/go.cgi?query=GO:0031328&view=details) | positive regulation of cellular biosynthetic process | 6.99E-14 | 1.11E-11 | 1.34 (17741,1208,5033,458) |
| [GO:0009653](http://www.godatabase.org/cgi-bin/amigo/go.cgi?query=GO:0009653&view=details) | anatomical structure morphogenesis | 9.45E-14 | 1.47E-11 | 1.35 (17741,1100,5033,422) |
| [GO:0009891](http://www.godatabase.org/cgi-bin/amigo/go.cgi?query=GO:0009891&view=details) | positive regulation of biosynthetic process | 1.64E-13 | 2.52E-11 | 1.33 (17741,1232,5033,464) |
| [GO:0007389](http://www.godatabase.org/cgi-bin/amigo/go.cgi?query=GO:0007389&view=details) | pattern specification process | 1.44E-12 | 2.19E-10 | 1.60 (17741,370,5033,168) |
| [GO:0000122](http://www.godatabase.org/cgi-bin/amigo/go.cgi?query=GO:0000122&view=details) | negative regulation of transcription from RNA polymerase II promoter | 1.98E-12 | 2.96E-10 | 1.54 (17741,452,5033,197) |
| [GO:0010605](http://www.godatabase.org/cgi-bin/amigo/go.cgi?query=GO:0010605&view=details) | negative regulation of macromolecule metabolic process | 8.35E-12 | 1.23E-9 | 1.30 (17741,1250,5033,461) |
| [GO:0006325](http://www.godatabase.org/cgi-bin/amigo/go.cgi?query=GO:0006325&view=details) | chromatin organization | 8.6E-11 | 1.25E-8 | 1.48 (17741,471,5033,198) |
| [GO:0003002](http://www.godatabase.org/cgi-bin/amigo/go.cgi?query=GO:0003002&view=details) | regionalization | 1.32E-10 | 1.9E-8 | 1.70 (17741,228,5033,110) |
| [GO:0044238](http://www.godatabase.org/cgi-bin/amigo/go.cgi?query=GO:0044238&view=details) | primary metabolic process | 1.37E-10 | 1.94E-8 | 1.09 (17741,7403,5033,2288) |
| [GO:0016570](http://www.godatabase.org/cgi-bin/amigo/go.cgi?query=GO:0016570&view=details) | histone modification | 1.99E-10 | 2.78E-8 | 1.71 (17741,216,5033,105) |
| [GO:0016569](http://www.godatabase.org/cgi-bin/amigo/go.cgi?query=GO:0016569&view=details) | covalent chromatin modification | 2.24E-10 | 3.1E-8 | 1.71 (17741,219,5033,106) |
| [GO:0048513](http://www.godatabase.org/cgi-bin/amigo/go.cgi?query=GO:0048513&view=details) | organ development | 2.72E-10 | 3.72E-8 | 1.34 (17741,855,5033,325) |
| [GO:0031324](http://www.godatabase.org/cgi-bin/amigo/go.cgi?query=GO:0031324&view=details) | negative regulation of cellular metabolic process | 2.73E-10 | 3.68E-8 | 1.28 (17741,1242,5033,450) |
| [GO:0030029](http://www.godatabase.org/cgi-bin/amigo/go.cgi?query=GO:0030029&view=details) | actin filament-based process | 2.86E-10 | 3.81E-8 | 1.56 (17741,337,5033,149) |
| [GO:0009892](http://www.godatabase.org/cgi-bin/amigo/go.cgi?query=GO:0009892&view=details) | negative regulation of metabolic process | 3.78E-10 | 4.98E-8 | 1.26 (17741,1339,5033,480) |
| [GO:0000375](http://www.godatabase.org/cgi-bin/amigo/go.cgi?query=GO:0000375&view=details) | RNA splicing, via transesterification reactions | 4.31E-10 | 5.61E-8 | 1.77 (17741,179,5033,90) |
| [GO:0000377](http://www.godatabase.org/cgi-bin/amigo/go.cgi?query=GO:0000377&view=details) | RNA splicing, via transesterification reactions with bulged adenosine as nucleophile | 5.68E-10 | 7.31E-8 | 1.78 (17741,172,5033,87) |
| [GO:0000398](http://www.godatabase.org/cgi-bin/amigo/go.cgi?query=GO:0000398&view=details) | mRNA splicing, via spliceosome | 5.68E-10 | 7.23E-8 | 1.78 (17741,172,5033,87) |
| [GO:0007411](http://www.godatabase.org/cgi-bin/amigo/go.cgi?query=GO:0007411&view=details) | axon guidance | 5.84E-10 | 7.34E-8 | 1.55 (17741,337,5033,148) |

Function


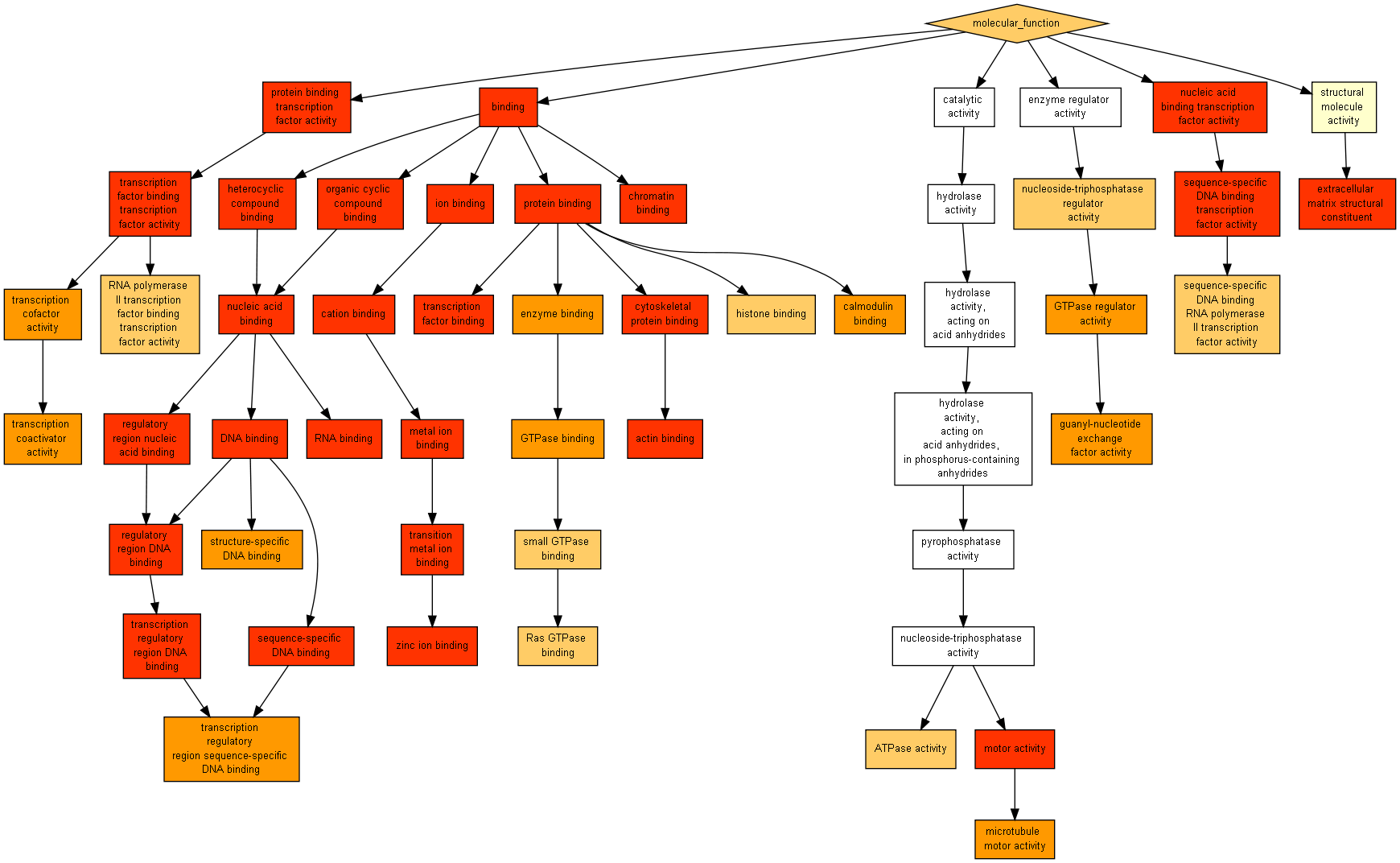


| **GO term** | **Description** | [**P-value**](http://cbl-gorilla.cs.technion.ac.il/GOrilla/c8ijves3/GOResultsFUNCTION.html#p_value_info) | [**FDR q-value**](http://cbl-gorilla.cs.technion.ac.il/GOrilla/c8ijves3/GOResultsFUNCTION.html#fdr_info) | [**Enrichment (N, B, n, b)**](http://cbl-gorilla.cs.technion.ac.il/GOrilla/c8ijves3/GOResultsFUNCTION.html#enrich_info) |
| --- | --- | --- | --- | --- |
| [GO:0003676](http://www.godatabase.org/cgi-bin/amigo/go.cgi?query=GO:0003676&view=details) | nucleic acid binding | 1.5E-165 | 5.73E-162 | 1.71 (17741,3278,5033,1593) |
| [GO:0003677](http://www.godatabase.org/cgi-bin/amigo/go.cgi?query=GO:0003677&view=details) | DNA binding | 6.09E-155 | 1.16E-151 | 1.86 (17741,2316,5033,1219) |
| [GO:0008270](http://www.godatabase.org/cgi-bin/amigo/go.cgi?query=GO:0008270&view=details) | zinc ion binding | 3.48E-109 | 4.43E-106 | 1.78 (17741,1972,5033,998) |
| [GO:0046914](http://www.godatabase.org/cgi-bin/amigo/go.cgi?query=GO:0046914&view=details) | transition metal ion binding | 5.32E-91 | 5.09E-88 | 1.67 (17741,2206,5033,1043) |
| [GO:1901363](http://www.godatabase.org/cgi-bin/amigo/go.cgi?query=GO:1901363&view=details) | heterocyclic compound binding | 1.12E-89 | 8.54E-87 | 1.37 (17741,5294,5033,2062) |
| [GO:0097159](http://www.godatabase.org/cgi-bin/amigo/go.cgi?query=GO:0097159&view=details) | organic cyclic compound binding | 1.39E-89 | 8.87E-87 | 1.37 (17741,5295,5033,2062) |
| [GO:0005488](http://www.godatabase.org/cgi-bin/amigo/go.cgi?query=GO:0005488&view=details) | binding | 1.14E-77 | 6.2E-75 | 1.16 (17741,11726,5033,3846) |
| [GO:0046872](http://www.godatabase.org/cgi-bin/amigo/go.cgi?query=GO:0046872&view=details) | metal ion binding | 1.06E-58 | 5.06E-56 | 1.37 (17741,3887,5033,1512) |
| [GO:0043169](http://www.godatabase.org/cgi-bin/amigo/go.cgi?query=GO:0043169&view=details) | cation binding | 5.44E-55 | 2.31E-52 | 1.35 (17741,3959,5033,1521) |
| [GO:0043167](http://www.godatabase.org/cgi-bin/amigo/go.cgi?query=GO:0043167&view=details) | ion binding | 1.66E-54 | 6.34E-52 | 1.35 (17741,3968,5033,1522) |
| [GO:0003700](http://www.godatabase.org/cgi-bin/amigo/go.cgi?query=GO:0003700&view=details) | sequence-specific DNA binding transcription factor activity | 9.84E-34 | 3.42E-31 | 1.62 (17741,991,5033,455) |
| [GO:0001071](http://www.godatabase.org/cgi-bin/amigo/go.cgi?query=GO:0001071&view=details) | nucleic acid binding transcription factor activity | 1.78E-33 | 5.66E-31 | 1.62 (17741,993,5033,455) |
| [GO:0008092](http://www.godatabase.org/cgi-bin/amigo/go.cgi?query=GO:0008092&view=details) | cytoskeletal protein binding | 2.61E-23 | 7.67E-21 | 1.66 (17741,611,5033,287) |
| [GO:0003779](http://www.godatabase.org/cgi-bin/amigo/go.cgi?query=GO:0003779&view=details) | actin binding | 5.24E-22 | 1.43E-19 | 1.86 (17741,347,5033,183) |
| [GO:0005515](http://www.godatabase.org/cgi-bin/amigo/go.cgi?query=GO:0005515&view=details) | protein binding | 5.87E-21 | 1.5E-18 | 1.14 (17741,7059,5033,2278) |
| [GO:0043565](http://www.godatabase.org/cgi-bin/amigo/go.cgi?query=GO:0043565&view=details) | sequence-specific DNA binding | 3.69E-20 | 8.83E-18 | 1.58 (17741,669,5033,299) |
| [GO:0044212](http://www.godatabase.org/cgi-bin/amigo/go.cgi?query=GO:0044212&view=details) | transcription regulatory region DNA binding | 4.56E-17 | 1.03E-14 | 1.77 (17741,322,5033,162) |
| [GO:0000975](http://www.godatabase.org/cgi-bin/amigo/go.cgi?query=GO:0000975&view=details) | regulatory region DNA binding | 1.28E-16 | 2.72E-14 | 1.75 (17741,330,5033,164) |
| [GO:0001067](http://www.godatabase.org/cgi-bin/amigo/go.cgi?query=GO:0001067&view=details) | regulatory region nucleic acid binding | 1.28E-16 | 2.58E-14 | 1.75 (17741,330,5033,164) |
| [GO:0003682](http://www.godatabase.org/cgi-bin/amigo/go.cgi?query=GO:0003682&view=details) | chromatin binding | 4.98E-16 | 9.53E-14 | 1.85 (17741,254,5033,133) |
| [GO:0003723](http://www.godatabase.org/cgi-bin/amigo/go.cgi?query=GO:0003723&view=details) | RNA binding | 9.26E-15 | 1.69E-12 | 1.43 (17741,838,5033,339) |
| [GO:0008134](http://www.godatabase.org/cgi-bin/amigo/go.cgi?query=GO:0008134&view=details) | transcription factor binding | 4.57E-14 | 7.95E-12 | 1.62 (17741,399,5033,183) |
| [GO:0003774](http://www.godatabase.org/cgi-bin/amigo/go.cgi?query=GO:0003774&view=details) | motor activity | 9.91E-14 | 1.65E-11 | 2.10 (17741,129,5033,77) |
| [GO:0005201](http://www.godatabase.org/cgi-bin/amigo/go.cgi?query=GO:0005201&view=details) | extracellular matrix structural constituent | 1.14E-11 | 1.82E-9 | 2.30 (17741,78,5033,51) |
| [GO:0000988](http://www.godatabase.org/cgi-bin/amigo/go.cgi?query=GO:0000988&view=details) | protein binding transcription factor activity | 2.19E-10 | 3.36E-8 | 1.46 (17741,484,5033,201) |
| [GO:0000989](http://www.godatabase.org/cgi-bin/amigo/go.cgi?query=GO:0000989&view=details) | transcription factor binding transcription factor activity | 3.63E-10 | 5.34E-8 | 1.47 (17741,469,5033,195) |
| [GO:0003712](http://www.godatabase.org/cgi-bin/amigo/go.cgi?query=GO:0003712&view=details) | transcription cofactor activity | 1.65E-9 | 2.33E-7 | 1.46 (17741,453,5033,187) |
| [GO:0019899](http://www.godatabase.org/cgi-bin/amigo/go.cgi?query=GO:0019899&view=details) | enzyme binding | 4.23E-9 | 5.78E-7 | 1.28 (17741,1035,5033,377) |
| [GO:0051020](http://www.godatabase.org/cgi-bin/amigo/go.cgi?query=GO:0051020&view=details) | GTPase binding | 5.48E-9 | 7.22E-7 | 1.81 (17741,142,5033,73) |
| [GO:0043566](http://www.godatabase.org/cgi-bin/amigo/go.cgi?query=GO:0043566&view=details) | structure-specific DNA binding | 1.64E-8 | 2.09E-6 | 1.62 (17741,222,5033,102) |
| [GO:0005085](http://www.godatabase.org/cgi-bin/amigo/go.cgi?query=GO:0005085&view=details) | guanyl-nucleotide exchange factor activity | 2.81E-8 | 3.47E-6 | 1.72 (17741,162,5033,79) |
| [GO:0003713](http://www.godatabase.org/cgi-bin/amigo/go.cgi?query=GO:0003713&view=details) | transcription coactivator activity | 3.24E-8 | 3.87E-6 | 1.55 (17741,266,5033,117) |
| [GO:0000976](http://www.godatabase.org/cgi-bin/amigo/go.cgi?query=GO:0000976&view=details) | transcription regulatory region sequence-specific DNA binding | 4.83E-8 | 5.59E-6 | 1.76 (17741,140,5033,70) |
| [GO:0005516](http://www.godatabase.org/cgi-bin/amigo/go.cgi?query=GO:0005516&view=details) | calmodulin binding | 4.91E-8 | 5.52E-6 | 1.71 (17741,161,5033,78) |
| [GO:0003777](http://www.godatabase.org/cgi-bin/amigo/go.cgi?query=GO:0003777&view=details) | microtubule motor activity | 8.2E-8 | 8.96E-6 | 2.05 (17741,74,5033,43) |
| [GO:0030695](http://www.godatabase.org/cgi-bin/amigo/go.cgi?query=GO:0030695&view=details) | GTPase regulator activity | 8.25E-8 | 8.76E-6 | 1.42 (17741,423,5033,170) |
| [GO:0016887](http://www.godatabase.org/cgi-bin/amigo/go.cgi?query=GO:0016887&view=details) | ATPase activity | 2.73E-7 | 2.82E-5 | 1.44 (17741,347,5033,142) |
| [GO:0031267](http://www.godatabase.org/cgi-bin/amigo/go.cgi?query=GO:0031267&view=details) | small GTPase binding | 2.76E-7 | 2.78E-5 | 1.76 (17741,124,5033,62) |
| [GO:0060589](http://www.godatabase.org/cgi-bin/amigo/go.cgi?query=GO:0060589&view=details) | nucleoside-triphosphatase regulator activity | 3E-7 | 2.94E-5 | 1.39 (17741,436,5033,172) |
| [GO:0000981](http://www.godatabase.org/cgi-bin/amigo/go.cgi?query=GO:0000981&view=details) | sequence-specific DNA binding RNA polymerase II transcription factor activity | 3.18E-7 | 3.04E-5 | 1.54 (17741,233,5033,102) |
| [GO:0001076](http://www.godatabase.org/cgi-bin/amigo/go.cgi?query=GO:0001076&view=details) | RNA polymerase II transcription factor binding transcription factor activity | 3.31E-7 | 3.09E-5 | 1.93 (17741,84,5033,46) |
| [GO:0042393](http://www.godatabase.org/cgi-bin/amigo/go.cgi?query=GO:0042393&view=details) | histone binding | 3.37E-7 | 3.07E-5 | 1.83 (17741,104,5033,54) |
| [GO:0017016](http://www.godatabase.org/cgi-bin/amigo/go.cgi?query=GO:0017016&view=details) | Ras GTPase binding | 5.67E-7 | 5.04E-5 | 1.78 (17741,113,5033,57) |

Component


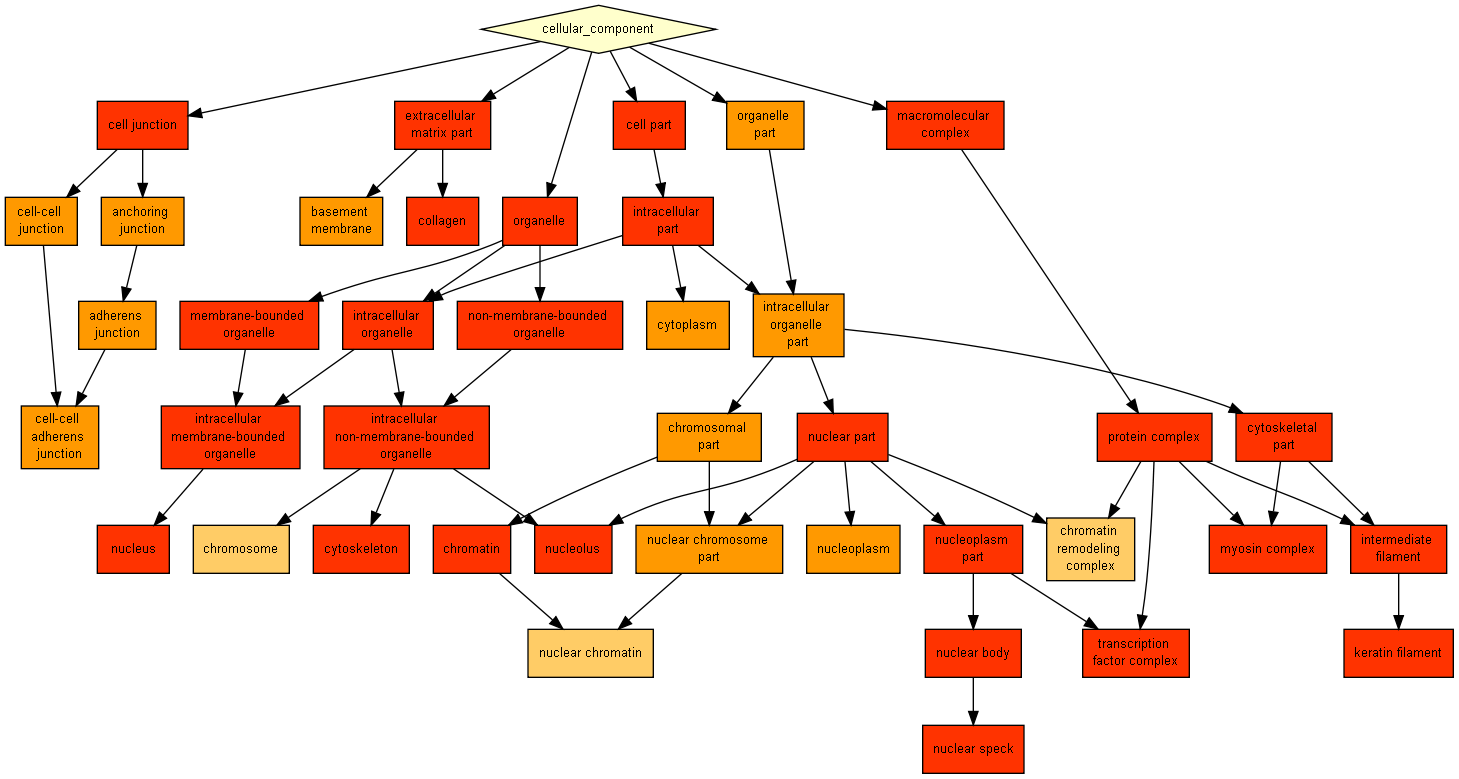


| **GO term** | **Description** | [**P-value**](http://cbl-gorilla.cs.technion.ac.il/GOrilla/c8ijves3/GOResultsCOMPONENT.html#p_value_info) | [**FDR q-value**](http://cbl-gorilla.cs.technion.ac.il/GOrilla/c8ijves3/GOResultsCOMPONENT.html#fdr_info) | [**Enrichment (N, B, n, b)**](http://cbl-gorilla.cs.technion.ac.il/GOrilla/c8ijves3/GOResultsCOMPONENT.html#enrich_info) |
| --- | --- | --- | --- | --- |
| [GO:0005634](http://www.godatabase.org/cgi-bin/amigo/go.cgi?query=GO:0005634&view=details) | nucleus | 1.36E-119 | 1.73E-116 | 1.47 (17741,4741,5033,1976) |
| [GO:0043229](http://www.godatabase.org/cgi-bin/amigo/go.cgi?query=GO:0043229&view=details) | intracellular organelle | 1.61E-65 | 1.03E-62 | 1.23 (17741,7999,5033,2780) |
| [GO:0043226](http://www.godatabase.org/cgi-bin/amigo/go.cgi?query=GO:0043226&view=details) | organelle | 2.67E-65 | 1.14E-62 | 1.22 (17741,8016,5033,2784) |
| [GO:0044464](http://www.godatabase.org/cgi-bin/amigo/go.cgi?query=GO:0044464&view=details) | cell part | 1.11E-49 | 3.54E-47 | 1.11 (17741,12594,5033,3968) |
| [GO:0043231](http://www.godatabase.org/cgi-bin/amigo/go.cgi?query=GO:0043231&view=details) | intracellular membrane-bounded organelle | 2.55E-48 | 6.52E-46 | 1.21 (17741,7072,5033,2437) |
| [GO:0043227](http://www.godatabase.org/cgi-bin/amigo/go.cgi?query=GO:0043227&view=details) | membrane-bounded organelle | 3.2E-48 | 6.83E-46 | 1.21 (17741,7077,5033,2438) |
| [GO:0044424](http://www.godatabase.org/cgi-bin/amigo/go.cgi?query=GO:0044424&view=details) | intracellular part | 8.92E-48 | 1.63E-45 | 1.12 (17741,12000,5033,3805) |
| [GO:0044428](http://www.godatabase.org/cgi-bin/amigo/go.cgi?query=GO:0044428&view=details) | nuclear part | 5.64E-43 | 9.01E-41 | 1.42 (17741,2428,5033,980) |
| [GO:0044451](http://www.godatabase.org/cgi-bin/amigo/go.cgi?query=GO:0044451&view=details) | nucleoplasm part | 2.89E-35 | 4.1E-33 | 1.72 (17741,793,5033,386) |
| [GO:0044430](http://www.godatabase.org/cgi-bin/amigo/go.cgi?query=GO:0044430&view=details) | cytoskeletal part | 5.07E-30 | 6.48E-28 | 1.51 (17741,1237,5033,531) |
| [GO:0043228](http://www.godatabase.org/cgi-bin/amigo/go.cgi?query=GO:0043228&view=details) | non-membrane-bounded organelle | 1.46E-25 | 1.7E-23 | 1.36 (17741,1932,5033,748) |
| [GO:0043232](http://www.godatabase.org/cgi-bin/amigo/go.cgi?query=GO:0043232&view=details) | intracellular non-membrane-bounded organelle | 1.46E-25 | 1.56E-23 | 1.36 (17741,1932,5033,748) |
| [GO:0016604](http://www.godatabase.org/cgi-bin/amigo/go.cgi?query=GO:0016604&view=details) | nuclear body | 4.65E-22 | 4.58E-20 | 1.99 (17741,268,5033,151) |
| [GO:0045095](http://www.godatabase.org/cgi-bin/amigo/go.cgi?query=GO:0045095&view=details) | keratin filament | 8.66E-22 | 7.91E-20 | 2.71 (17741,91,5033,70) |
| [GO:0016607](http://www.godatabase.org/cgi-bin/amigo/go.cgi?query=GO:0016607&view=details) | nuclear speck | 2.66E-20 | 2.26E-18 | 2.29 (17741,146,5033,95) |
| [GO:0044420](http://www.godatabase.org/cgi-bin/amigo/go.cgi?query=GO:0044420&view=details) | extracellular matrix part | 7.86E-20 | 6.27E-18 | 2.17 (17741,172,5033,106) |
| [GO:0005581](http://www.godatabase.org/cgi-bin/amigo/go.cgi?query=GO:0005581&view=details) | collagen | 1.38E-19 | 1.04E-17 | 2.64 (17741,88,5033,66) |
| [GO:0005882](http://www.godatabase.org/cgi-bin/amigo/go.cgi?query=GO:0005882&view=details) | intermediate filament | 8.04E-17 | 5.71E-15 | 2.03 (17741,184,5033,106) |
| [GO:0043234](http://www.godatabase.org/cgi-bin/amigo/go.cgi?query=GO:0043234&view=details) | protein complex | 5.15E-13 | 3.47E-11 | 1.18 (17741,3187,5033,1071) |
| [GO:0032991](http://www.godatabase.org/cgi-bin/amigo/go.cgi?query=GO:0032991&view=details) | macromolecular complex | 6.41E-13 | 4.1E-11 | 1.17 (17741,3756,5033,1242) |
| [GO:0005856](http://www.godatabase.org/cgi-bin/amigo/go.cgi?query=GO:0005856&view=details) | cytoskeleton | 4.84E-12 | 2.94E-10 | 1.43 (17741,655,5033,266) |
| [GO:0005730](http://www.godatabase.org/cgi-bin/amigo/go.cgi?query=GO:0005730&view=details) | nucleolus | 2E-11 | 1.16E-9 | 1.45 (17741,583,5033,239) |
| [GO:0030054](http://www.godatabase.org/cgi-bin/amigo/go.cgi?query=GO:0030054&view=details) | cell junction | 4.05E-10 | 2.25E-8 | 1.38 (17741,697,5033,272) |
| [GO:0016459](http://www.godatabase.org/cgi-bin/amigo/go.cgi?query=GO:0016459&view=details) | myosin complex | 6.3E-10 | 3.36E-8 | 2.31 (17741,64,5033,42) |
| [GO:0005667](http://www.godatabase.org/cgi-bin/amigo/go.cgi?query=GO:0005667&view=details) | transcription factor complex | 7.21E-10 | 3.69E-8 | 1.61 (17741,274,5033,125) |
| [GO:0000785](http://www.godatabase.org/cgi-bin/amigo/go.cgi?query=GO:0000785&view=details) | chromatin | 9.05E-10 | 4.45E-8 | 1.71 (17741,202,5033,98) |
| [GO:0005737](http://www.godatabase.org/cgi-bin/amigo/go.cgi?query=GO:0005737&view=details) | cytoplasm | 1.27E-9 | 5.99E-8 | 1.13 (17741,3929,5033,1265) |
| [GO:0070161](http://www.godatabase.org/cgi-bin/amigo/go.cgi?query=GO:0070161&view=details) | anchoring junction | 2.44E-9 | 1.11E-7 | 1.69 (17741,205,5033,98) |
| [GO:0005911](http://www.godatabase.org/cgi-bin/amigo/go.cgi?query=GO:0005911&view=details) | cell-cell junction | 4.41E-9 | 1.94E-7 | 1.59 (17741,264,5033,119) |
| [GO:0044422](http://www.godatabase.org/cgi-bin/amigo/go.cgi?query=GO:0044422&view=details) | organelle part | 6.63E-9 | 2.82E-7 | 1.09 (17741,6201,5033,1923) |
| [GO:0005604](http://www.godatabase.org/cgi-bin/amigo/go.cgi?query=GO:0005604&view=details) | basement membrane | 6.8E-9 | 2.8E-7 | 2.24 (17741,63,5033,40) |
| [GO:0005912](http://www.godatabase.org/cgi-bin/amigo/go.cgi?query=GO:0005912&view=details) | adherens junction | 9.89E-9 | 3.95E-7 | 1.69 (17741,188,5033,90) |
| [GO:0044454](http://www.godatabase.org/cgi-bin/amigo/go.cgi?query=GO:0044454&view=details) | nuclear chromosome part | 1.01E-8 | 3.93E-7 | 1.61 (17741,234,5033,107) |
| [GO:0044427](http://www.godatabase.org/cgi-bin/amigo/go.cgi?query=GO:0044427&view=details) | chromosomal part | 2.35E-8 | 8.82E-7 | 1.39 (17741,508,5033,201) |
| [GO:0005913](http://www.godatabase.org/cgi-bin/amigo/go.cgi?query=GO:0005913&view=details) | cell-cell adherens junction | 4.82E-8 | 1.76E-6 | 2.40 (17741,44,5033,30) |
| [GO:0005654](http://www.godatabase.org/cgi-bin/amigo/go.cgi?query=GO:0005654&view=details) | nucleoplasm | 6.34E-8 | 2.25E-6 | 1.28 (17741,925,5033,335) |
| [GO:0044446](http://www.godatabase.org/cgi-bin/amigo/go.cgi?query=GO:0044446&view=details) | intracellular organelle part | 8.6E-8 | 2.97E-6 | 1.09 (17741,6115,5033,1885) |
| [GO:0000790](http://www.godatabase.org/cgi-bin/amigo/go.cgi?query=GO:0000790&view=details) | nuclear chromatin | 1.76E-7 | 5.92E-6 | 1.83 (17741,110,5033,57) |
| [GO:0016585](http://www.godatabase.org/cgi-bin/amigo/go.cgi?query=GO:0016585&view=details) | chromatin remodeling complex | 2.22E-7 | 7.27E-6 | 1.76 (17741,126,5033,63) |
| [GO:0005694](http://www.godatabase.org/cgi-bin/amigo/go.cgi?query=GO:0005694&view=details) | chromosome | 2.95E-7 | 9.41E-6 | 1.74 (17741,132,5033,65) |

**2) RC - Ranked list test** of 5511 human compositionally ordered proteins with p-value threshold of 10^-6^ yields:

Process


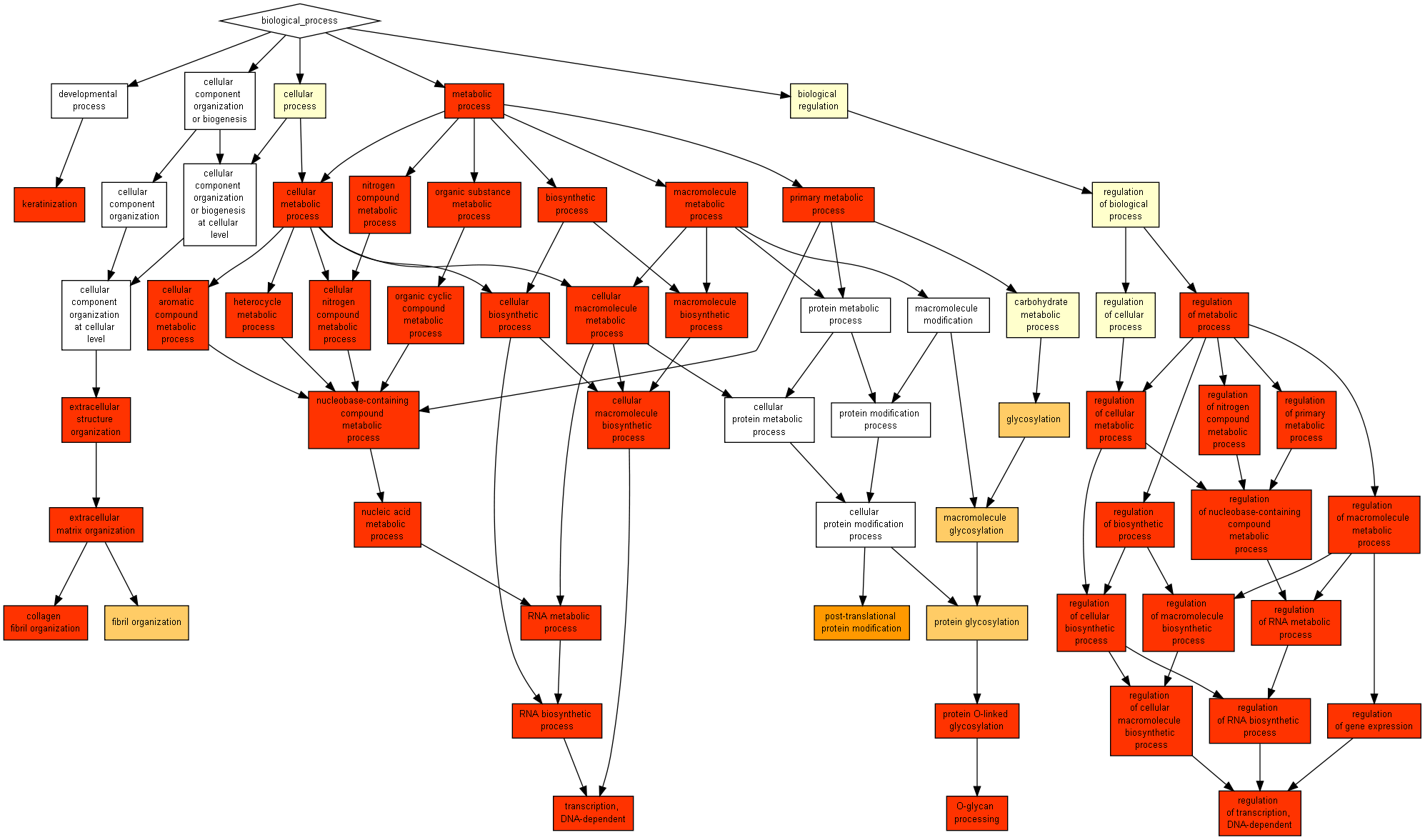


| **GO term** | **Description** | [**P-value**](http://cbl-gorilla.cs.technion.ac.il/GOrilla/v9ffx2p8/GOResultsPROCESS.html#p_value_info) | [**FDR q-value**](http://cbl-gorilla.cs.technion.ac.il/GOrilla/v9ffx2p8/GOResultsPROCESS.html#fdr_info) | [**Enrichment (N, B, n, b)**](http://cbl-gorilla.cs.technion.ac.il/GOrilla/v9ffx2p8/GOResultsPROCESS.html#enrich_info) |
| --- | --- | --- | --- | --- |
| [GO:0006351](http://www.godatabase.org/cgi-bin/amigo/go.cgi?query=GO:0006351&view=details) | transcription, DNA-dependent | 1.87E-56 | 1.39E-52 | 1.90 (5033,1140,991,427) |
| [GO:0032774](http://www.godatabase.org/cgi-bin/amigo/go.cgi?query=GO:0032774&view=details) | RNA biosynthetic process | 1.65E-54 | 6.1E-51 | 1.86 (5033,1187,991,435) |
| [GO:0009059](http://www.godatabase.org/cgi-bin/amigo/go.cgi?query=GO:0009059&view=details) | macromolecule biosynthetic process | 6.16E-53 | 1.52E-49 | 1.87 (5033,1228,924,421) |
| [GO:0034645](http://www.godatabase.org/cgi-bin/amigo/go.cgi?query=GO:0034645&view=details) | cellular macromolecule biosynthetic process | 5.31E-52 | 9.84E-49 | 1.82 (5033,1223,991,439) |
| [GO:0016070](http://www.godatabase.org/cgi-bin/amigo/go.cgi?query=GO:0016070&view=details) | RNA metabolic process | 6.54E-50 | 9.7E-47 | 1.72 (5033,1423,988,481) |
| [GO:0090304](http://www.godatabase.org/cgi-bin/amigo/go.cgi?query=GO:0090304&view=details) | nucleic acid metabolic process | 5.19E-43 | 6.41E-40 | 1.62 (5033,1558,996,500) |
| [GO:0044249](http://www.godatabase.org/cgi-bin/amigo/go.cgi?query=GO:0044249&view=details) | cellular biosynthetic process | 4.31E-42 | 4.57E-39 | 1.68 (5033,1368,991,453) |
| [GO:0009058](http://www.godatabase.org/cgi-bin/amigo/go.cgi?query=GO:0009058&view=details) | biosynthetic process | 9.55E-42 | 8.85E-39 | 1.80 (5033,1390,758,376) |
| [GO:0051252](http://www.godatabase.org/cgi-bin/amigo/go.cgi?query=GO:0051252&view=details) | regulation of RNA metabolic process | 4.34E-39 | 3.57E-36 | 1.68 (5033,1356,947,429) |
| [GO:0046483](http://www.godatabase.org/cgi-bin/amigo/go.cgi?query=GO:0046483&view=details) | heterocycle metabolic process | 4.95E-39 | 3.67E-36 | 1.57 (5033,1630,996,507) |
| [GO:0006139](http://www.godatabase.org/cgi-bin/amigo/go.cgi?query=GO:0006139&view=details) | nucleobase-containing compound metabolic process | 5.65E-39 | 3.8E-36 | 1.58 (5033,1617,996,504) |
| [GO:0006725](http://www.godatabase.org/cgi-bin/amigo/go.cgi?query=GO:0006725&view=details) | cellular aromatic compound metabolic process | 8.46E-39 | 5.23E-36 | 1.57 (5033,1637,996,508) |
| [GO:0006355](http://www.godatabase.org/cgi-bin/amigo/go.cgi?query=GO:0006355&view=details) | regulation of transcription, DNA-dependent | 2.81E-38 | 1.6E-35 | 1.69 (5033,1319,947,419) |
| [GO:1901360](http://www.godatabase.org/cgi-bin/amigo/go.cgi?query=GO:1901360&view=details) | organic cyclic compound metabolic process | 4.53E-38 | 2.4E-35 | 1.56 (5033,1663,996,512) |
| [GO:2001141](http://www.godatabase.org/cgi-bin/amigo/go.cgi?query=GO:2001141&view=details) | regulation of RNA biosynthetic process | 5.6E-38 | 2.77E-35 | 1.68 (5033,1322,947,419) |
| [GO:0034641](http://www.godatabase.org/cgi-bin/amigo/go.cgi?query=GO:0034641&view=details) | cellular nitrogen compound metabolic process | 8.27E-38 | 3.83E-35 | 1.56 (5033,1643,996,507) |
| [GO:0006807](http://www.godatabase.org/cgi-bin/amigo/go.cgi?query=GO:0006807&view=details) | nitrogen compound metabolic process | 1.27E-37 | 5.53E-35 | 1.56 (5033,1645,996,507) |
| [GO:0071704](http://www.godatabase.org/cgi-bin/amigo/go.cgi?query=GO:0071704&view=details) | organic substance metabolic process | 1E-36 | 4.12E-34 | 1.53 (5033,1719,996,521) |
| [GO:2000112](http://www.godatabase.org/cgi-bin/amigo/go.cgi?query=GO:2000112&view=details) | regulation of cellular macromolecule biosynthetic process | 7.81E-35 | 3.05E-32 | 1.61 (5033,1392,988,441) |
| [GO:0010556](http://www.godatabase.org/cgi-bin/amigo/go.cgi?query=GO:0010556&view=details) | regulation of macromolecule biosynthetic process | 1.63E-33 | 6.04E-31 | 1.60 (5033,1406,988,441) |
| [GO:0010468](http://www.godatabase.org/cgi-bin/amigo/go.cgi?query=GO:0010468&view=details) | regulation of gene expression | 5.94E-33 | 2.1E-30 | 1.57 (5033,1470,988,454) |
| [GO:0031326](http://www.godatabase.org/cgi-bin/amigo/go.cgi?query=GO:0031326&view=details) | regulation of cellular biosynthetic process | 2.74E-32 | 9.25E-30 | 1.59 (5033,1433,947,430) |
| [GO:0009889](http://www.godatabase.org/cgi-bin/amigo/go.cgi?query=GO:0009889&view=details) | regulation of biosynthetic process | 2.95E-32 | 9.52E-30 | 1.59 (5033,1438,947,431) |
| [GO:0019219](http://www.godatabase.org/cgi-bin/amigo/go.cgi?query=GO:0019219&view=details) | regulation of nucleobase-containing compound metabolic process | 3.02E-30 | 9.33E-28 | 1.56 (5033,1480,947,435) |
| [GO:0051171](http://www.godatabase.org/cgi-bin/amigo/go.cgi?query=GO:0051171&view=details) | regulation of nitrogen compound metabolic process | 2.5E-29 | 7.4E-27 | 1.55 (5033,1491,947,435) |
| [GO:0044260](http://www.godatabase.org/cgi-bin/amigo/go.cgi?query=GO:0044260&view=details) | cellular macromolecule metabolic process | 9.26E-28 | 2.64E-25 | 1.41 (5033,2013,996,560) |
| [GO:0043170](http://www.godatabase.org/cgi-bin/amigo/go.cgi?query=GO:0043170&view=details) | macromolecule metabolic process | 1.08E-23 | 2.96E-21 | 1.36 (5033,2114,996,569) |
| [GO:0060255](http://www.godatabase.org/cgi-bin/amigo/go.cgi?query=GO:0060255&view=details) | regulation of macromolecule metabolic process | 3.88E-23 | 1.03E-20 | 1.44 (5033,1669,988,471) |
| [GO:0080090](http://www.godatabase.org/cgi-bin/amigo/go.cgi?query=GO:0080090&view=details) | regulation of primary metabolic process | 8.15E-21 | 2.08E-18 | 1.42 (5033,1697,947,454) |
| [GO:0031323](http://www.godatabase.org/cgi-bin/amigo/go.cgi?query=GO:0031323&view=details) | regulation of cellular metabolic process | 1.48E-18 | 3.66E-16 | 1.39 (5033,1737,947,455) |
| [GO:0044237](http://www.godatabase.org/cgi-bin/amigo/go.cgi?query=GO:0044237&view=details) | cellular metabolic process | 2.97E-18 | 7.1E-16 | 1.30 (5033,2224,996,574) |
| [GO:0030198](http://www.godatabase.org/cgi-bin/amigo/go.cgi?query=GO:0030198&view=details) | extracellular matrix organization | 8.55E-18 | 1.98E-15 | 6.58 (5033,82,308,33) |
| [GO:0043062](http://www.godatabase.org/cgi-bin/amigo/go.cgi?query=GO:0043062&view=details) | extracellular structure organization | 1.36E-17 | 3.05E-15 | 6.50 (5033,83,308,33) |
| [GO:0019222](http://www.godatabase.org/cgi-bin/amigo/go.cgi?query=GO:0019222&view=details) | regulation of metabolic process | 2.1E-16 | 4.58E-14 | 1.43 (5033,1836,700,364) |
| [GO:0044238](http://www.godatabase.org/cgi-bin/amigo/go.cgi?query=GO:0044238&view=details) | primary metabolic process | 6.69E-15 | 1.42E-12 | 1.27 (5033,2288,996,575) |
| [GO:0008152](http://www.godatabase.org/cgi-bin/amigo/go.cgi?query=GO:0008152&view=details) | metabolic process | 5.62E-13 | 1.16E-10 | 1.24 (5033,2380,996,586) |
| [GO:0016266](http://www.godatabase.org/cgi-bin/amigo/go.cgi?query=GO:0016266&view=details) | O-glycan processing | 6.62E-13 | 1.33E-10 | 31.16 (5033,17,95,10) |
| [GO:0006493](http://www.godatabase.org/cgi-bin/amigo/go.cgi?query=GO:0006493&view=details) | protein O-linked glycosylation | 1.27E-11 | 2.48E-9 | 25.23 (5033,21,95,10) |
| [GO:0031424](http://www.godatabase.org/cgi-bin/amigo/go.cgi?query=GO:0031424&view=details) | keratinization | 2.28E-11 | 4.34E-9 | 4.28 (5033,26,994,22) |
| [GO:0030199](http://www.godatabase.org/cgi-bin/amigo/go.cgi?query=GO:0030199&view=details) | collagen fibril organization | 5.9E-10 | 1.09E-7 | 16.89 (5033,20,149,10) |
| [GO:0043687](http://www.godatabase.org/cgi-bin/amigo/go.cgi?query=GO:0043687&view=details) | post-translational protein modification | 5.75E-8 | 1.04E-5 | 12.32 (5033,43,95,10) |
| [GO:0006486](http://www.godatabase.org/cgi-bin/amigo/go.cgi?query=GO:0006486&view=details) | protein glycosylation | 3.57E-7 | 6.31E-5 | 10.39 (5033,51,95,10) |
| [GO:0043413](http://www.godatabase.org/cgi-bin/amigo/go.cgi?query=GO:0043413&view=details) | macromolecule glycosylation | 3.57E-7 | 6.16E-5 | 10.39 (5033,51,95,10) |
| [GO:0070085](http://www.godatabase.org/cgi-bin/amigo/go.cgi?query=GO:0070085&view=details) | glycosylation | 3.57E-7 | 6.02E-5 | 10.39 (5033,51,95,10) |
| [GO:0043206](http://www.godatabase.org/cgi-bin/amigo/go.cgi?query=GO:0043206&view=details) | fibril organization | 6.77E-7 | 1.12E-4 | 40.26 (5033,4,125,4) |

Function


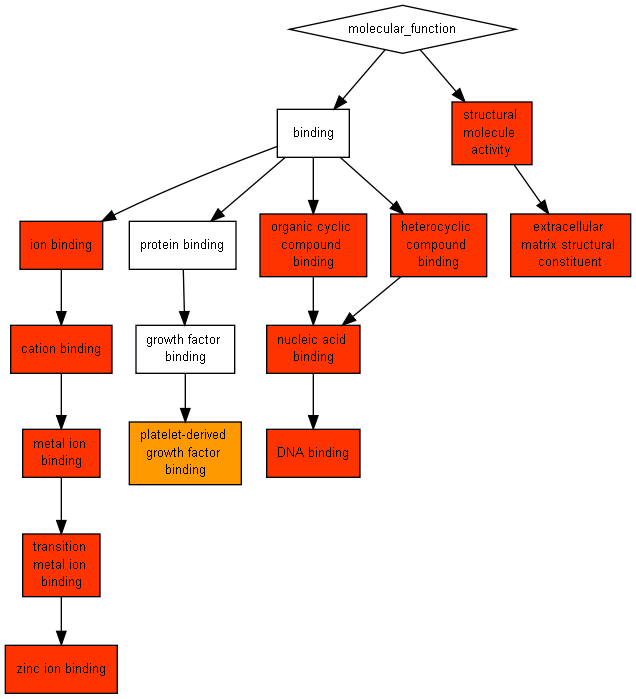


| **GO term** | **Description** | [**P-value**](http://cbl-gorilla.cs.technion.ac.il/GOrilla/v9ffx2p8/GOResultsFUNCTION.html#p_value_info) | [**FDR q-value**](http://cbl-gorilla.cs.technion.ac.il/GOrilla/v9ffx2p8/GOResultsFUNCTION.html#fdr_info) | [**Enrichment (N, B, n, b)**](http://cbl-gorilla.cs.technion.ac.il/GOrilla/v9ffx2p8/GOResultsFUNCTION.html#enrich_info) |
| --- | --- | --- | --- | --- |
| [GO:0008270](http://www.godatabase.org/cgi-bin/amigo/go.cgi?query=GO:0008270&view=details) | zinc ion binding | 6.62E-68 | 1.21E-64 | 2.32 (5033,998,744,342) |
| [GO:0046914](http://www.godatabase.org/cgi-bin/amigo/go.cgi?query=GO:0046914&view=details) | transition metal ion binding | 5.77E-62 | 5.26E-59 | 2.22 (5033,1043,744,342) |
| [GO:0003677](http://www.godatabase.org/cgi-bin/amigo/go.cgi?query=GO:0003677&view=details) | DNA binding | 2.41E-50 | 1.46E-47 | 1.98 (5033,1219,743,356) |
| [GO:0003676](http://www.godatabase.org/cgi-bin/amigo/go.cgi?query=GO:0003676&view=details) | nucleic acid binding | 6.17E-50 | 2.81E-47 | 1.66 (5033,1593,992,521) |
| [GO:0046872](http://www.godatabase.org/cgi-bin/amigo/go.cgi?query=GO:0046872&view=details) | metal ion binding | 7.87E-40 | 2.87E-37 | 1.76 (5033,1512,705,373) |
| [GO:0043169](http://www.godatabase.org/cgi-bin/amigo/go.cgi?query=GO:0043169&view=details) | cation binding | 2.87E-39 | 8.72E-37 | 1.72 (5033,1521,753,392) |
| [GO:0043167](http://www.godatabase.org/cgi-bin/amigo/go.cgi?query=GO:0043167&view=details) | ion binding | 3.47E-39 | 9.04E-37 | 1.72 (5033,1522,753,392) |
| [GO:0005201](http://www.godatabase.org/cgi-bin/amigo/go.cgi?query=GO:0005201&view=details) | extracellular matrix structural constituent | 6.86E-26 | 1.56E-23 | 9.29 (5033,51,361,34) |
| [GO:1901363](http://www.godatabase.org/cgi-bin/amigo/go.cgi?query=GO:1901363&view=details) | heterocyclic compound binding | 7.33E-23 | 1.49E-20 | 1.36 (5033,2062,992,554) |
| [GO:0097159](http://www.godatabase.org/cgi-bin/amigo/go.cgi?query=GO:0097159&view=details) | organic cyclic compound binding | 7.33E-23 | 1.34E-20 | 1.36 (5033,2062,992,554) |
| [GO:0005198](http://www.godatabase.org/cgi-bin/amigo/go.cgi?query=GO:0005198&view=details) | structural molecule activity | 1.71E-15 | 2.84E-13 | 6.25 (5033,206,125,32) |
| [GO:0048407](http://www.godatabase.org/cgi-bin/amigo/go.cgi?query=GO:0048407&view=details) | platelet-derived growth factor binding | 2.81E-8 | 4.27E-6 | 30.20 (5033,8,125,6) |

Component


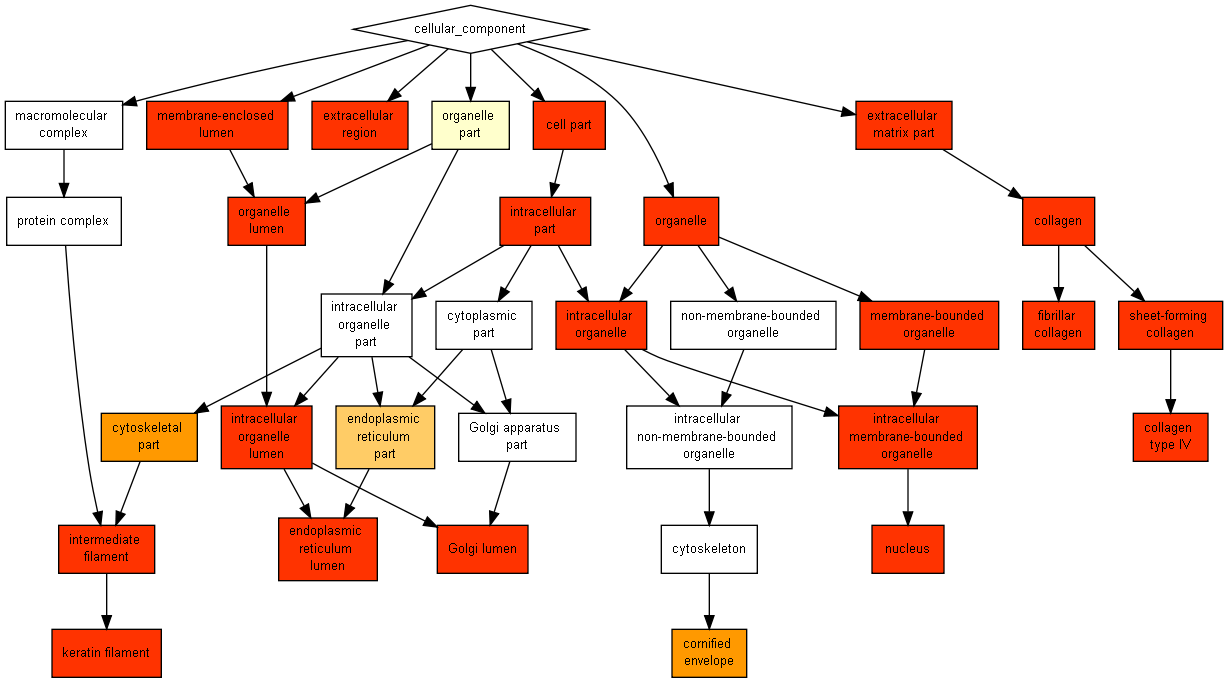


| **GO term** | **Description** | [**P-value**](http://cbl-gorilla.cs.technion.ac.il/GOrilla/v9ffx2p8/GOResultsCOMPONENT.html#p_value_info) | [**FDR q-value**](http://cbl-gorilla.cs.technion.ac.il/GOrilla/v9ffx2p8/GOResultsCOMPONENT.html#fdr_info) | [**Enrichment (N, B, n, b)**](http://cbl-gorilla.cs.technion.ac.il/GOrilla/v9ffx2p8/GOResultsCOMPONENT.html#enrich_info) |
| --- | --- | --- | --- | --- |
| [GO:0005882](http://www.godatabase.org/cgi-bin/amigo/go.cgi?query=GO:0005882&view=details) | intermediate filament | 1.36E-35 | 1.32E-32 | 5.32 (5033,106,607,68) |
| [GO:0005634](http://www.godatabase.org/cgi-bin/amigo/go.cgi?query=GO:0005634&view=details) | nucleus | 2.77E-33 | 1.34E-30 | 1.45 (5033,1976,996,567) |
| [GO:0045095](http://www.godatabase.org/cgi-bin/amigo/go.cgi?query=GO:0045095&view=details) | keratin filament | 4.93E-30 | 1.6E-27 | 6.62 (5033,70,521,48) |
| [GO:0044424](http://www.godatabase.org/cgi-bin/amigo/go.cgi?query=GO:0044424&view=details) | intracellular part | 1.42E-29 | 3.44E-27 | 1.18 (5033,3805,996,889) |
| [GO:0044464](http://www.godatabase.org/cgi-bin/amigo/go.cgi?query=GO:0044464&view=details) | cell part | 7.16E-25 | 1.39E-22 | 1.15 (5033,3968,996,904) |
| [GO:0070013](http://www.godatabase.org/cgi-bin/amigo/go.cgi?query=GO:0070013&view=details) | intracellular organelle lumen | 5.9E-24 | 9.55E-22 | 6.39 (5033,115,308,45) |
| [GO:0043233](http://www.godatabase.org/cgi-bin/amigo/go.cgi?query=GO:0043233&view=details) | organelle lumen | 5.91E-21 | 8.19E-19 | 5.57 (5033,132,308,45) |
| [GO:0031974](http://www.godatabase.org/cgi-bin/amigo/go.cgi?query=GO:0031974&view=details) | membrane-enclosed lumen | 1.75E-20 | 2.13E-18 | 5.45 (5033,135,308,45) |
| [GO:0005581](http://www.godatabase.org/cgi-bin/amigo/go.cgi?query=GO:0005581&view=details) | collagen | 3.44E-19 | 3.71E-17 | 7.68 (5033,66,308,31) |
| [GO:0005788](http://www.godatabase.org/cgi-bin/amigo/go.cgi?query=GO:0005788&view=details) | endoplasmic reticulum lumen | 2.85E-18 | 2.77E-16 | 7.54 (5033,65,308,30) |
| [GO:0043231](http://www.godatabase.org/cgi-bin/amigo/go.cgi?query=GO:0043231&view=details) | intracellular membrane-bounded organelle | 1.14E-17 | 1E-15 | 1.27 (5033,2437,999,616) |
| [GO:0043227](http://www.godatabase.org/cgi-bin/amigo/go.cgi?query=GO:0043227&view=details) | membrane-bounded organelle | 1.3E-17 | 1.05E-15 | 1.27 (5033,2438,999,616) |
| [GO:0043229](http://www.godatabase.org/cgi-bin/amigo/go.cgi?query=GO:0043229&view=details) | intracellular organelle | 5.09E-17 | 3.8E-15 | 1.23 (5033,2780,992,676) |
| [GO:0043226](http://www.godatabase.org/cgi-bin/amigo/go.cgi?query=GO:0043226&view=details) | organelle | 8.5E-17 | 5.9E-15 | 1.23 (5033,2784,992,676) |
| [GO:0044420](http://www.godatabase.org/cgi-bin/amigo/go.cgi?query=GO:0044420&view=details) | extracellular matrix part | 9.19E-16 | 5.95E-14 | 5.28 (5033,106,324,36) |
| [GO:0005583](http://www.godatabase.org/cgi-bin/amigo/go.cgi?query=GO:0005583&view=details) | fibrillar collagen | 1.91E-14 | 1.16E-12 | 30.71 (5033,11,149,10) |
| [GO:0005576](http://www.godatabase.org/cgi-bin/amigo/go.cgi?query=GO:0005576&view=details) | extracellular region | 3.32E-12 | 1.9E-10 | 4.31 (5033,301,128,33) |
| [GO:0005587](http://www.godatabase.org/cgi-bin/amigo/go.cgi?query=GO:0005587&view=details) | collagen type IV | 4.42E-11 | 2.38E-9 | 59.92 (5033,6,84,6) |
| [GO:0030935](http://www.godatabase.org/cgi-bin/amigo/go.cgi?query=GO:0030935&view=details) | sheet-forming collagen | 1.08E-10 | 5.52E-9 | 30.88 (5033,7,163,7) |
| [GO:0005796](http://www.godatabase.org/cgi-bin/amigo/go.cgi?query=GO:0005796&view=details) | Golgi lumen | 7.02E-10 | 3.41E-8 | 18.27 (5033,29,95,10) |
| [GO:0044430](http://www.godatabase.org/cgi-bin/amigo/go.cgi?query=GO:0044430&view=details) | cytoskeletal part | 2.02E-8 | 9.35E-7 | 2.59 (5033,531,161,44) |
| [GO:0001533](http://www.godatabase.org/cgi-bin/amigo/go.cgi?query=GO:0001533&view=details) | cornified envelope | 7.5E-8 | 3.31E-6 | 5.00 (5033,15,873,13) |
| [GO:0044432](http://www.godatabase.org/cgi-bin/amigo/go.cgi?query=GO:0044432&view=details) | endoplasmic reticulum part | 2.79E-7 | 1.18E-5 | 3.50 (5033,208,173,25) |

**3) RP - Ranked list test of** 5511 human compositionally ordered proteins, with p-value threshold of 10^-6^ yields:

Process


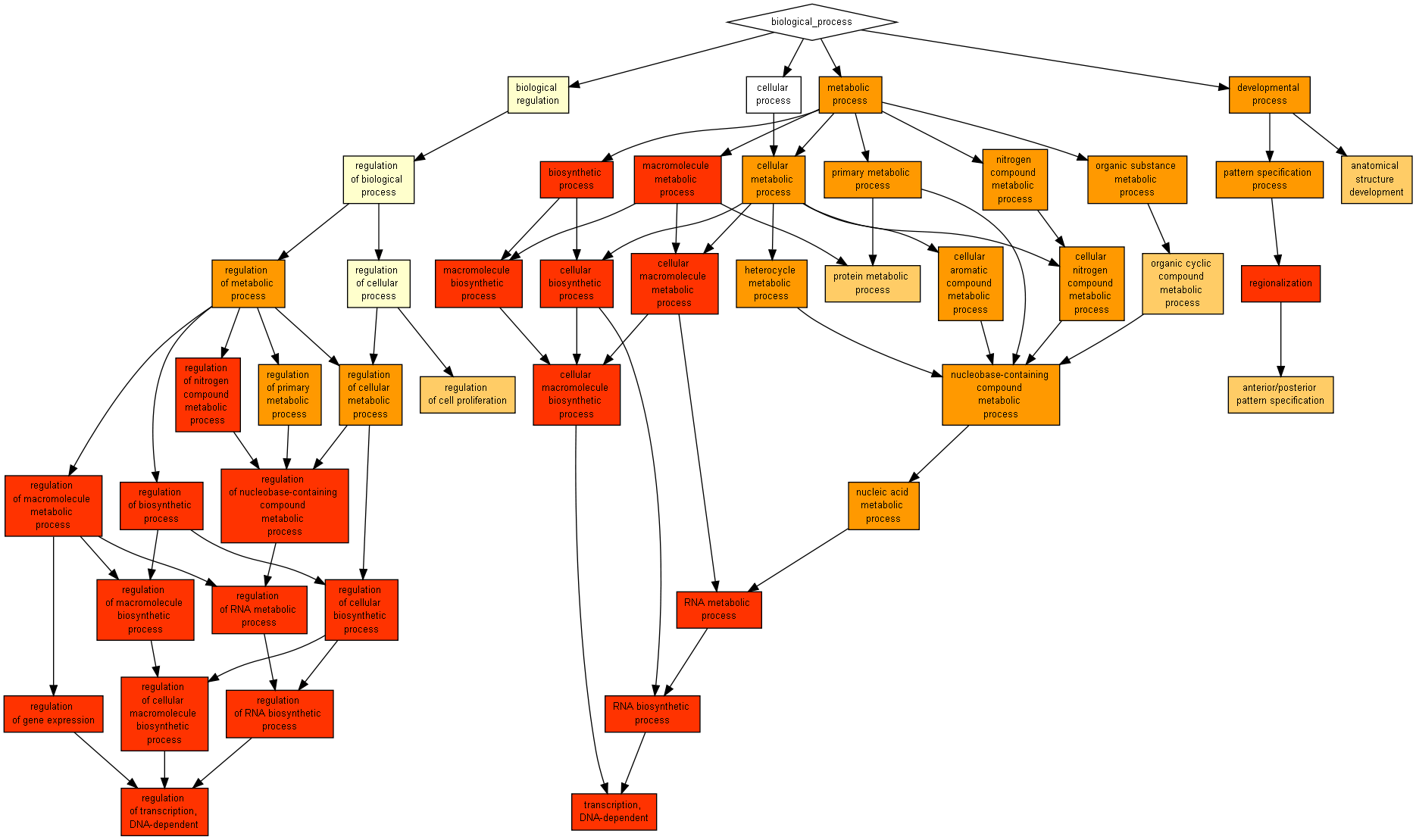


| **GO term** | **Description** | [**P-value**](http://cbl-gorilla.cs.technion.ac.il/GOrilla/hqe91pzt/GOResultsPROCESS.html#p_value_info) | [**FDR q-value**](http://cbl-gorilla.cs.technion.ac.il/GOrilla/hqe91pzt/GOResultsPROCESS.html#fdr_info) | [**Enrichment (N, B, n, b)**](http://cbl-gorilla.cs.technion.ac.il/GOrilla/hqe91pzt/GOResultsPROCESS.html#enrich_info) |
| --- | --- | --- | --- | --- |
| [GO:0032774](http://www.godatabase.org/cgi-bin/amigo/go.cgi?query=GO:0032774&view=details) | RNA biosynthetic process | 3.01E-14 | 2.23E-10 | 1.47 (5033,1187,897,312) |
| [GO:0006351](http://www.godatabase.org/cgi-bin/amigo/go.cgi?query=GO:0006351&view=details) | transcription, DNA-dependent | 1.52E-13 | 5.64E-10 | 1.48 (5033,1140,897,300) |
| [GO:0009058](http://www.godatabase.org/cgi-bin/amigo/go.cgi?query=GO:0009058&view=details) | biosynthetic process | 2.42E-13 | 5.99E-10 | 1.39 (5033,1390,897,344) |
| [GO:0051252](http://www.godatabase.org/cgi-bin/amigo/go.cgi?query=GO:0051252&view=details) | regulation of RNA metabolic process | 5.11E-13 | 9.47E-10 | 1.39 (5033,1356,900,337) |
| [GO:0034645](http://www.godatabase.org/cgi-bin/amigo/go.cgi?query=GO:0034645&view=details) | cellular macromolecule biosynthetic process | 5.7E-13 | 8.46E-10 | 1.45 (5033,1223,897,315) |
| [GO:0006355](http://www.godatabase.org/cgi-bin/amigo/go.cgi?query=GO:0006355&view=details) | regulation of transcription, DNA-dependent | 7.57E-13 | 9.35E-10 | 1.39 (5033,1319,900,329) |
| [GO:2001141](http://www.godatabase.org/cgi-bin/amigo/go.cgi?query=GO:2001141&view=details) | regulation of RNA biosynthetic process | 1.07E-12 | 1.13E-9 | 1.39 (5033,1322,900,329) |
| [GO:0009059](http://www.godatabase.org/cgi-bin/amigo/go.cgi?query=GO:0009059&view=details) | macromolecule biosynthetic process | 1.11E-12 | 1.03E-9 | 1.44 (5033,1228,897,315) |
| [GO:0044249](http://www.godatabase.org/cgi-bin/amigo/go.cgi?query=GO:0044249&view=details) | cellular biosynthetic process | 1.19E-12 | 9.77E-10 | 1.41 (5033,1368,897,343) |
| [GO:0031326](http://www.godatabase.org/cgi-bin/amigo/go.cgi?query=GO:0031326&view=details) | regulation of cellular biosynthetic process | 1.04E-11 | 7.68E-9 | 1.35 (5033,1433,900,347) |
| [GO:0043170](http://www.godatabase.org/cgi-bin/amigo/go.cgi?query=GO:0043170&view=details) | macromolecule metabolic process | 1.43E-11 | 9.62E-9 | 1.26 (5033,2114,897,474) |
| [GO:0009889](http://www.godatabase.org/cgi-bin/amigo/go.cgi?query=GO:0009889&view=details) | regulation of biosynthetic process | 1.8E-11 | 1.11E-8 | 1.35 (5033,1438,900,347) |
| [GO:0010556](http://www.godatabase.org/cgi-bin/amigo/go.cgi?query=GO:0010556&view=details) | regulation of macromolecule biosynthetic process | 2.78E-11 | 1.59E-8 | 1.35 (5033,1406,900,340) |
| [GO:2000112](http://www.godatabase.org/cgi-bin/amigo/go.cgi?query=GO:2000112&view=details) | regulation of cellular macromolecule biosynthetic process | 3.06E-11 | 1.62E-8 | 1.35 (5033,1392,900,337) |
| [GO:0016070](http://www.godatabase.org/cgi-bin/amigo/go.cgi?query=GO:0016070&view=details) | RNA metabolic process | 3.37E-11 | 1.66E-8 | 1.35 (5033,1423,897,342) |
| [GO:0010468](http://www.godatabase.org/cgi-bin/amigo/go.cgi?query=GO:0010468&view=details) | regulation of gene expression | 3.52E-11 | 1.63E-8 | 1.34 (5033,1470,900,352) |
| [GO:0019219](http://www.godatabase.org/cgi-bin/amigo/go.cgi?query=GO:0019219&view=details) | regulation of nucleobase-containing compound metabolic process | 6.01E-11 | 2.62E-8 | 1.33 (5033,1480,900,353) |
| [GO:0051171](http://www.godatabase.org/cgi-bin/amigo/go.cgi?query=GO:0051171&view=details) | regulation of nitrogen compound metabolic process | 1.83E-10 | 7.54E-8 | 1.32 (5033,1491,900,353) |
| [GO:0060255](http://www.godatabase.org/cgi-bin/amigo/go.cgi?query=GO:0060255&view=details) | regulation of macromolecule metabolic process | 3.27E-10 | 1.28E-7 | 1.29 (5033,1669,897,385) |
| [GO:0003002](http://www.godatabase.org/cgi-bin/amigo/go.cgi?query=GO:0003002&view=details) | regionalization | 6.16E-10 | 2.28E-7 | 3.48 (5033,110,447,34) |
| [GO:0044260](http://www.godatabase.org/cgi-bin/amigo/go.cgi?query=GO:0044260&view=details) | cellular macromolecule metabolic process | 6.22E-10 | 2.19E-7 | 1.25 (5033,2013,897,448) |
| [GO:0044238](http://www.godatabase.org/cgi-bin/amigo/go.cgi?query=GO:0044238&view=details) | primary metabolic process | 1.08E-9 | 3.64E-7 | 1.22 (5033,2288,897,497) |
| [GO:0090304](http://www.godatabase.org/cgi-bin/amigo/go.cgi?query=GO:0090304&view=details) | nucleic acid metabolic process | 3.69E-9 | 1.19E-6 | 1.29 (5033,1558,897,359) |
| [GO:0080090](http://www.godatabase.org/cgi-bin/amigo/go.cgi?query=GO:0080090&view=details) | regulation of primary metabolic process | 7.33E-9 | 2.26E-6 | 1.27 (5033,1697,897,384) |
| [GO:0032502](http://www.godatabase.org/cgi-bin/amigo/go.cgi?query=GO:0032502&view=details) | developmental process | 7.97E-9 | 2.36E-6 | 1.54 (5033,1228,412,155) |
| [GO:0031323](http://www.godatabase.org/cgi-bin/amigo/go.cgi?query=GO:0031323&view=details) | regulation of cellular metabolic process | 1.46E-8 | 4.17E-6 | 1.26 (5033,1737,897,390) |
| [GO:0007389](http://www.godatabase.org/cgi-bin/amigo/go.cgi?query=GO:0007389&view=details) | pattern specification process | 2.1E-8 | 5.78E-6 | 2.75 (5033,168,447,41) |
| [GO:0008152](http://www.godatabase.org/cgi-bin/amigo/go.cgi?query=GO:0008152&view=details) | metabolic process | 2.17E-8 | 5.75E-6 | 1.20 (5033,2380,897,507) |
| [GO:0034641](http://www.godatabase.org/cgi-bin/amigo/go.cgi?query=GO:0034641&view=details) | cellular nitrogen compound metabolic process | 2.71E-8 | 6.92E-6 | 1.27 (5033,1643,897,371) |
| [GO:0019222](http://www.godatabase.org/cgi-bin/amigo/go.cgi?query=GO:0019222&view=details) | regulation of metabolic process | 2.95E-8 | 7.28E-6 | 1.24 (5033,1836,897,407) |
| [GO:0006807](http://www.godatabase.org/cgi-bin/amigo/go.cgi?query=GO:0006807&view=details) | nitrogen compound metabolic process | 3.13E-8 | 7.48E-6 | 1.27 (5033,1645,897,371) |
| [GO:0071704](http://www.godatabase.org/cgi-bin/amigo/go.cgi?query=GO:0071704&view=details) | organic substance metabolic process | 3.17E-8 | 7.35E-6 | 1.26 (5033,1719,897,385) |
| [GO:0006139](http://www.godatabase.org/cgi-bin/amigo/go.cgi?query=GO:0006139&view=details) | nucleobase-containing compound metabolic process | 4.34E-8 | 9.76E-6 | 1.27 (5033,1617,897,365) |
| [GO:0044237](http://www.godatabase.org/cgi-bin/amigo/go.cgi?query=GO:0044237&view=details) | cellular metabolic process | 5.04E-8 | 1.1E-5 | 1.20 (5033,2224,897,477) |
| [GO:0006725](http://www.godatabase.org/cgi-bin/amigo/go.cgi?query=GO:0006725&view=details) | cellular aromatic compound metabolic process | 6.13E-8 | 1.3E-5 | 1.26 (5033,1637,897,368) |
| [GO:0046483](http://www.godatabase.org/cgi-bin/amigo/go.cgi?query=GO:0046483&view=details) | heterocycle metabolic process | 8.38E-8 | 1.72E-5 | 1.26 (5033,1630,897,366) |
| [GO:1901360](http://www.godatabase.org/cgi-bin/amigo/go.cgi?query=GO:1901360&view=details) | organic cyclic compound metabolic process | 1.44E-7 | 2.89E-5 | 1.25 (5033,1663,897,371) |
| [GO:0048856](http://www.godatabase.org/cgi-bin/amigo/go.cgi?query=GO:0048856&view=details) | anatomical structure development | 4.36E-7 | 8.5E-5 | 1.64 (5033,770,426,107) |
| [GO:0019538](http://www.godatabase.org/cgi-bin/amigo/go.cgi?query=GO:0019538&view=details) | protein metabolic process | 5.5E-7 | 1.05E-4 | 1.79 (5033,713,319,81) |
| [GO:0042127](http://www.godatabase.org/cgi-bin/amigo/go.cgi?query=GO:0042127&view=details) | regulation of cell proliferation | 6.8E-7 | 1.26E-4 | 1.92 (5033,298,581,66) |
| [GO:0009952](http://www.godatabase.org/cgi-bin/amigo/go.cgi?query=GO:0009952&view=details) | anterior/posterior pattern specification | 8.16E-7 | 1.48E-4 | 3.59 (5033,69,447,22) |

Function


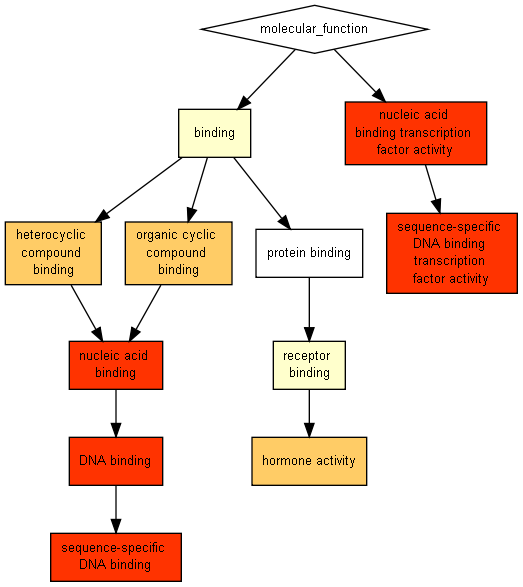


| **GO term** | **Description** | [**P-value**](http://cbl-gorilla.cs.technion.ac.il/GOrilla/hqe91pzt/GOResultsFUNCTION.html#p_value_info) | [**FDR q-value**](http://cbl-gorilla.cs.technion.ac.il/GOrilla/hqe91pzt/GOResultsFUNCTION.html#fdr_info) | [**Enrichment (N, B, n, b)**](http://cbl-gorilla.cs.technion.ac.il/GOrilla/hqe91pzt/GOResultsFUNCTION.html#enrich_info) |
| --- | --- | --- | --- | --- |
| [GO:0003676](http://www.godatabase.org/cgi-bin/amigo/go.cgi?query=GO:0003676&view=details) | nucleic acid binding | 3.97E-15 | 7.24E-12 | 1.39 (5033,1593,897,396) |
| [GO:0003677](http://www.godatabase.org/cgi-bin/amigo/go.cgi?query=GO:0003677&view=details) | DNA binding | 5.44E-15 | 4.96E-12 | 1.48 (5033,1219,897,321) |
| [GO:0043565](http://www.godatabase.org/cgi-bin/amigo/go.cgi?query=GO:0043565&view=details) | sequence-specific DNA binding | 2.36E-14 | 1.44E-11 | 2.39 (5033,299,591,84) |
| [GO:0001071](http://www.godatabase.org/cgi-bin/amigo/go.cgi?query=GO:0001071&view=details) | nucleic acid binding transcription factor activity | 1.68E-13 | 7.66E-11 | 1.85 (5033,455,879,147) |
| [GO:0003700](http://www.godatabase.org/cgi-bin/amigo/go.cgi?query=GO:0003700&view=details) | sequence-specific DNA binding transcription factor activity | 1.68E-13 | 6.13E-11 | 1.85 (5033,455,879,147) |
| [GO:1901363](http://www.godatabase.org/cgi-bin/amigo/go.cgi?query=GO:1901363&view=details) | heterocyclic compound binding | 2.34E-7 | 7.12E-5 | 1.21 (5033,2062,897,444) |
| [GO:0097159](http://www.godatabase.org/cgi-bin/amigo/go.cgi?query=GO:0097159&view=details) | organic cyclic compound binding | 2.34E-7 | 6.11E-5 | 1.21 (5033,2062,897,444) |
| [GO:0005179](http://www.godatabase.org/cgi-bin/amigo/go.cgi?query=GO:0005179&view=details) | hormone activity | 4.66E-7 | 1.06E-4 | 10.15 (5033,18,248,9) |

Component


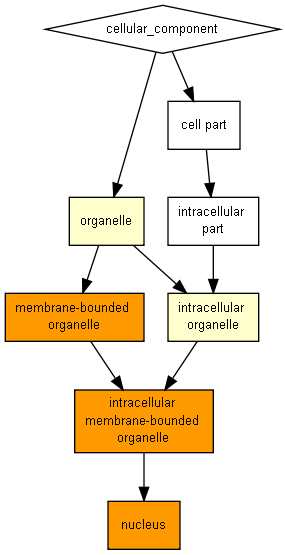


| **GO term** | **Description** | [**P-value**](http://cbl-gorilla.cs.technion.ac.il/GOrilla/hqe91pzt/GOResultsCOMPONENT.html#p_value_info) | [**FDR q-value**](http://cbl-gorilla.cs.technion.ac.il/GOrilla/hqe91pzt/GOResultsCOMPONENT.html#fdr_info) | [**Enrichment (N, B, n, b)**](http://cbl-gorilla.cs.technion.ac.il/GOrilla/hqe91pzt/GOResultsCOMPONENT.html#enrich_info) |
| --- | --- | --- | --- | --- |
| [GO:0005634](http://www.godatabase.org/cgi-bin/amigo/go.cgi?query=GO:0005634&view=details) | nucleus | 4.7E-9 | 4.56E-6 | 1.24 (5033,1976,897,437) |
| [GO:0043231](http://www.godatabase.org/cgi-bin/amigo/go.cgi?query=GO:0043231&view=details) | intracellular membrane-bounded organelle | 8.67E-8 | 4.21E-5 | 1.18 (5033,2437,897,514) |
| [GO:0043227](http://www.godatabase.org/cgi-bin/amigo/go.cgi?query=GO:0043227&view=details) | membrane-bounded organelle | 9.36E-8 | 3.03E-5 | 1.18 (5033,2438,897,514) |
