# Supplementary material for: Systematic Analysis of Compositional Order of Proteins Reveals New Characteristics of Biological Functions and a Universal Correlate of Macroevolution
Source: PLoS Comput Biol. 2013 Nov 21;9(11):e1003346. doi: 10.1371/journal.pcbi.1003346 (PMC3836704; doi:10.1371/journal.pcbi.1003346)
Supplement: Text S3 — GOrilla analysis of Human novel CO proteins. Results of functional enrichments in Human novel CO proteins using GOrilla web-tool. Enrichments of processes, functions and cellular components are shown for (i) Human novel CO proteins ranked by RC (ii) Human novel CO proteins ranked by RP. (DOCX) [file pcbi.1003346.s028.docx]

**Supporting Information – Text S2**

Analysis of human CO novel protein (n=1368) by GOrilla [Eden et al., 2007], for lists ranked by RC (1) and RP (2).

**1) RC - Ranked list test** with p-value threshold of 10^-6^ yields:

Process


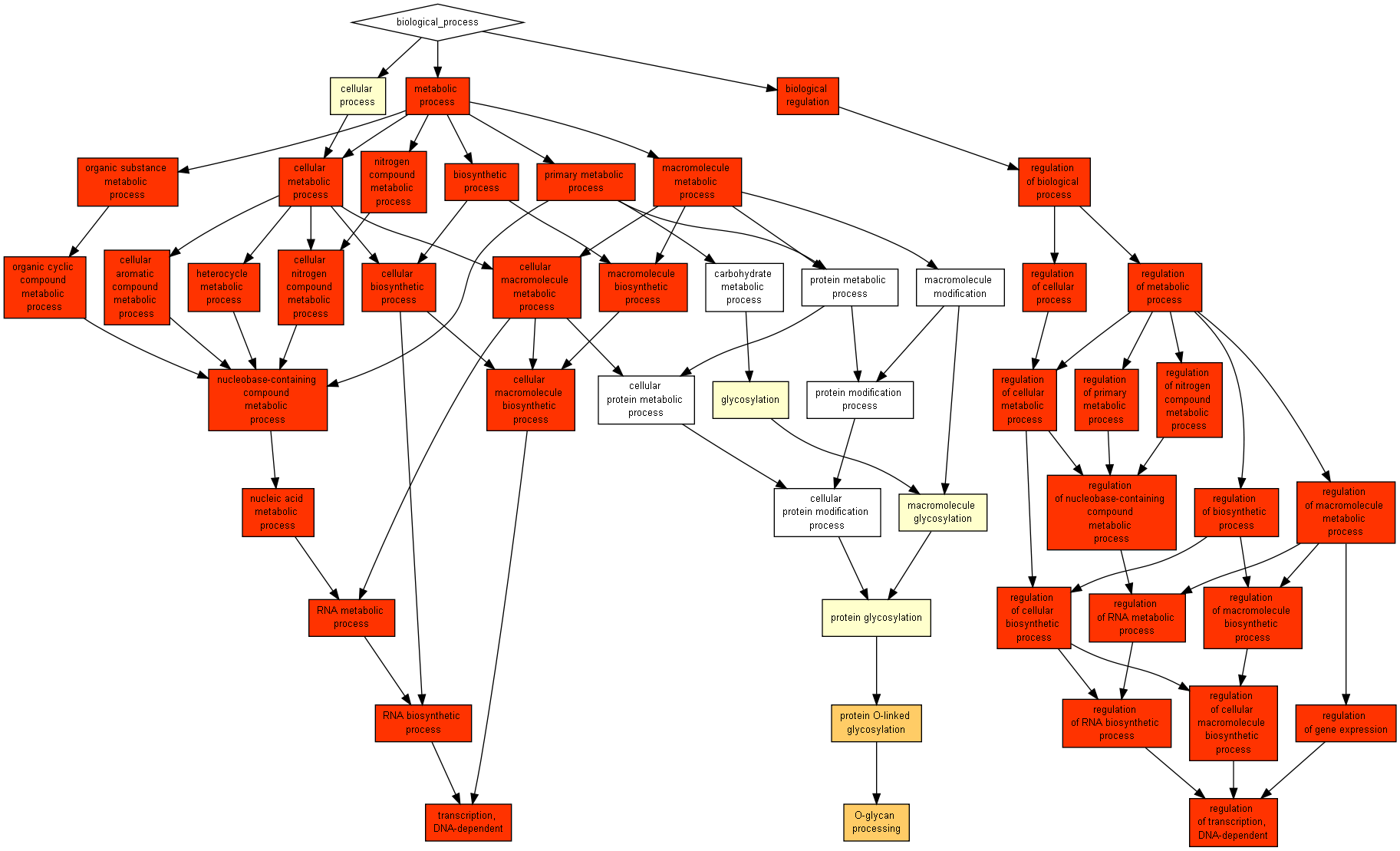


| **GO term** | **Description** | [**P-value**](http://cbl-gorilla.cs.technion.ac.il/GOrilla/r5dlbln3/GOResultsPROCESS.html#p_value_info) | [**FDR q-value**](http://cbl-gorilla.cs.technion.ac.il/GOrilla/r5dlbln3/GOResultsPROCESS.html#fdr_info) | [**Enrichment (N, B, n, b)**](http://cbl-gorilla.cs.technion.ac.il/GOrilla/r5dlbln3/GOResultsPROCESS.html#enrich_info) |
| --- | --- | --- | --- | --- |
| [GO:0032774](http://www.godatabase.org/cgi-bin/amigo/go.cgi?query=GO:0032774&view=details) | RNA biosynthetic process | 2.75E-50 | 5.96E-47 | 1.46 (1139,425,680,371) |
| [GO:0006351](http://www.godatabase.org/cgi-bin/amigo/go.cgi?query=GO:0006351&view=details) | transcription, DNA-dependent | 1E-49 | 1.09E-46 | 1.46 (1139,420,680,367) |
| [GO:0034645](http://www.godatabase.org/cgi-bin/amigo/go.cgi?query=GO:0034645&view=details) | cellular macromolecule biosynthetic process | 3.92E-47 | 2.83E-44 | 1.45 (1139,430,680,371) |
| [GO:0009059](http://www.godatabase.org/cgi-bin/amigo/go.cgi?query=GO:0009059&view=details) | macromolecule biosynthetic process | 3.92E-47 | 2.12E-44 | 1.45 (1139,430,680,371) |
| [GO:0016070](http://www.godatabase.org/cgi-bin/amigo/go.cgi?query=GO:0016070&view=details) | RNA metabolic process | 8.73E-47 | 3.78E-44 | 1.43 (1139,453,680,386) |
| [GO:0044249](http://www.godatabase.org/cgi-bin/amigo/go.cgi?query=GO:0044249&view=details) | cellular biosynthetic process | 5.17E-45 | 1.86E-42 | 1.43 (1139,441,680,376) |
| [GO:0009058](http://www.godatabase.org/cgi-bin/amigo/go.cgi?query=GO:0009058&view=details) | biosynthetic process | 1.96E-44 | 6.07E-42 | 1.42 (1139,442,680,376) |
| [GO:0006355](http://www.godatabase.org/cgi-bin/amigo/go.cgi?query=GO:0006355&view=details) | regulation of transcription, DNA-dependent | 5.37E-44 | 1.45E-41 | 1.44 (1139,413,680,356) |
| [GO:2001141](http://www.godatabase.org/cgi-bin/amigo/go.cgi?query=GO:2001141&view=details) | regulation of RNA biosynthetic process | 5.37E-44 | 1.29E-41 | 1.44 (1139,413,680,356) |
| [GO:0051252](http://www.godatabase.org/cgi-bin/amigo/go.cgi?query=GO:0051252&view=details) | regulation of RNA metabolic process | 1.11E-43 | 2.41E-41 | 1.44 (1139,415,680,357) |
| [GO:2000112](http://www.godatabase.org/cgi-bin/amigo/go.cgi?query=GO:2000112&view=details) | regulation of cellular macromolecule biosynthetic process | 1.18E-43 | 2.33E-41 | 1.44 (1139,418,680,359) |
| [GO:0010556](http://www.godatabase.org/cgi-bin/amigo/go.cgi?query=GO:0010556&view=details) | regulation of macromolecule biosynthetic process | 4.44E-43 | 8.02E-41 | 1.61 (1139,419,504,299) |
| [GO:0009889](http://www.godatabase.org/cgi-bin/amigo/go.cgi?query=GO:0009889&view=details) | regulation of biosynthetic process | 1.1E-42 | 1.84E-40 | 1.61 (1139,422,504,300) |
| [GO:0031326](http://www.godatabase.org/cgi-bin/amigo/go.cgi?query=GO:0031326&view=details) | regulation of cellular biosynthetic process | 2.74E-42 | 4.25E-40 | 1.61 (1139,421,504,299) |
| [GO:0010468](http://www.godatabase.org/cgi-bin/amigo/go.cgi?query=GO:0010468&view=details) | regulation of gene expression | 7.04E-42 | 1.02E-39 | 1.42 (1139,427,680,363) |
| [GO:0090304](http://www.godatabase.org/cgi-bin/amigo/go.cgi?query=GO:0090304&view=details) | nucleic acid metabolic process | 1.61E-41 | 2.18E-39 | 1.40 (1139,467,680,389) |
| [GO:0006139](http://www.godatabase.org/cgi-bin/amigo/go.cgi?query=GO:0006139&view=details) | nucleobase-containing compound metabolic process | 1.49E-40 | 1.9E-38 | 1.39 (1139,475,680,393) |
| [GO:0019219](http://www.godatabase.org/cgi-bin/amigo/go.cgi?query=GO:0019219&view=details) | regulation of nucleobase-containing compound metabolic process | 5.67E-40 | 6.82E-38 | 1.58 (1139,425,504,298) |
| [GO:0051171](http://www.godatabase.org/cgi-bin/amigo/go.cgi?query=GO:0051171&view=details) | regulation of nitrogen compound metabolic process | 5.67E-40 | 6.46E-38 | 1.58 (1139,425,504,298) |
| [GO:0034641](http://www.godatabase.org/cgi-bin/amigo/go.cgi?query=GO:0034641&view=details) | cellular nitrogen compound metabolic process | 1.58E-39 | 1.71E-37 | 1.38 (1139,477,680,393) |
| [GO:0006725](http://www.godatabase.org/cgi-bin/amigo/go.cgi?query=GO:0006725&view=details) | cellular aromatic compound metabolic process | 5.05E-39 | 5.21E-37 | 1.38 (1139,478,680,393) |
| [GO:0006807](http://www.godatabase.org/cgi-bin/amigo/go.cgi?query=GO:0006807&view=details) | nitrogen compound metabolic process | 5.05E-39 | 4.98E-37 | 1.38 (1139,478,680,393) |
| [GO:0046483](http://www.godatabase.org/cgi-bin/amigo/go.cgi?query=GO:0046483&view=details) | heterocycle metabolic process | 5.05E-39 | 4.76E-37 | 1.38 (1139,478,680,393) |
| [GO:0060255](http://www.godatabase.org/cgi-bin/amigo/go.cgi?query=GO:0060255&view=details) | regulation of macromolecule metabolic process | 6.89E-39 | 6.21E-37 | 1.40 (1139,443,680,370) |
| [GO:1901360](http://www.godatabase.org/cgi-bin/amigo/go.cgi?query=GO:1901360&view=details) | organic cyclic compound metabolic process | 8.49E-39 | 7.35E-37 | 1.37 (1139,480,680,394) |
| [GO:0080090](http://www.godatabase.org/cgi-bin/amigo/go.cgi?query=GO:0080090&view=details) | regulation of primary metabolic process | 1.61E-37 | 1.34E-35 | 1.55 (1139,446,504,305) |
| [GO:0071704](http://www.godatabase.org/cgi-bin/amigo/go.cgi?query=GO:0071704&view=details) | organic substance metabolic process | 7.03E-36 | 5.64E-34 | 1.36 (1139,486,680,394) |
| [GO:0031323](http://www.godatabase.org/cgi-bin/amigo/go.cgi?query=GO:0031323&view=details) | regulation of cellular metabolic process | 2.19E-35 | 1.7E-33 | 1.53 (1139,448,504,303) |
| [GO:0019222](http://www.godatabase.org/cgi-bin/amigo/go.cgi?query=GO:0019222&view=details) | regulation of metabolic process | 6.08E-34 | 4.54E-32 | 1.59 (1139,463,421,272) |
| [GO:0044260](http://www.godatabase.org/cgi-bin/amigo/go.cgi?query=GO:0044260&view=details) | cellular macromolecule metabolic process | 9.42E-31 | 6.8E-29 | 1.31 (1139,528,682,414) |
| [GO:0043170](http://www.godatabase.org/cgi-bin/amigo/go.cgi?query=GO:0043170&view=details) | macromolecule metabolic process | 3.07E-27 | 2.14E-25 | 1.29 (1139,540,682,416) |
| [GO:0044237](http://www.godatabase.org/cgi-bin/amigo/go.cgi?query=GO:0044237&view=details) | cellular metabolic process | 2.2E-23 | 1.49E-21 | 1.26 (1139,557,682,420) |
| [GO:0044238](http://www.godatabase.org/cgi-bin/amigo/go.cgi?query=GO:0044238&view=details) | primary metabolic process | 8.69E-23 | 5.7E-21 | 1.35 (1139,562,507,337) |
| [GO:0008152](http://www.godatabase.org/cgi-bin/amigo/go.cgi?query=GO:0008152&view=details) | metabolic process | 2.22E-19 | 1.42E-17 | 1.35 (1139,577,450,307) |
| [GO:0050794](http://www.godatabase.org/cgi-bin/amigo/go.cgi?query=GO:0050794&view=details) | regulation of cellular process | 8.66E-19 | 5.36E-17 | 1.37 (1139,556,422,283) |
| [GO:0050789](http://www.godatabase.org/cgi-bin/amigo/go.cgi?query=GO:0050789&view=details) | regulation of biological process | 1.09E-17 | 6.54E-16 | 1.35 (1139,570,422,286) |
| [GO:0065007](http://www.godatabase.org/cgi-bin/amigo/go.cgi?query=GO:0065007&view=details) | biological regulation | 5.31E-17 | 3.11E-15 | 1.34 (1139,582,422,289) |
| [GO:0016266](http://www.godatabase.org/cgi-bin/amigo/go.cgi?query=GO:0016266&view=details) | O-glycan processing | 5.41E-7 | 3.08E-5 | 13.07 (1139,10,61,7) |

Function


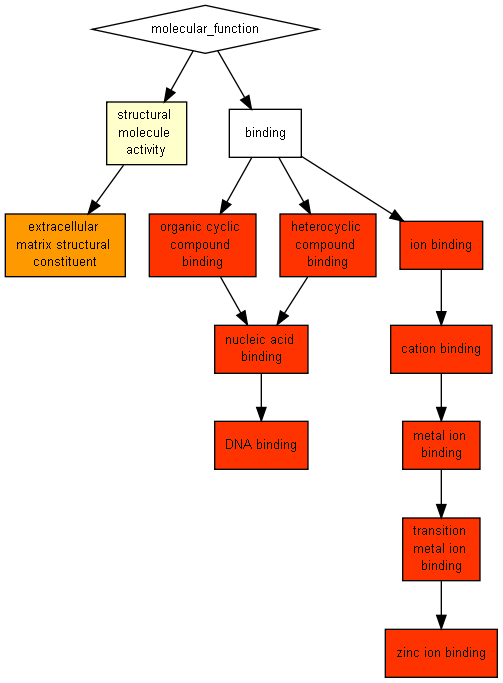


| **GO term** | **Description** | [**P-value**](http://cbl-gorilla.cs.technion.ac.il/GOrilla/r5dlbln3/GOResultsFUNCTION.html#p_value_info) | [**FDR q-value**](http://cbl-gorilla.cs.technion.ac.il/GOrilla/r5dlbln3/GOResultsFUNCTION.html#fdr_info) | [**Enrichment (N, B, n, b)**](http://cbl-gorilla.cs.technion.ac.il/GOrilla/r5dlbln3/GOResultsFUNCTION.html#enrich_info) |
| --- | --- | --- | --- | --- |
| [GO:0003677](http://www.godatabase.org/cgi-bin/amigo/go.cgi?query=GO:0003677&view=details) | DNA binding | 5.18E-44 | 3.2E-41 | 1.48 (1139,434,625,352) |
| [GO:0008270](http://www.godatabase.org/cgi-bin/amigo/go.cgi?query=GO:0008270&view=details) | zinc ion binding | 3.99E-42 | 1.23E-39 | 1.52 (1139,444,560,332) |
| [GO:0046914](http://www.godatabase.org/cgi-bin/amigo/go.cgi?query=GO:0046914&view=details) | transition metal ion binding | 8.01E-38 | 1.65E-35 | 1.54 (1139,455,507,311) |
| [GO:0003676](http://www.godatabase.org/cgi-bin/amigo/go.cgi?query=GO:0003676&view=details) | nucleic acid binding | 2.79E-35 | 4.31E-33 | 1.46 (1139,520,509,339) |
| [GO:1901363](http://www.godatabase.org/cgi-bin/amigo/go.cgi?query=GO:1901363&view=details) | heterocyclic compound binding | 8.49E-22 | 1.05E-19 | 1.26 (1139,592,621,408) |
| [GO:0097159](http://www.godatabase.org/cgi-bin/amigo/go.cgi?query=GO:0097159&view=details) | organic cyclic compound binding | 8.49E-22 | 8.73E-20 | 1.26 (1139,592,621,408) |
| [GO:0046872](http://www.godatabase.org/cgi-bin/amigo/go.cgi?query=GO:0046872&view=details) | metal ion binding | 1.87E-21 | 1.65E-19 | 1.39 (1139,538,445,293) |
| [GO:0043169](http://www.godatabase.org/cgi-bin/amigo/go.cgi?query=GO:0043169&view=details) | cation binding | 3.07E-21 | 2.37E-19 | 1.39 (1139,539,445,293) |
| [GO:0043167](http://www.godatabase.org/cgi-bin/amigo/go.cgi?query=GO:0043167&view=details) | ion binding | 3.07E-21 | 2.1E-19 | 1.39 (1139,539,445,293) |
| [GO:0005201](http://www.godatabase.org/cgi-bin/amigo/go.cgi?query=GO:0005201&view=details) | extracellular matrix structural constituent | 1.39E-8 | 8.57E-7 | 12.98 (1139,9,78,8) |

Component


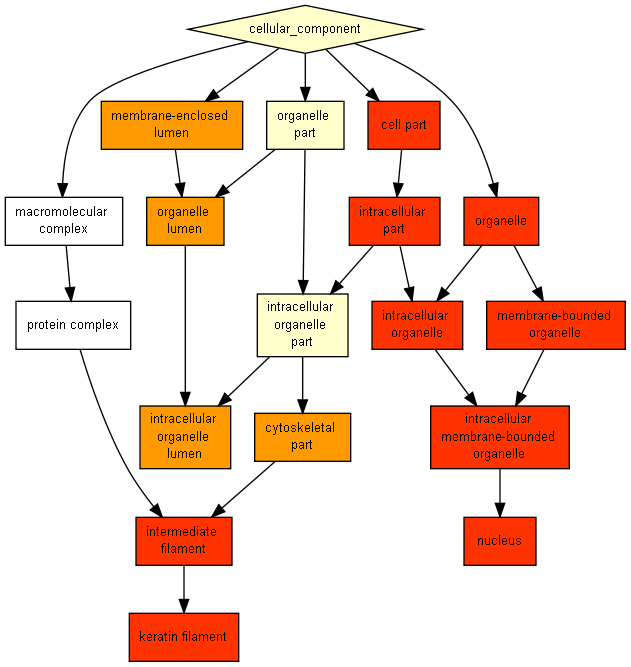


| **GO term** | **Description** | [**P-value**](http://cbl-gorilla.cs.technion.ac.il/GOrilla/r5dlbln3/GOResultsCOMPONENT.html#p_value_info) | [**FDR q-value**](http://cbl-gorilla.cs.technion.ac.il/GOrilla/r5dlbln3/GOResultsCOMPONENT.html#fdr_info) | [**Enrichment (N, B, n, b)**](http://cbl-gorilla.cs.technion.ac.il/GOrilla/r5dlbln3/GOResultsCOMPONENT.html#enrich_info) |
| --- | --- | --- | --- | --- |
| [GO:0044424](http://www.godatabase.org/cgi-bin/amigo/go.cgi?query=GO:0044424&view=details) | intracellular part | 2.91E-36 | 1.13E-33 | 1.19 (1139,830,680,592) |
| [GO:0044464](http://www.godatabase.org/cgi-bin/amigo/go.cgi?query=GO:0044464&view=details) | cell part | 1.34E-33 | 2.61E-31 | 1.20 (1139,863,577,526) |
| [GO:0005634](http://www.godatabase.org/cgi-bin/amigo/go.cgi?query=GO:0005634&view=details) | nucleus | 2.23E-33 | 2.9E-31 | 1.31 (1139,549,680,430) |
| [GO:0043231](http://www.godatabase.org/cgi-bin/amigo/go.cgi?query=GO:0043231&view=details) | intracellular membrane-bounded organelle | 1.41E-26 | 1.38E-24 | 1.26 (1139,601,680,451) |
| [GO:0043227](http://www.godatabase.org/cgi-bin/amigo/go.cgi?query=GO:0043227&view=details) | membrane-bounded organelle | 1.41E-26 | 1.1E-24 | 1.26 (1139,601,680,451) |
| [GO:0043226](http://www.godatabase.org/cgi-bin/amigo/go.cgi?query=GO:0043226&view=details) | organelle | 6.41E-26 | 4.16E-24 | 1.29 (1139,639,570,412) |
| [GO:0043229](http://www.godatabase.org/cgi-bin/amigo/go.cgi?query=GO:0043229&view=details) | intracellular organelle | 6.41E-26 | 3.57E-24 | 1.29 (1139,639,570,412) |
| [GO:0005882](http://www.godatabase.org/cgi-bin/amigo/go.cgi?query=GO:0005882&view=details) | intermediate filament | 3.52E-17 | 1.72E-15 | 2.62 (1139,59,383,52) |
| [GO:0045095](http://www.godatabase.org/cgi-bin/amigo/go.cgi?query=GO:0045095&view=details) | keratin filament | 8.59E-15 | 3.72E-13 | 4.26 (1139,46,186,32) |
| [GO:0044430](http://www.godatabase.org/cgi-bin/amigo/go.cgi?query=GO:0044430&view=details) | cytoskeletal part | 1.34E-8 | 5.21E-7 | 3.07 (1139,108,110,32) |
| [GO:0070013](http://www.godatabase.org/cgi-bin/amigo/go.cgi?query=GO:0070013&view=details) | intracellular organelle lumen | 2.24E-8 | 7.93E-7 | 7.97 (1139,22,78,12) |
| [GO:0043233](http://www.godatabase.org/cgi-bin/amigo/go.cgi?query=GO:0043233&view=details) | organelle lumen | 8.76E-8 | 2.85E-6 | 7.30 (1139,24,78,12) |
| [GO:0031974](http://www.godatabase.org/cgi-bin/amigo/go.cgi?query=GO:0031974&view=details) | membrane-enclosed lumen | 8.76E-8 | 2.63E-6 | 7.30 (1139,24,78,12) |

**2) RP - Ranked list test** with p-value threshold of 10^-6^ yields:

Process


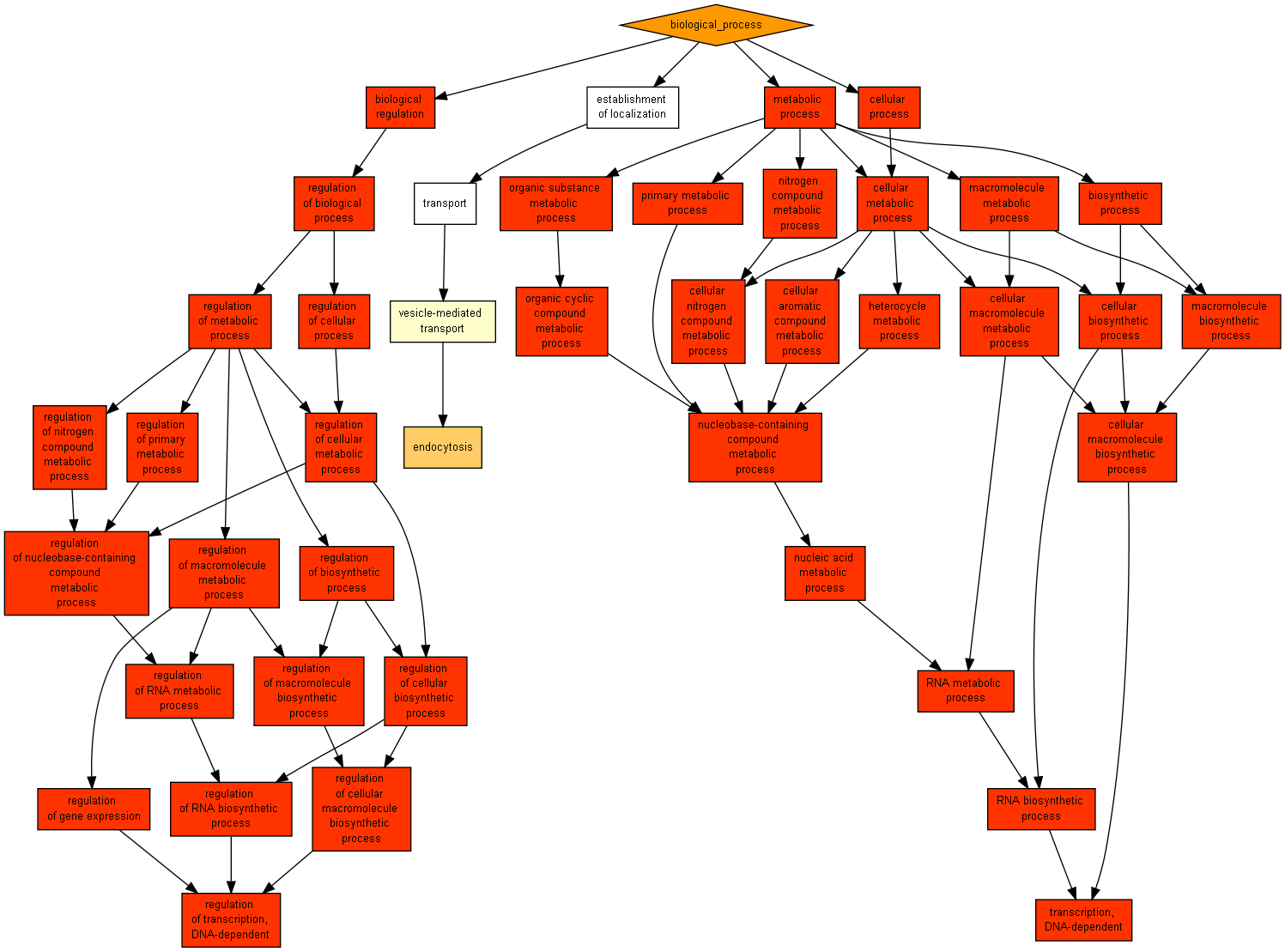


| **GO term** | **Description** | [**P-value**](http://cbl-gorilla.cs.technion.ac.il/GOrilla/t4e6hn8e/GOResultsPROCESS.html#p_value_info) | [**FDR q-value**](http://cbl-gorilla.cs.technion.ac.il/GOrilla/t4e6hn8e/GOResultsPROCESS.html#fdr_info) | [**Enrichment (N, B, n, b)**](http://cbl-gorilla.cs.technion.ac.il/GOrilla/t4e6hn8e/GOResultsPROCESS.html#enrich_info) |
| --- | --- | --- | --- | --- |
| [GO:0032774](http://www.godatabase.org/cgi-bin/amigo/go.cgi?query=GO:0032774&view=details) | RNA biosynthetic process | 3.97E-74 | 8.59E-71 | 1.79 (1139,425,498,333) |
| [GO:0006351](http://www.godatabase.org/cgi-bin/amigo/go.cgi?query=GO:0006351&view=details) | transcription, DNA-dependent | 1.19E-73 | 1.29E-70 | 1.80 (1139,420,498,330) |
| [GO:0034645](http://www.godatabase.org/cgi-bin/amigo/go.cgi?query=GO:0034645&view=details) | cellular macromolecule biosynthetic process | 2.24E-70 | 1.62E-67 | 1.77 (1139,430,498,332) |
| [GO:0009059](http://www.godatabase.org/cgi-bin/amigo/go.cgi?query=GO:0009059&view=details) | macromolecule biosynthetic process | 2.24E-70 | 1.21E-67 | 1.77 (1139,430,498,332) |
| [GO:0044249](http://www.godatabase.org/cgi-bin/amigo/go.cgi?query=GO:0044249&view=details) | cellular biosynthetic process | 8.09E-69 | 3.5E-66 | 1.74 (1139,441,498,336) |
| [GO:0009058](http://www.godatabase.org/cgi-bin/amigo/go.cgi?query=GO:0009058&view=details) | biosynthetic process | 2.6E-68 | 9.38E-66 | 1.74 (1139,442,498,336) |
| [GO:0016070](http://www.godatabase.org/cgi-bin/amigo/go.cgi?query=GO:0016070&view=details) | RNA metabolic process | 6.78E-65 | 2.1E-62 | 1.71 (1139,453,498,338) |
| [GO:0006355](http://www.godatabase.org/cgi-bin/amigo/go.cgi?query=GO:0006355&view=details) | regulation of transcription, DNA-dependent | 3.79E-64 | 1.03E-61 | 1.75 (1139,413,500,318) |
| [GO:2001141](http://www.godatabase.org/cgi-bin/amigo/go.cgi?query=GO:2001141&view=details) | regulation of RNA biosynthetic process | 3.79E-64 | 9.12E-62 | 1.75 (1139,413,500,318) |
| [GO:0051252](http://www.godatabase.org/cgi-bin/amigo/go.cgi?query=GO:0051252&view=details) | regulation of RNA metabolic process | 4.02E-64 | 8.71E-62 | 1.75 (1139,415,500,319) |
| [GO:2000112](http://www.godatabase.org/cgi-bin/amigo/go.cgi?query=GO:2000112&view=details) | regulation of cellular macromolecule biosynthetic process | 1.32E-62 | 2.6E-60 | 1.74 (1139,418,500,319) |
| [GO:0010556](http://www.godatabase.org/cgi-bin/amigo/go.cgi?query=GO:0010556&view=details) | regulation of macromolecule biosynthetic process | 4.16E-62 | 7.51E-60 | 1.73 (1139,419,500,319) |
| [GO:0031326](http://www.godatabase.org/cgi-bin/amigo/go.cgi?query=GO:0031326&view=details) | regulation of cellular biosynthetic process | 4.31E-62 | 7.18E-60 | 1.73 (1139,421,500,320) |
| [GO:0009889](http://www.godatabase.org/cgi-bin/amigo/go.cgi?query=GO:0009889&view=details) | regulation of biosynthetic process | 1.34E-61 | 2.07E-59 | 1.73 (1139,422,500,320) |
| [GO:0019219](http://www.godatabase.org/cgi-bin/amigo/go.cgi?query=GO:0019219&view=details) | regulation of nucleobase-containing compound metabolic process | 3.86E-60 | 5.57E-58 | 1.72 (1139,425,500,320) |
| [GO:0051171](http://www.godatabase.org/cgi-bin/amigo/go.cgi?query=GO:0051171&view=details) | regulation of nitrogen compound metabolic process | 3.86E-60 | 5.22E-58 | 1.72 (1139,425,500,320) |
| [GO:0090304](http://www.godatabase.org/cgi-bin/amigo/go.cgi?query=GO:0090304&view=details) | nucleic acid metabolic process | 2.22E-59 | 2.83E-57 | 1.66 (1139,467,498,339) |
| [GO:0010468](http://www.godatabase.org/cgi-bin/amigo/go.cgi?query=GO:0010468&view=details) | regulation of gene expression | 3.48E-59 | 4.19E-57 | 1.71 (1139,427,500,320) |
| [GO:0006807](http://www.godatabase.org/cgi-bin/amigo/go.cgi?query=GO:0006807&view=details) | nitrogen compound metabolic process | 2.49E-57 | 2.83E-55 | 1.64 (1139,478,498,342) |
| [GO:0034641](http://www.godatabase.org/cgi-bin/amigo/go.cgi?query=GO:0034641&view=details) | cellular nitrogen compound metabolic process | 7.49E-57 | 8.12E-55 | 1.64 (1139,477,498,341) |
| [GO:0071704](http://www.godatabase.org/cgi-bin/amigo/go.cgi?query=GO:0071704&view=details) | organic substance metabolic process | 1.21E-56 | 1.25E-54 | 1.62 (1139,486,498,345) |
| [GO:1901360](http://www.godatabase.org/cgi-bin/amigo/go.cgi?query=GO:1901360&view=details) | organic cyclic compound metabolic process | 1.78E-56 | 1.75E-54 | 1.63 (1139,480,498,342) |
| [GO:0046483](http://www.godatabase.org/cgi-bin/amigo/go.cgi?query=GO:0046483&view=details) | heterocycle metabolic process | 2E-56 | 1.88E-54 | 1.63 (1139,478,498,341) |
| [GO:0006725](http://www.godatabase.org/cgi-bin/amigo/go.cgi?query=GO:0006725&view=details) | cellular aromatic compound metabolic process | 2E-56 | 1.81E-54 | 1.63 (1139,478,498,341) |
| [GO:0006139](http://www.godatabase.org/cgi-bin/amigo/go.cgi?query=GO:0006139&view=details) | nucleobase-containing compound metabolic process | 6.7E-56 | 5.8E-54 | 1.63 (1139,475,498,339) |
| [GO:0031323](http://www.godatabase.org/cgi-bin/amigo/go.cgi?query=GO:0031323&view=details) | regulation of cellular metabolic process | 2.4E-53 | 2E-51 | 1.65 (1139,448,500,324) |
| [GO:0060255](http://www.godatabase.org/cgi-bin/amigo/go.cgi?query=GO:0060255&view=details) | regulation of macromolecule metabolic process | 7.41E-53 | 5.94E-51 | 1.65 (1139,443,500,321) |
| [GO:0080090](http://www.godatabase.org/cgi-bin/amigo/go.cgi?query=GO:0080090&view=details) | regulation of primary metabolic process | 1.91E-52 | 1.47E-50 | 1.64 (1139,446,500,322) |
| [GO:0019222](http://www.godatabase.org/cgi-bin/amigo/go.cgi?query=GO:0019222&view=details) | regulation of metabolic process | 5.26E-48 | 3.93E-46 | 1.60 (1139,463,500,325) |
| [GO:0044260](http://www.godatabase.org/cgi-bin/amigo/go.cgi?query=GO:0044260&view=details) | cellular macromolecule metabolic process | 1.68E-47 | 1.21E-45 | 1.53 (1139,528,501,355) |
| [GO:0044237](http://www.godatabase.org/cgi-bin/amigo/go.cgi?query=GO:0044237&view=details) | cellular metabolic process | 2.35E-46 | 1.64E-44 | 1.49 (1139,557,504,368) |
| [GO:0043170](http://www.godatabase.org/cgi-bin/amigo/go.cgi?query=GO:0043170&view=details) | macromolecule metabolic process | 1.36E-45 | 9.2E-44 | 1.51 (1139,540,501,358) |
| [GO:0044238](http://www.godatabase.org/cgi-bin/amigo/go.cgi?query=GO:0044238&view=details) | primary metabolic process | 1.14E-42 | 7.46E-41 | 1.47 (1139,562,503,365) |
| [GO:0008152](http://www.godatabase.org/cgi-bin/amigo/go.cgi?query=GO:0008152&view=details) | metabolic process | 7.38E-42 | 4.7E-40 | 1.45 (1139,577,504,371) |
| [GO:0050794](http://www.godatabase.org/cgi-bin/amigo/go.cgi?query=GO:0050794&view=details) | regulation of cellular process | 1.77E-29 | 1.09E-27 | 1.40 (1139,556,498,341) |
| [GO:0050789](http://www.godatabase.org/cgi-bin/amigo/go.cgi?query=GO:0050789&view=details) | regulation of biological process | 4.69E-28 | 2.82E-26 | 1.38 (1139,570,506,349) |
| [GO:0065007](http://www.godatabase.org/cgi-bin/amigo/go.cgi?query=GO:0065007&view=details) | biological regulation | 1.14E-26 | 6.66E-25 | 1.36 (1139,582,506,352) |
| [GO:0009987](http://www.godatabase.org/cgi-bin/amigo/go.cgi?query=GO:0009987&view=details) | cellular process | 4.58E-18 | 2.61E-16 | 1.21 (1139,764,506,412) |
| [GO:0008150](http://www.godatabase.org/cgi-bin/amigo/go.cgi?query=GO:0008150&view=details) | biological_process | 1.39E-8 | 7.71E-7 | 1.09 (1139,902,579,501) |
| [GO:0006897](http://www.godatabase.org/cgi-bin/amigo/go.cgi?query=GO:0006897&view=details) | endocytosis | 2.68E-7 | 1.45E-5 | 71.19 (1139,8,8,4) |

Function


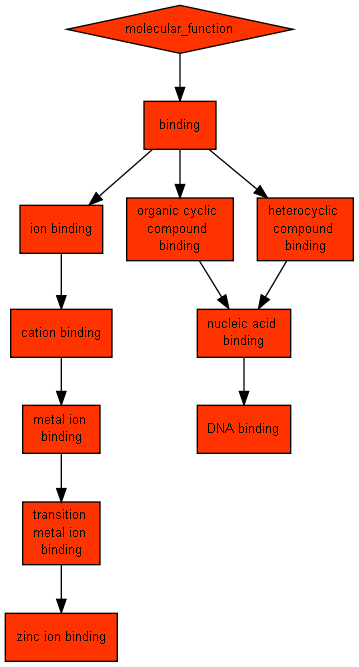


| **GO term** | **Description** | [**P-value**](http://cbl-gorilla.cs.technion.ac.il/GOrilla/t4e6hn8e/GOResultsFUNCTION.html#p_value_info) | [**FDR q-value**](http://cbl-gorilla.cs.technion.ac.il/GOrilla/t4e6hn8e/GOResultsFUNCTION.html#fdr_info) | [**Enrichment (N, B, n, b)**](http://cbl-gorilla.cs.technion.ac.il/GOrilla/t4e6hn8e/GOResultsFUNCTION.html#enrich_info) |
| --- | --- | --- | --- | --- |
| [GO:0046914](http://www.godatabase.org/cgi-bin/amigo/go.cgi?query=GO:0046914&view=details) | transition metal ion binding | 4.02E-80 | 2.48E-77 | 1.77 (1139,455,504,356) |
| [GO:0008270](http://www.godatabase.org/cgi-bin/amigo/go.cgi?query=GO:0008270&view=details) | zinc ion binding | 4.65E-80 | 1.44E-77 | 1.79 (1139,444,498,348) |
| [GO:0003676](http://www.godatabase.org/cgi-bin/amigo/go.cgi?query=GO:0003676&view=details) | nucleic acid binding | 2.12E-67 | 4.36E-65 | 1.64 (1139,520,498,372) |
| [GO:0003677](http://www.godatabase.org/cgi-bin/amigo/go.cgi?query=GO:0003677&view=details) | DNA binding | 2.53E-62 | 3.9E-60 | 1.72 (1139,434,498,326) |
| [GO:0046872](http://www.godatabase.org/cgi-bin/amigo/go.cgi?query=GO:0046872&view=details) | metal ion binding | 1.04E-59 | 1.28E-57 | 1.58 (1139,538,496,371) |
| [GO:0043169](http://www.godatabase.org/cgi-bin/amigo/go.cgi?query=GO:0043169&view=details) | cation binding | 2.65E-59 | 2.73E-57 | 1.58 (1139,539,496,371) |
| [GO:0043167](http://www.godatabase.org/cgi-bin/amigo/go.cgi?query=GO:0043167&view=details) | ion binding | 2.65E-59 | 2.34E-57 | 1.58 (1139,539,496,371) |
| [GO:1901363](http://www.godatabase.org/cgi-bin/amigo/go.cgi?query=GO:1901363&view=details) | heterocyclic compound binding | 2.24E-50 | 1.73E-48 | 1.48 (1139,592,504,388) |
| [GO:0097159](http://www.godatabase.org/cgi-bin/amigo/go.cgi?query=GO:0097159&view=details) | organic cyclic compound binding | 2.24E-50 | 1.54E-48 | 1.48 (1139,592,504,388) |
| [GO:0005488](http://www.godatabase.org/cgi-bin/amigo/go.cgi?query=GO:0005488&view=details) | binding | 1.29E-22 | 7.93E-21 | 1.20 (1139,832,506,445) |
| [GO:0003674](http://www.godatabase.org/cgi-bin/amigo/go.cgi?query=GO:0003674&view=details) | molecular_function | 7.94E-14 | 4.45E-12 | 1.11 (1139,935,567,516) |

Component


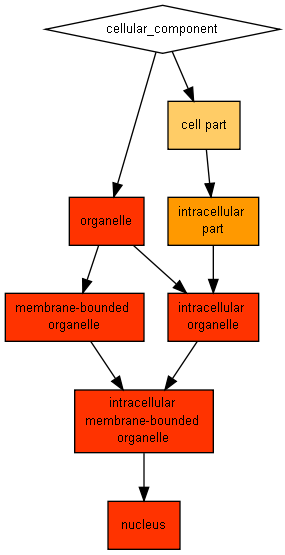


| **GO term** | **Description** | [**P-value**](http://cbl-gorilla.cs.technion.ac.il/GOrilla/t4e6hn8e/GOResultsCOMPONENT.html#p_value_info) | [**FDR q-value**](http://cbl-gorilla.cs.technion.ac.il/GOrilla/t4e6hn8e/GOResultsCOMPONENT.html#fdr_info) | [**Enrichment (N, B, n, b)**](http://cbl-gorilla.cs.technion.ac.il/GOrilla/t4e6hn8e/GOResultsCOMPONENT.html#enrich_info) |
| --- | --- | --- | --- | --- |
| [GO:0005634](http://www.godatabase.org/cgi-bin/amigo/go.cgi?query=GO:0005634&view=details) | nucleus | 1.79E-42 | 6.99E-40 | 1.48 (1139,549,501,358) |
| [GO:0043231](http://www.godatabase.org/cgi-bin/amigo/go.cgi?query=GO:0043231&view=details) | intracellular membrane-bounded organelle | 2.18E-36 | 4.25E-34 | 1.40 (1139,601,506,375) |
| [GO:0043227](http://www.godatabase.org/cgi-bin/amigo/go.cgi?query=GO:0043227&view=details) | membrane-bounded organelle | 2.18E-36 | 2.84E-34 | 1.40 (1139,601,506,375) |
| [GO:0043226](http://www.godatabase.org/cgi-bin/amigo/go.cgi?query=GO:0043226&view=details) | organelle | 1.46E-29 | 1.42E-27 | 1.34 (1139,639,506,381) |
| [GO:0043229](http://www.godatabase.org/cgi-bin/amigo/go.cgi?query=GO:0043229&view=details) | intracellular organelle | 1.46E-29 | 1.14E-27 | 1.34 (1139,639,506,381) |
| [GO:0044424](http://www.godatabase.org/cgi-bin/amigo/go.cgi?query=GO:0044424&view=details) | intracellular part | 1.79E-8 | 1.16E-6 | 1.16 (1139,830,370,312) |
| [GO:0044464](http://www.godatabase.org/cgi-bin/amigo/go.cgi?query=GO:0044464&view=details) | cell part | 1.14E-7 | 6.37E-6 | 1.14 (1139,863,370,319) |
